# Glycaemic control among type 2 diabetes patients in sub-Saharan Africa from 2012 to 2022: a systematic review and meta-analysis

**DOI:** 10.1186/s13098-022-00902-0

**Published:** 2022-09-20

**Authors:** Jean-Pierre Fina Lubaki, Olufemi Babatunde Omole, Joel Msafiri Francis

**Affiliations:** 1 Department of Family Medicine and Primary Care, University of the Witwatersrand, Johannesburg, South Africa; 2 Department of Family Medicine and Primary Care, Protestant University of Congo, Kinshasa, Democratic Republic of the Congo

**Keywords:** Systematic review, Meta-analysis, Prevalence, Factors, Glycaemic control, Type 2 diabetes, Sub-Saharan Africa

## Abstract

**Background:**

There is an increased burden of diabetes globally including in sub-Saharan Africa. The literature shows that glycaemic control among type 2 diabetes patients is poor in most countries in sub-Saharan Africa. Understanding the factors influencing glycaemic control in this region is therefore important to develop interventions to optimize glycaemic control. We carried out a systematic review to determine the prevalence and factors associated with glycaemic control in sub-Saharan Africa to inform the development of a glycaemic control framework in the Democratic Republic of the Congo.

**Methods:**

We searched five databases (African Index Medicus, Africa-Wide Information, Global Health, PubMed, and Web of Science) using the following search terms: type-2 diabetes, glycaemic control, and sub-Saharan Africa. Only peer-reviewed articles from January 2012 to May 2022 were eligible for this review. Two reviewers, independently, selected articles, assessed their methodological quality using Joanna Briggs checklists, and extracted data. A meta-analysis was performed to estimate the prevalence of glycaemic control. Factors associated with glycaemic control were presented as a narrative synthesis due to heterogeneity as assessed by the I^2^.

**Results:**

A total of 74 studies, involving 21,133 participants were included in the review. The pooled prevalence of good glycaemic control was 30% (95% CI:27.6–32.9). The glycaemic control prevalence ranged from 10–60%. Younger and older age, gender, lower income, absence of health insurance, low level of education, place of residence, family history of diabetes, longer duration of diabetes, pill burden, treatment regimen, side effects, use of statins or antihypertensives, alcohol consumption, smoking, presence of comorbidities/complications, and poor management were associated with poor glycaemic control. On the other hand, positive perceived family support, adequate coping strategies, high diabetes health literacy, dietary adherence, exercise practice, attendance to follow-up, and medication adherence were associated with good glycaemic control.

**Conclusion:**

Suboptimal glycaemic control is pervasive among patients with type-2 diabetes in sub-Saharan Africa and poses a significant public health challenge. While urgent interventions are required to optimize glycaemic control in this region, these should consider sociodemographic, lifestyle, clinical, and treatment-related factors. This systematic review and meta-analysis protocol is registered in PROSPERO under CRD 42021237941.

**Supplementary Information:**

The online version contains supplementary material available at 10.1186/s13098-022-00902-0.

## Background

The prevalence of type 2 diabetes mellitus has been increasing worldwide, with low and middle-income countries bearing the brunt of this growth in terms of morbidity, mortality, and economic costs [1, 2]. As such, Africa has been experiencing the greatest increase of all the World Health Organization (WHO) regions. The epidemiological transition due to the adoption of the Western lifestyle and urbanization, among other things, has played a major role in the progression of diabetes [3]. The growing burden of diabetes has been a barrier to the wellness of families and the effectiveness of the health system.

One of the main goals of diabetes mellitus management is to achieve glycaemic control to delay or prevent the onset of diabetes complications. Worldwide, only approximately 50% of patients achieve glycaemic control [4] and in sub-Saharan Africa (SSA), glycaemic control rates are generally poor. In sub-Saharan Africa, the proper management of diabetes faces numerous challenges including inadequate resources, coexisting traditional health priorities, ill-preparedness for chronic disease management and low health insurance coverage [5].

Glycaemic control represents an emergency to alleviate the burden of the disease in sub-Saharan Africa [6]. The design and implementation of effective glycaemic control strategies require accurate knowledge of the factors underlying glycaemic control to enable the identification of effective interventions. The factors associated with poor glycaemic control are numerous and vary in importance depending on the population [7, 8]. Empirical evidence suggests that higher socioeconomic status, greater dietary knowledge, and higher self-efficacy and empowerment improve glycaemic control [9]. Factors driving poor glycaemic control include patients, diabetes disease, treatment, health system, and physician-related factors [8, 10]. However, there is a paucity of literature on factors that influence glycaemic control in the sub-Saharan region. Therefore, this systematic review aims to determine the prevalence and factors associated with glycaemic control among type 2 diabetes patients in sub-Saharan Africa. The review will comprise all articles on glycaemic control among patients with type 2 diabetes from January 2012 to May 2022 to have enough studies to have a broad view of the phenomenon.

## Methods

The protocol of this systematic review and meta-analysis was registered on PROSPERO with reference CRD 41021237941. The Preferred Reporting Items for Systematic reviews and Meta-Analyses (PRISMA) guidelines were used to report the entire process of this systematic review [11].

### Eligibility criteria

Eligible studies were those that reported glycaemic control in persons with type 2 diabetes mellitus in sub-Saharan Africa. Only peer-reviewed articles were eligible to ensure the inclusion of valid research and avoid falsified data. Data from January 2012 to May 2022 without language restrictions were included. As we planned to estimate the prevalence and identify factors associated with glycaemic control, the following types of study designs were considered: randomized controlled trials, quasi-experimental trials, cohort studies, case–control studies, and cross-sectional studies. Only studies that reported a multivariate analysis were included in the systematic review.

### Information sources

The search was conducted in five databases: African Index Medicus, Africa-Wide Information, Global Health, PubMed, and Web of Science. In addition, the reference lists of the selected articles were reviewed for any other eligible articles. The last search date was 02 May 2022.

### Search strategy

The search term domains were “Type 2 diabetes mellitus”, “glycaemic control”, and “sub-Saharan Africa”. Additional file 1: Table S1 presents the search strategy at the level of the five databases.

### Selection process

Two investigators (JPF and JMF) independently reviewed the studies using the eligibility criteria and selected studies for inclusion in the review. The first investigator (JPF) reviewed all articles, and the second investigator (JMF) randomly assessed 10% of the selected articles. Differences in selection were assessed by consensus. The software used for the selection and recording of decisions was EndNote 20.

### Data extraction

Data from eligible studies were captured using a Microsoft Excel file. The first investigator (JPF) performed data extraction on all the articles and the second investigator (JMF) randomly assessed 10% of the extracted information. Any differences of opinion between individual judgments were resolved through consensus. For the metaanalysis of the proportions of glycaemic control, we contacted eight authors for missing information and clarification. One provided us with its study dataset, and another showed us how to access the information needed to calculate the glycaemic control.

### Study variables

#### Main outcomes

Reported glycaemic control: pooled prevalence of samples reported to have glycaemic control.

Exposure: reported independent risk factors for glycaemic control.

#### Data items

The following information was extracted from the studies: the last name of the first author, study type, publication year, country, study population, total sample size, glycaemic level assessment method, glycaemic control definition, number or proportion of persons with good glycaemic control, factors associated with glycaemic control, and measure of association for glycaemic control.

### Study risk of bias assessment

The assessment of the methodological quality of the selected studies was performed by two reviewers (JPF and JMF) using the Joanna Briggs Institute Critical Appraisal Tools [12]; any differences of opinion between the two reviewers were resolved by consensus. The Joanna Briggs Institute Critical Appraisal Tools were used to classify each selected study as good, moderate, or poor regarding the risk of bias. The percentage of "Yes" votes was equal to or less than 50%, 51–80%, and more than 80% for poor, moderate, or good quality respectively [13]. The assessment of an item was marked "Yes" if the description met the criteria set for the assessment, otherwise, the assessment was "No". If the description was insufficient, the assessment was "Unknown".

### Synthesis methods

To estimate the overall prevalence of glycaemic control, we carried out a meta-analysis with the random-effects model of the proportions of good glycaemic control. The statistical software used was Stata 17.0 [14]. The data needed for analysis are summarized in Additional file 3: Table S3. Only 51 studies were eligible for the estimation of the prevalence of glycaemic control as randomized control trials, quasi-experimental and case–control studies were excluded. We observed high heterogeneity of the studies as shown by the I^2^ [15], and therefore reported the pooled prevalence estimate and the glycaemic control patterns in sub-Saharan Africa. Forest plots were used to visually display the results of individual studies and the syntheses. We explored heterogeneity by performing subgroups analysis of the prevalence by region of sub-Saharan Africa (Eastern, Western, Central, Southern), study type (case–control, cohort, cross-sectional, quasi-experimental, randomized control trial), and method (glycosylated haemoglobin, glycaemia) used to assess the control.

To assess the factors driving glycaemic control, due to the heterogeneity of the studies, we performed a narrative synthesis of reported factors. Each reported factor is presented with the studies in which it was assessed, and the measure of association —and its 95% confidence interval—with glycaemic control found in each study is reported. The factors were categorized into six groups: sociodemographic, lifestyle, clinical, treatment modalities, adherence, and interventions.

## Results

### Study selection

**Fig. 1:**
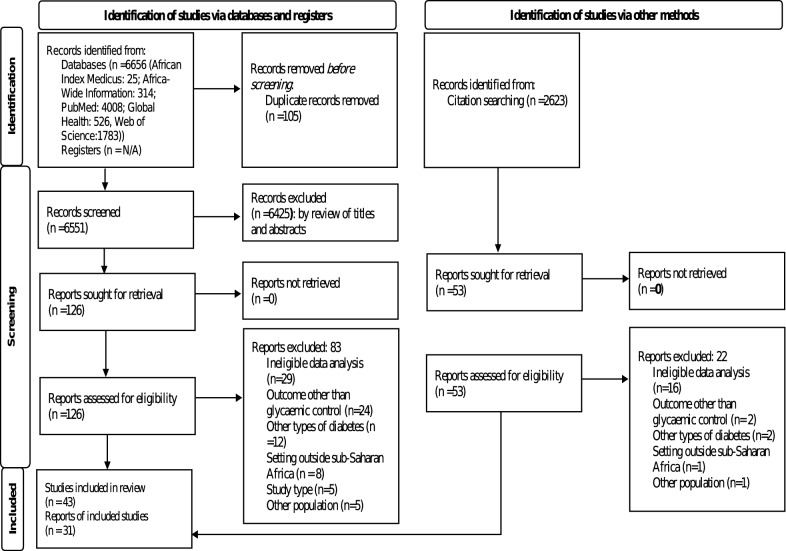
Selection of studies

We retrieved 6656 publications from the information sources. A total of 105 publications were removed due to duplication. Of the remaining 6551 articles, 6425 were excluded after the titles and abstracts were reviewed, and 126 were retained for full-text evaluation based on the inclusion criteria. Of the 126, eighty-three articles were excluded after full-text review and 43 articles were retained. An additional search of the reference lists of selected articles yielded 2623 publications. We selected 53 publications for full-text evaluation according to the selection criteria. Of these, 22 were excluded because they did not meet inclusion criteria, and 31 articles were retained. The total number of included studies was 74 articles by both search strategies. The selection process is summarized in the PRISMA flow diagram (Fig. 1). The characteristics of the excluded studies and the reason(s) for exclusion are summarized in Additional file 2: Table S2.

### General characteristics of the included studies

A total of 74 studies reporting on 21,133 patients with type 2 diabetes were included in the review. The studies were conducted in 16 sub-Saharan African countries, with Ethiopia (n = 26, 35.1%) being the most represented, followed by South Africa (n = 11, 14.9%) and Nigeria (n = 10, 13.5%). The majority of the studies (n = 51, 68.9%) were conducted in the last five years (2017-2022). Of the 74 studies selected, 55 (74.3%) were cross-sectional studies, ten (13.5%) were randomized controlled studies, four (5.4%) were quasi-experimental studies, three (4.1%) were case–control studies, and two (2.7%) were cohort studies. The general characteristics of the included studies are presented in Table 1.

**Table 1:** General characteristics of included studies

First author last name	Study setting	Study type	Publication year	Study population	Sample size	Glycaemic level assessment method
Achila [16]	Eritrea	Cross-sectional	2020	Type 2 diabetes; 20–88 years	309	HbA1c
Adejumo [17]	Nigeria	Cross-sectional	2012	Type 2 diabetes with normal renal function; ≥ 18 years	144	HbA1c
Adeniyi [18]	South Africa	Cross-sectional	2016	Type 2 diabetes; ≥ 30 years at diagnostic of diabetes	327	HbA1c
Afolabi [19]	Nigeria	Cross-sectional	2018	Type 2 diabetes; 40–80 years	80	HbA1c
Akabwai [20]	Uganda	Cross-sectional	2016	Type 2 diabetes; ≥ 18 years	280	HbA1c
Akpalu [21]	Ghana	Cross-sectional	2018	Type 2 diabetes; 30–65 years	400	HbA1c
Anioke [22]	Nigeria	Cross-sectional	2019	Type 2 diabetes; ≥ 30 years	140	HbA1c
Anyanwu [23]	Nigeria	Randomized controlled trial	2016	Type 2 diabetes with poor glycemic control and vitamin D deficiency; 35–65 years	42	HbA1c
Assah [24]	Cameroon	Randomized controlled trial	2015	Type 2 diabetes	192	HbA1c
Ayele [25]	Ethiopia	Cross-sectional	2019	Type 2 diabetes; > 18 years	275	FBG
Belay [26]	Ethiopia	Cross-sectional	2017	Type 2 diabetes; 18–80 years	188	FPG
BeLue [27]	Senegal	Cross-sectional	2016	Type 2 diabetes	106	HbA1c
Biadgo [28]	Ethiopia	Cross-sectional	2018	Type 2 diabetes	159	FBS
Biru [29]	Ethiopia	Cross-sectional	2017	Type 2 diabetes; ≥ 18 years	322	FBG
Blum [30]	DR Congo	Cross-sectional	2019	Type 2 diabetes; ≥ 18 years	319	HbA1c
Botchway [31]	Ghana	Cross-sectional	2021	Type 2 diabetes; ≥ 18 years	254	HbA1c
Camara [32]	Cameroon and Guinea	Cross-sectional	2015	Type 2 diabetes; ≥ 16 years	1267	HbA1c
Dagnew [33]	Ethiopia	Comparative Cross-sectional	2017	Type 2 diabetes and healthy relatives; ≥ 30 years	210	FBG
Demoz [34]	Ethiopia	Cross-sectional	2019	Type 2 diabetes; ≥ 18 years	357	HbA1c and FBG
Doglikuu [35]	Ghana	Cross-sectional	2021	Type 2 diabetes; ≥ 18 years	530	HbA1c
Eticha [36]	Ethiopia	Cross-sectional	2016	Type 2 diabetes; ≥ 18 years	384	HbA1c
Ezema [37]	Nigeria	Randomized trial	2014	Type 2 diabetes; 40–55 years	54	FBG
Fayehun [38]	Nigeria	Randomized trial	2018	Type 2 diabetes; 33–64 years	46	HbA1c
Fekadu [39]	Ethiopia	Cross-sectional	2019	Type 2 diabetes; 18–86 years	228	Mean of FBG records from the last three clinic visits
Fseha [40]	Ethiopia	Cross-sectional	2017	Type 2 diabetes; 22–60 years	200	Three-month average FBG
Gathu [41]	Kenya	Randomized controlled trial	2018	Sub-optimally controlled type 2 diabetes; 18–65 years	140	HbA1c
Gebremedhin [42]	Ethiopia	Cross-sectional	2019	Type 2 diabetes; ≥ 18 years	267	FBG
Hailu [43]	Ethiopia	Controlled before-and-after study	2018	Type 2 diabetes; > 30 years	220	HbA1c
Id [44]	Ethiopia	Cross-sectional	2021	Type 2 diabetes; > 18 years	394	FBS
Inih [45]	Nigeria	Cross-sectional	2018	Type 2 diabetes; men	150	HbA1c
Kalain [46]	South Africa	Cross-sectional	2020	Type 2 diabetes; ≥ 18 years	200	HbA1c
Kamuhabwa [47]	Tanzania	Cross-sectional	2014	Type 2 diabetes; ≥ 18 years	469	FBG
Kassahun [48]	Ethiopia	Cross-sectional	2016	Type 2 diabetes; ≥ 18 years	309	Mean of at least four months FBG readings
Kefale [49]	Ethiopia	Cross-sectional	2019	Type 2 diabetes; ≥ 18 years	169	mean of at least three-month FBG measurements
Khoza [50]	South Africa	Case–control	2018	Type 2 diabetes; > 30 years	320	HbA1c and glucose
Kimando [51]	Kenya	Cross-sectional	2017	Type 2 diabetes without overt complications; ≥ 30 years	385	HbA1c
Maharaj [52]	Nigeria	Randomized controlled trial	2016	Non-insulin dependent type 2 diabetes; 30–58 years	90	HbA1c, FPG
Mamo [53]	Ethiopia	Case–control	2019	Type 2 diabetes with poor glycemic control (cases) and without good glycemic control (controls); > 18 years	410	FBG
Mash [54]	South Africa	Quasi-experimental study	2014	Type 2 diabetes; > 18 years	1570	HbA1c
Mash [55]	South Africa	Pragmatic Randomized controlled trial	2016	Type 2 diabetes	600	HbA1c
Mashele [56]	South Africa	Cross-sectional	2019	Type 2 diabetes; 35–74 years	176	HbA1c
Mayet [57]	South Africa	Descriptive retrospective study	2012	Poorly controlled insulin-requiring type 2 diabetes	131	HbA1c
Mobula [58]	Ghana	Cross-sectional	2018	Hypertensive and Type 2 diabetes; ≥ 18 years	1226	HbA1c
Mohamed [59]	Sudan	Case–control	2013	Type 2 diabetes and non-diabetic controls	457	HbA1c
Mohammed [60]	Ethiopia	Cross-sectional	2020	Type 2 diabetes	307	FBG
Mphwanthe [61]	Malawi	Cross-sectional	2020	Type 2 diabetes; ≥ 25 years	428	HbA1c
Mphwanthe [62]	Malawi	Cross-sectional	2020	Type 2 diabetes	428	HbA1c
Muchiri [63]	South Africa	Randomized controlled trial	2016	Type 2 diabetes; 40–70 years	82	HbA1c
Mwavua [64]	Kenya	Cross-sectional	2016	Type 2 diabetes; ≥ 18 years	200	HbA1c
Mwita [65]	Botswana	Cross-sectional	2019	Type 2 diabetes; ≥ 18 years	500	HbA1c
Noor [66]	Sudan	Cross-sectional	2017	Type 2 diabetes	387	HbA1c
Omar [67]	Sudan	Cross-sectional	2018	Type 2 diabetes; ≥ 18 years	339	HbA1c
Osuji [68]	Nigeria	Cross-sectional	2018	Type 2 diabetes; ≥ 18 years	316	HbA1c
Otieno [69]	Kenya	Cross-sectional	2017	Type 2 diabetes; ≥ 30 years	220	HbA1c
Oyewole [70]	Nigeria	Cross-sectional	2019	Type 2 diabetes; ≥ 21 years	162	FBG and HbA1c
Rambiritch [71]	South Africa	A 12-week, prospective, single-center, open-label, dose-escalation study	2014	Poorly controlled type 2 diabetes requiring oral antidiabetic medications; > 20 years	22	FBG
Ramkisson [72]	South Africa	Cross-sectional	2016	Type 2 diabetes; ≥ 18 years	401	HbA1c
Rwegerera [73]	Botswana	Cross-sectional	2019	Type 2 diabetes	368	HbA1c
Sarfo-Kantanka [74]	Ghana	Case–control	2017	Type 2 diabetes; 40–80 years	612	FBG and HbA1c
Shimels [75]	Ethiopia	Cross-sectional	2018	Type 2 diabetes; ≥ 18 years	361	FPG
Siddiqui [76]	South Africa	Prospective observational study	2018	Type 2 diabetes; 18–65 years	95	HbA1c
Tefera [77]	Ethiopia	Cross-sectional	2020	Type 2 diabetes; ≥ 18 years	400	FPG
Tekalegn [78]	Ethiopia	Cross-sectional	2018	Type 2 diabetes; ≥ 15 years	412	FBG
Teklay [79]	Ethiopia	Cross-sectional	2013	Type 2 diabetes; ≥ 18 years	267	Mean of the last four FBG readings
Thuita [80]	Kenya	Cross-sectional	2019	Type 2 diabetes; 20–79 years	153	HbA1c
Thuita [81]	Kenya	Randomized controlled trial	2020	Type 2 diabetes; 20–79 years	153	HbA1C and FBG
Tsobgny-Tsague [82]	Cameroon	Randomized controlled trial	2018	Type 2 diabetes with poor glycaemic control and moderate to severe chronic periodontitis	34	HbA1c
Woldu [83]	Ethiopia	Cross-sectional	2014	Type 2 diabetes	102	FBG
Yan [84]	Mozambique	Randomized controlled trial	2014	Type 2 diabetes; 40–70 years	41	HbA1c
Yigazu [85]	Ethiopia	Cross-sectional	2017	Type 2 diabetes; 18–80 years	174	average FBG
Yimam [86]	Ethiopia	Cross-sectional	2020	Type 2 diabetes with hypertension; ≥ 18 years	300	Mean FBG level over three months Consecutive measurements
Yosef [87]	Ethiopia	Cross-sectional	2021	Type 2 diabetes	245	Mean FBG over three consecutive visits
Abera [88]	Ethiopia	Cross-sectional	2022	Type 2 diabetes	325	HbA1c
Abebe [89]	Ethiopia	Prospective observational study	2022	Type 2 diabetes	138	Mean FBG level over three months consecutive measurements

*HbA1c* Haemoglobin A1c, *FBG* Fasting blood glucose, *FBS* Fasting blood sugar, *FPG* Fasting plasma glucose

### Assessment of risk of bias

Of the 74 studies selected for the review, only 14 (18.9%) were assessed as being of good quality, 54 (73.0%) were of moderate quality, and six (8.1%) were of poor quality. Additional file 4: Tables S4, Additional file 5: Table S5, Additional file 6: Table S6, Additional file 7: Table S7, Additional file 8: Table S8, Additional file 9 detail the assessment of study methodological quality. Of the 55 cross-sectional studies, only four (7.3%) were able to formally identify confounding factors, while ten (1 8.2%) reported the method used to address confounding factors. In the four quasi-experimental studies, one study (25.0%) did not measure the outcomes consistently or in a reproducible way. In two of the ten randomized controlled trials (20.0%), participants and treatment providers were not blinded to treatment allocation nor were the staff members assessing outcomes blinded to treatment allocation. Moreover, for one of these two studies, the treatment groups were not similar at baseline. For two of the three case–control studies, confounding factors were not identified, and for one study, cases and controls were mismatched.

### Assessment of glycaemic control

Glycaemic control was assessed by glycosylated haemoglobin in 43(58.1%) studies, fasting blood glucose in 25 (33.8%) studies and a combination of both methods in 6(8.1%) studies. The cut-off points for good glycaemic control varied across studies and were: HbA1c < 7%, HbA1c ≤ 7%, HbA1c < 8%, HbA1c < 53 mmol/mol, FBG: 70–130 mg/dL, FBG < 126 mg/dL, FBG ≤ 126 mg/dL, FBG: 70–130 mg/dL, FBG < 154 mg/dL, FBS ≤ 130 mg/dl, FPG: 100–130 mg/dL, FBG: 4–7 mmol/L, FBG ≤ 130 mg/dL or 7. 2 mmol/L.

### Prevalence of glycaemic control

**Fig.2:**
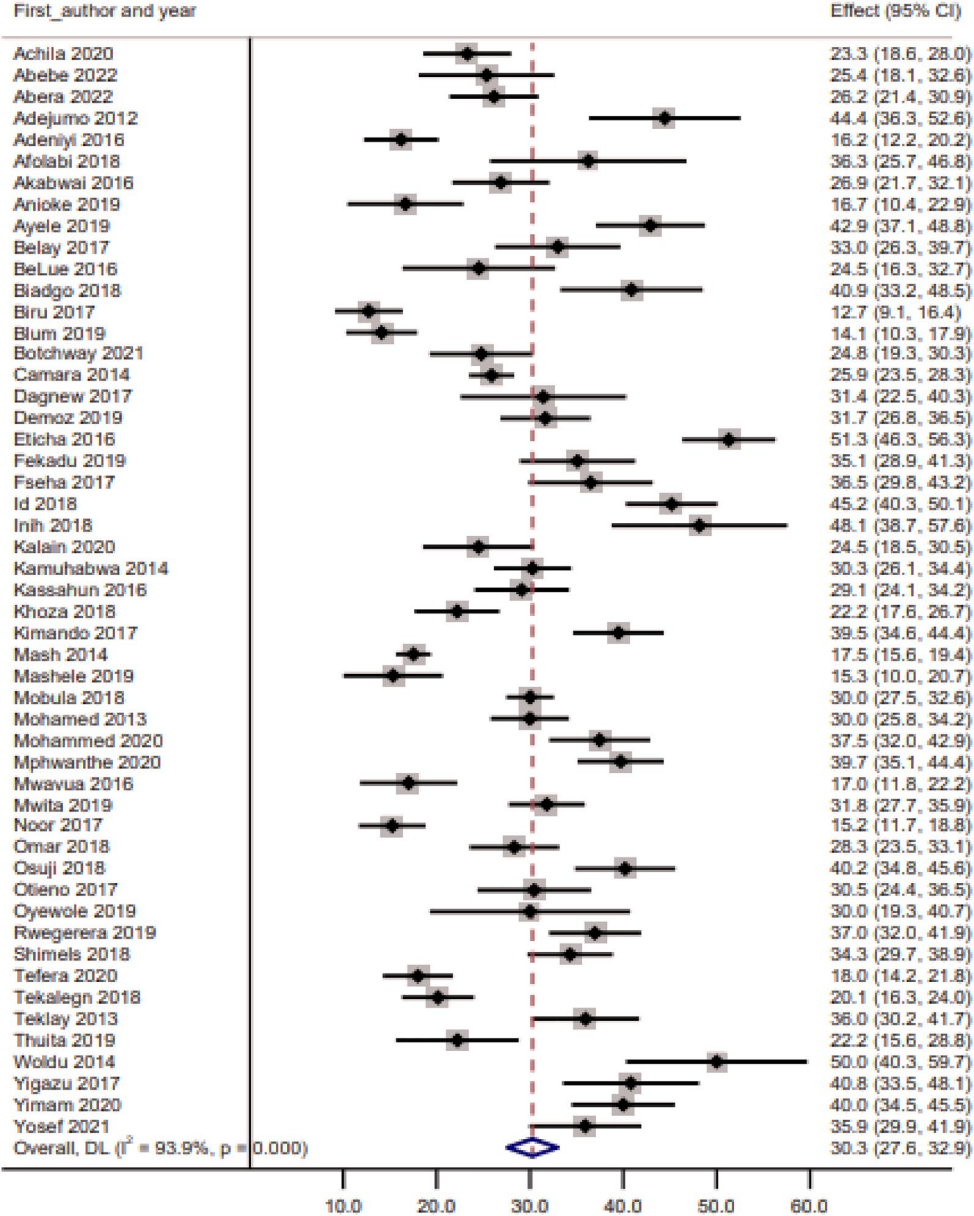
Prevalence of glycaemic control in sub-Sharan Africa

**Fig.3:**
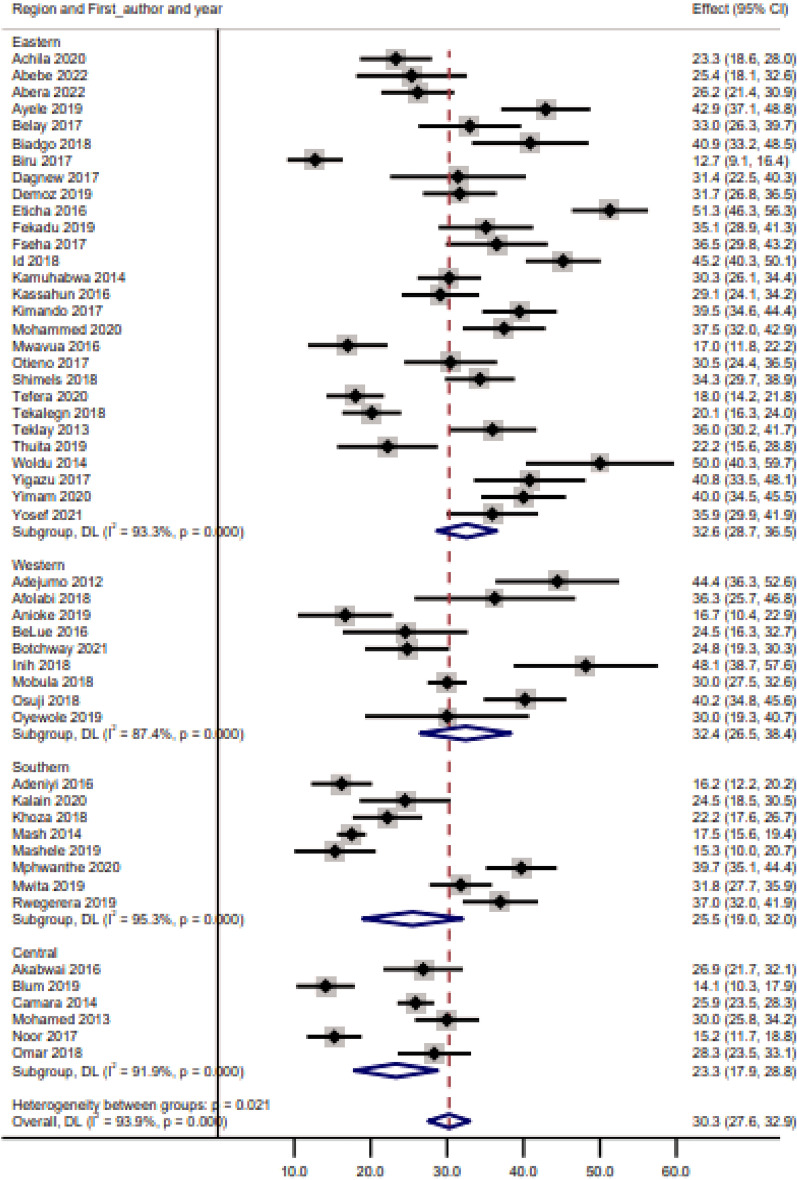
Prevalence of glycaemic control by sub-Saharan Africa regions

The estimated pooled prevalence of good glycaemic control in sub-Saharan Africa was 30.3% (95% CI: 27.6–32.9). The analysis showed considerable heterogeneity (I^2^: 93.9%, p < 0.001), and glycaemic control prevalence ranged from 10 to 60% (Fig. 2). The subgroup analysis by region showed that most of the studies in the Central (n = 5, 83.3%) and the Southern (n = 5, 62.5%) regions had a prevalence of glycaemic control of < 30% while most of the studies in the Eastern region had a prevalence of glycaemic control > 30% (Fig. 3).

### Factors associated with glycaemic control

The reported sociodemographic, lifestyle, clinical, adherence, treatment factors, and reported glycaemic control optimization interventions factors are summarized in Tables 2, 3, 4, 5, 6, 7.

**Table 2:** Sociodemographic factors and glycaemic control in sub-Saharan Africa

First author name	Year	Study design	Study setting	Study population	Sample size	Measurement of glycemic control	Definition of glycaemic control	Generic factor	Specific factors	Measure of association	Point estimate	Lower bound	Upper bound	Association with glycemic control
BeLue [27]	2016	Cross-sectional	Senegal	Type 2 diabetes	106	HbA1c	Glycemic control (HbA1c < 7%)	Age	Increasing age	ß coefficient	− 0.06	− 0.08	− 0.04	No significant association between age and glycaemic control
Botchway [31]	2021	Cross-sectional	Ghana	Type 2 diabetes; ≥ 18 years	254	HbA1c	No threshold			ß coefficient	− 0.05	− 0.07	− 0.03	Significant negative association between age and HbA1c
Demoz [34]	2019	Cross-sectional	Ethiopia	Type 2 diabetes; ≥ 18 years	357	FBG	Adequate control (average fasting blood glucose 70–130 mg/dL or HbA1c < 7%); poor control (average fasting blood glucose > 130 or < 70 mg/dL or HbA1c > 7%)			Odds Ratio	1.57	1.11	2.31	Age was not a predictor significantly associated with poor glycaemic control
Mobula [58]	2018	Cross-sectional	Ghana	Hypertensive and Type 2 diabetes; ≥ 18 years	1226	HbA1c	Poor control (HBA1c ≥ 7%)			Odds Ratio	0.97	0.96	0.98	Increasing age had significant association with good glycaemic control
Mphwanthe [61]	2020	Cross-sectional	Malawi	Type 2 diabetes; ≥ 25 years	428	HbA1c	Poor control (HbA1c ≥ 8%)			ß coefficient	− 0.065	− 0.80	− 0.050	Increasing age in years showed a negative significant association with HbA1c
Anioke [22]	2019	Cross-sectional	Nigeria	Type 2 diabetes; ≥ 30 years	140	HbA1c	Good control (HbA1c < 7%); poor control (HbA1c ≥ 7%)		Age ≥ 65 years	Odds Ratio	5.00	1.19	20.96	Being an elderly was five times more likely to show poor glycaemia than non-elderly
Osuji [68]	2018	Cross-sectional	Nigeria	Type 2 diabetes; ≥ 18 years	316	HbA1c	Good (HbA1c < 7%); poor (HbA1c ≥ 7%)			Odds Ratio	0.538	0.111	2.607	No significant association between glycaemic control and age group ≥ 65 years
											0.375	0.075	1.875	No significant association between glycaemic control and age group 45–64 yrs
Otieno [69]	2017	Cross-sectional	Kenya	Type 2 diabetes; ≥ 30 years	220	HbA1c	Good control (HbA1c ≤ 7%); poor/suboptimal (HbA1c > 7%)			Odds Ratio	0.9	0.3	3.6	No influence of age ≥ 65 years in determining glycaemic control in patients with depression
											1.5	0.6	3.6	No influence of age ≥ 65 years in determining glycaemic control in patients without depression
Biru [29]	2017	Cross-sectional	Ethiopia	Type 2 diabetes; ≥ 18 years	322	FBG	Good control (FBG ≤ 110 mg/dL)		41–50 years	Odds Ratio	2.82	0.62	12.74	Age 41–50 years was not associated significantly associated with glycaemic control
									51–60 years		3.22	0.76	13.70	Age 51–60 years was not associated significantly associated with glycaemic control
									61–70 years		8.32	1.76	39.35	Age 61–70 years was associated with good glycaemic control
									≥ 71 years		31.30	4.07	240.90	Age ≥ 71 years was associated with good glycaemic control
Woldu [83]	2014	Cross-sectional	Ethiopia	Type 2 diabetes	102	FBG	Poor glycaemic control (FBG level of > 126 mg/dl)		41–50 years	Odds Ratio	0.01	0.000	1.1144	Being in the age group 41–50 years was significantly associated with poor glycaemic control
									51–60 years		0.01	0.000	0.749	Being in the age group 51–60 years was significantly associated with poor glycaemic control
									61–70 years		0.01	0.000	0.395	Being in the age group 61–70 years was significantly associated with poor glycaemic control
									≥ 71 years		0.1	0.002	5.794	Being in the age group > 71 years was significantly associated with poor glycaemic control
Camara [32]	2014	Cross-sectional	Cameroon and Guinea	Type 2 diabetes mellitus; ≥ 16 years	1267	HbA1c	Good control (HbA1C < 7.0% or < 53 mmol/mol)		Age ˂ 65 years	Odds Ratio	1.39	1.19	20.96	Age of less than 65 years was signifcantly associated with poor glycaemic control
Eticha [36]	2016	Cross-sectional	Ethiopia	Type 2 diabetes; ≥ 18 years	384	HbA1c	Good control (HbA1c < 7%); poor control (HbA1c ≥ 7%)		˂ 50 years	Odds Ratio	3.0	1.2	7.4	Age of less than 50 years was significantly associated with poor glycaemic control
									50–59 years		4.7	2.0	11.0	Age between 50 and 59 years was significantly associated with poor glycaemic control
Fekadu [39]	2019	Cross-sectional	Ethiopia	Type 2 diabetes; 18–86 years	228	FBG	Good control (FBG: 70–130 mg/dL); poor control (< 70 mg/dL and > 130 mg/dL)		< 21 years	Odds Ratio	1.32	0.09	38.04	No significant association between age < 21 years and glycaemic control
									21–40 years		1.21	0.03	1.49	No significant association between age 21–40 years and glycaemic control
									41–60 years		2.01	0.04	0.06	Significant association between age 41–60 years and poor glycaemic control
Kimando [51]	2017	Cross-sectional	Kenya	Type 2 diabetes without overt complications; ≥ 30 years	385	HbA1c	Sub-optimal control (HbA1c > 7.0%)		˃ 50 years	Odds Ratio	0.8	0.4	1.5	Being aged more than 50 years was not significantly associated with poor glycaemic control
Mwita [65]	2019	Cross-sectional	Botswana	Type 2 diabetes; ≥ 18 years	500	HbA1c	Optimal glycaemic (HbA1c < 7%)		≤ 50 years	Odds Ratio	5.79	1.08	31.14	Age equal or less than 50 years was significantly associated with optimal glycaemic control
Rwegerera [73]	2019	Cross-sectional	Botswana	Type 2 diabetes	368	HbA1c	desirable (< 7%); suboptimal (7–9%); poor (≥ 9%)		36–50 years	Odds Ratio	2.03	0.3	13.69	This age group was not significantly associated with glycaemic control
									51–65 years		4.32	0.63	29.8	This age group was not associated with desirable glycaemic control but significantly associated with suboptimal glycaemic control
									≥ 66 years		11.7	1.4	97.69	This age group was also significantly associated with both desirable and suboptimal glycaemic control
Shimels [75]	2018	Cross-sectional	Ethiopia	Type 2 diabetes; ≥ 18 years	361	FPG	glycemic control (FPG: 100–130 mg/dl)		46–60 years	Odds Ratio	0.66	0.37	1.18	No significant association between age of 46–60 years with glycaemic control
									˃ 60 years		0.74	0.36	1.52	No significant association between age of more than 60 years with glycaemic control
Tefera [77]	2020	Cross-sectional	Ethiopia	Type 2 diabetes; ≥ 18 years	400	FPG	Controlled (FPG:80–130 mg/dl)		˂ 40 years	Odds Ratio	6.23	1.99	9.11	Being of age less than 40 years was significantly associated with reaching glycaemic target
									40–60 years		2.52	0.47	3.61	No significant association has been found with 40–60 years age group age
Tekalegn [78]	2018	Cross-sectional	Ethiopia	Type 2 diabetes; ≥ 15 years	422	FBG	Good control (average FBG:70–130 mg/dL); poor control (average FBG > 130 or < 70 mg/dL)		40–49 years	Odds Ratio	2.14	0.74	6.2	No significant association between gycaemic control and age group 40–49 years
									50–59 years		2.46	0.91	6.63	No significant association between glycaemia control and age group 50–59 years
									≥ 60 years		1.02	0.37	2.78	No significant association between glycaemic control and age ≥ 60 years
Yimam [86]	2020	Cross-sectional	Ethiopia	Type 2 diabetes with hypertension; ≥ 18 years	300	FBG	Good control (mean FBG:80–130 mg/dl); poor control (FBG < 80 mg/dl or > 130 mg/dl)		41–60 years	Odds Ratio	3.05	1.20	7.77	Age 41–60 years was significantly associated with poor glycaemic control
									˃ 60 years		2.62	1.01	6.80	Age more than 60 years was significantly associated with poor glycaemic control
Abera [88]	2022	Cross-sectional	Ethiopia	Type 2 diabetes	325	HbA1c	Good control (HbA1c < 7%); poor control (HbA1c ≥ 7%)		18–44 years	Odds Ratio	1.63	0.66	4.18	Significant association between age groups of 45–54 years and ≥ 65 years and poor glycaemic control
									45–54 years		2.46	1.28	6.01	
									≥ 65 years		1.97	1.3	5.97	
Abebe [89]	2022	Cross-sectional	Ethiopia	Type 2 diabetes; > 18 years	138	FBG	Good control (mean FBG:80–130 mg/dl); poor control (FBG < 80 mg/dl or > 130 mg/dl)		≥ 60 years	Odds Ratio	0.4	0.16	1.008	No significant relationship between age ≥ 60 years and glycaemic control
Adeniyi [18]	2016	Cross-sectional	South Africa	Type 2 diabetes; ≥ 30 years at diagnostic of DM	327	HbA1c	Good control (HbA1c ≤ 7%); poor control (HbA1c > 7%); moderately poor (HbA1c = 7–8.9%); critically poor (HbA1c ≥ 9%)	Gender	Female gender	Odds Ratio	3.4	1.5	7.7	Female gender was a significant determinant of uncontrolled diabetes
Demoz [34]	2021	Cross-sectional	Ethiopia	Type 2 diabetes; ≥ 18 years	357	HbA1c and FBG	Adequate control ( average fasting blood glucose 70–130 mg/dL or HbA1c < 7%); poor control (average fasting blood glucose > 130 or < 70 mg/dL or HbA1c > 7%)			Odds Ratio	1.59	1.20	2.38	Being Female was significantly associated with poor glycaemic control
Biru [29]	2017	Cross-sectional	Ethiopia	Type 2 diabetes; ≥ 18 years	322	FBG	Good control (FBG ≤ 110 mg/dL)			Odds Ratio	3.47	1.22	9.91	Female gender was significanty associated with good glycaemic control
Kimando [51]	2017	Cross-sectional	Kenya	Type 2 diabetes without overt complications; ≥ 30 years	385	HbA1c	Sub-optimal control (HbA1c > 7.0%)			Odds Ratio	1.1	0.7	1.6	No significant association between Female gender and glycaemic control
Mphwanthe [61]	2020	Cross-sectional	Malawi	Type 2 diabetes; ≥ 25 years	428	HbA1c	Poor control (HbA1c ≥ 8%)			ß coefficient	− 0.197	− 0.492	0.098	Female gender has no significant correlation with glycaemic control
Mwavua [64]	2016	Cross-sectional	Kenya	Type 2 diabetes; ≥ 18 years	200	HbA1c	Good control (HbA1c < 7%); poor control (HbA1c ≥ 7%)			Odds Ratio	1.0	0.4	2.4	Female gender had no significant correlation with glycaemic control
Mwita [65]	2019	Cross-sectional	Botswana	Type 2 diabetes; ≥ 18 years	500	HbA1c	Optimal glycaemic (HbA1c < 7%)			Odds Ratio	0.42	0.14	1.25	Female gender had no correlation with glycaemic control
Rwegerera [73]	2019	Cross-sectional	Botswana	Type 2 diabetes	368	HbA1c	desirable (< 7%); suboptimal (7–9%); poor (≥ 9%)			Odds Ratio	1.91	1.09	3.36	Female gender was significantly associated with good glycaemic control
BeLue [27]	2016	Cross-sectional	Senegal	Type 2 diabetes	106	HbA1c	Glycemic control (HbA1c < 7%)		Male gender	ß coefficient	1.5	0.86	2.14	Male gender was significantly positive association with poor glycaemic control
Shimels [75]	2018	Cross-sectional	Ethiopia	Type 2 diabetes; ≥ 18 years	361	FPG	Glycemic control (FPG: 100–130 mg/dl)			Odds Ratio	0.26	0.13	0.53	Male gender was significantly associated with good glycaemic control
Mobula [58]	2018	Cross-sectional	Ghana	hypertensive and Type 2 diabetes; ≥ 18 years	1226	HbA1c	Poor control (HBA1c ≥ 7%)			Odds Ratio	0.66	0.49	0.88	Male gender was significantly associated with good glycaemic control
Botchway [31]	2021	Cross-sectional	Ghana	Type 2 diabetes; ≥ 18 years	254	HbA1c	No threshold			ß coefficient	'− 0.30	− 0.68	− 0.08	Male gender had no significant correlation with glycaemic control
Fekadu [39]	2019	Cross-sectional	Ethiopia	Type 2 diabetes; 18–86 years	228	FBG	Good control (FBG: 70–130 mg/dL); poor control (< 70 mg/dL and > 130 mg/dL)			Odds Ratio	0.32	1.63	20.19	Male gender had no significant association with glycaemic control
Id [44]	2021	Cross-sectional	Ethiopia	Type 2 diabetes;, > 18 years	394	FBS	Good blood glucose control (< 154 mg/dl); poor blood glucose control (≥ 154 mg/dl)			Odds Ratio	1.41	0.89	2.2	Male sex had no significant association with glycaemic control
Noor [66]	2017	Cross-sectional	Sudan	Type 2 diabetes	387	HbA1c	Controlled < 7% Uncontrolled > 7%			Odds Ratio	1.250	0.491	3.179	Male sex had no significant association with glycaemic control
Tefera [77]	2020	Cross-sectional	Ethiopia	Type 2 diabetes; ≥ 18 years	400	FPG	Controlled (FPG:80–130 mg/dl)			Odds Ratio	1.71	0.87	3.37	Male gender had no association with glycaemic control
Woldu [83]	2014	Cross-sectional	Ethiopia	Type 2 diabetes	102	FBG	Poor glycaemic control (FBG level of > 126 mg/dl)			Odds Ratio	0.3	0.051	1.718	No association between male gender and glycaemic control
Yigazu [85]	2017	Cross-sectional	Ethiopia	Type 2 diabetes; 18–80 years	174	FBG	Controlled (average FBG: 80–130 mg/dL); Uncontrolled (average FBG > 130 or < 70 mg/dL)			Odds Ratio	1.58	0.79	3.15	No association between male gender and glycaemic control
Yosef [87]	2021	Cross-sectional	Ethiopia	Type 2 diabetes; ≥ 18 years	245	FBG	Good control (FBG:70–130 mg/dL)			Odds Ratio	2.28	1.24	4.21	Male gender was associated with poor glycaemic control
Adeniyi [18]	2016	Cross-sectional	South Africa	Type 2 diabetes; ≥ 30 years at diagnostic of diabetes	327	HbA1c	Good control (HbA1c ≤ 7%); poor control (HbA1c > 7%); moderately poor (HbA1c = 7–8.9%); critically poor (HbA1c ≥ 9%)	Income	Individual monthly income	Odds Ratio	2.9	1.3	6.5	Lower monthly income was associated with poor glycaemic control
Ayele [25]	2019	Cross-sectional	Ethiopia	Type 2 diabetes; > 18 years	275	FBG	Good control (FBG level between 70 and 130 mg/dL); poor control (FBG greater than 130 or less than 70 mg/ dL)			Odds Ratio	1.724	0.719	4.131	No association found between monthly income in ETB ≥ 2500 and glycaemic control
											0.755	0.271	2.102	No association found between monthly income in ETB 1500–2500 and glycaemic control
Yosef [87]	2021	Cross-sectional	Ethiopia	Type 2 diabetes; ≥ 18 years	245	FBG	Good control (FBG:70–130 mg/dL)			Odds Ratio	2.14	1.17	3.91	Significant association between a monthly income of less than 136 american dollars and poor glycaemic control
Mphwanthe [61]	2020	Cross-sectional	Malawi	Type 2 diabetes; ≥ 25 years	428	HbA1c	Poor control (HbA1c ≥ 8%)		Household Income	ß coefficient	0.087	− 0.157	0.331	No association found between household income level (≥ 30,000.34 MWK) and glycaemic control
BeLue [27]	2016	Cross-sectional	Senegal	Type 2 diabetes	106	HbA1c	Glycemic control (HbA1c < 7%)	Marital status	Unmarried	ß coefficient	− 0.108	− 0.778	0.562	Being unmarried had no significant correlation with glycaemic control
Demoz [34]	2019	Cross-sectional	Ethiopia	Type 2 diabetes; ≥ 18 years	357	HbA1c and FBG	Adequate control ( average fasting blood glucose 70–130 mg/dL or HbA1c < 7%); poor control (average fasting blood glucose > 130 or < 70 mg/dL or HbA1c > 7%)			Odds Ratio	0.93	0.81	1.35	No significant association between glycaemic control and being never married
Ayele [25]	2019	Case–control	Ethiopia	Type 2 diabetes with poor glycemic control (cases) and without good glycemic control (controls); > 18 years	410	FBG	Good control (average fasting blood glucose of 80–130 mg/dL); poor control (average fasting blood glucose of > 130 mg/dL)		Single/ divorced /widowed	Odds Ratio	1.80	0.68	4.72	Being single/divorced/widowed had no significant relationship with glycaemic control
Mashele [56]	2019	Cross-sectional	South Africa	Type 2 diabetes; 35–74 years	176	HbA1c	Optimal control (HbA1c < 7%); poor control (HbA1c ≥ 7%)		Marital status	Odds Ratio	1.006	0.962	1.051	Marital status had no significant relationship with glycaemic control
Mphwanthe [61]	2020	Cross-sectional	Malawi	Type 2 diabetes; ≥ 25 years	428	HbA1c	Poor control (HbA1c ≥ 8%)		Married	ß coefficient	− 0.119	− 0.427	0.189	Being married had no significant correlation with glycaemic control
Kimando [51]	2017	Cross-sectional	Kenya	Type 2 diabetes without overt complications; ≥ 30 years	385	HbA1c	Sub-optimal control (HbA1c > 7.0%)		Separated	Odds Ratio	1.4	0.1	15.3	No significant relationship between glycaemic control and being separated
									Unmarried		1.8	0.7	4.8	No significant relationship between glycaemic control and being single or unmarried
									Widowed		1.1	0.7	1.7	No significant relationship between glycaemic control and being a widowed
Rwegerera [73]	2019	Cross-sectional	Botswana	Type 2 diabetes	368	HbA1c	desirable (< 7%); suboptimal (7–9%); poor (≥ 9%)		Single/ Separated /Widowed	Odds Ratio	1.41	0.69	2.89	Being single/separated/widowed had no significant relationship with glycaemic control
Ayele [25]	2019	Cross-sectional	Ethiopia	Type 2 diabetes; > 18 years	275	FBG	Good control (FBG level between 70 and 130 mg/dL); Poor control (FBG greater than 130 or less than 70 mg/ dL)	Place of residence	Rural	Odds Ratio	1.403	0.442	4.454	No significant association found between rural residency and glycaemic control
Ayele [25]	2019	Case–control	Ethiopia	Type 2 diabetes with poor glycemic control (cases) and without good glycemic control (controls); > 18 years	410	FBG	Good control (average fasting blood glucose of 80–130 mg/dL); poor control (average fasting blood glucose of > 130 mg/dL)			Odds Ratio	0.66	0.24	1.85	Rural residency had no significant association with glycaemic control
Woldu [83]	2014	Cross-sectional	Ethiopia	Type 2 diabetes	102	FBG	Poor control (FBG level of > 126 mg/dl)			Odds Ratio	0.5	0.106	1.986	Residing in rural area had no significant association with glycaemic control
Kefale [49]	2019	Cross-sectional	Ethiopia	Type 2 diabetes; ≥ 18 years	169	FBS	Glycemic control (FBS ≤ 130 mg/dl in all these most three recent measurements)		Urban	Odds Ratio	2.5	1.1	5.7	Urban residency was significantly associated with poor glycaemic control
Tefera [77]	2020	Cross-sectional	Ethiopia	Type 2 diabetes; ≥ 18 years	400	FPG	Controlled (FPG:80–130 mg/dl)			Odds Ratio	0.72	0.27	1.93	Residing in urban area was not significantly associated with glycaemic control
Mobula [58]	2018	Cross-sectional	Ghana	hypertensive and Type 2 diabetes; ≥ 18 years	1226	HbA1c	Poor control (HBA1c ≥ 7%)		Urban	Odds Ratio	1.09	0.74	1.60	Residing in urban area had no significant relationship with glycaemic control
									Semi-urban		0.88	0.57	1.38	Residing in semi-urban area had no significant relationship with glycaemic control
Mphwanthe [61]	2020	Cross-sectional	Malawi	Type 2 diabetes; ≥ 25 years	428	HbA1c	Poor control (HbA1c ≥ 8%)		Semi-urban	ß coefficient	− 0.172	− 0.446	0.102	Residing in semi-urban area had no significant relationship with glycaemic control
Ayele [25]	2019	Cross-sectional	Ethiopia	Type 2 diabetes; > 18 years	275	FBG	Good control (FBG level between 70 and 130 mg/dL); Poor control (FBG greater than 130 or less than 70 mg/ dL)	Employment	Farmer	Odds Ratio	0.279	0.098	0.797	Being farmer was inversely associated with good glycaemic control
									Unemployed		1.268	0.354	4.549	No significant association was found between glycaemic control with unemployed
									NGO employed		4.059	0.775	21.253	No significant association was found between glycaemic control with NGO employed
									Merchant		0.322	0.102	1.107	No significant association was found between glycaemic ccontrol with merchant
BeLue [25]	2016	Cross-sectional	Senegal	Type 2 diabetes	106	HbA1c	Glycemic control (HbA1c < 7%)		Employed	ß coefficient	0.07	0.01	0.13	No significant association found between being employed and glycaemic control
Kassahun [48]	2016	Cross-sectional	Ethiopia	Type 2 diabetes; ≥ 18 years	309	FBG	Poor control (mean FBG > 130 mg/dl)		Farmer	Odds Ratio	2.47	1.13	5.39	Being farmer has been found significantly associated with poor glycaemic control
									Employed		2.65	0.96	7.24	Significant association between poor glycaemic control with employed
									Merchant		2.69	0.86	8.37	No significant association between glycaemic control with merchant
									Daily labor		2.22	0.80	6.11	No significant association between glycaemic control with daily labor
Kimando [51]	2017	Cross-sectional	Kenya	Type 2 diabetes without overt complications; ≥ 30 years	385	HbA1c	Sub-optimal control (HbA1c > 7.0%)		Employed	Odds Ratio	0.9	0.4	2.3	No significant association between glycaemic control and being employed
									Self-employed		0.9	0.5	1.4	No significant association between glycaemic control and being self-employed
									Retired		0.9	0.5	1.6	No significant association between glycaemic control and being retired
BeLue [27]	2016	Cross-sectional	Senegal	Type 2 diabetes	106	HbA1c	Glycemic control (HbA1c < 7%)	Education level	No formal education	ß coefficient	0.11	− 0.44	0.66	Being of no education class was not significantly associated with glycaemic control
Biru [29]	2017	Cross-sectional	Ethiopia	Type 2 diabetes; ≥ 18 years	322	FBG	Good control (FBG ≤ 110 mg/dL)		Primary level	Odds Ratio	13.66	2.94	63.55	Being of primary level of education was significantly associated with good glycaemic control
									Secondary level		20.09	3.80	106.14	Being of secondary level of education was significantly associated with good glycaemic control
									Higher education		20.72	3.78	113.51	Being of higher education was associated with good glycaemic level
Botchway [31]	2021	Cross-sectional	Ghana	Type 2 diabetes; ≥ 18 years	254	HbA1c	No threshold		Junior secondary level	ß coefficient	− 0.21	− 0.62	0.20	No significant association between glycaemic control and having completed junior secondary school
									Senior secondary level or higher education		0.33	− 0.14	0.80	No significant association between glycaemic control and having completed senior secondary school or higher education
Demoz [34]	2019	Cross-sectional	Ethiopia	Type 2 diabetes; ≥ 18 years	357	HbA1c and FBG	Adequate control (average fasting blood glucose 70–130 mg/dL or HbA1c < 7%); poor control (average fasting blood glucose > 130 or < 70 mg/dL or HbA1c > 7%)		No formal education	Odds Ratio	1.59	0.37	1.09	No significant association between glycaemic control and no formal education
									Primary level		2.10	0.75	1.77	No significant association between glycaemic control and having primary level of education
									Secondary level		1.11	0.55	1.31	No significant association between glycaemic control with secondary level of education
Fekadu [39]	2019	Cross-sectional	Ethiopia	Type 2 diabetes; 18–86 years	228	FBG	Good control (FBG: 70–130 mg/dL); poor control (< 70 mg/dL and > 130 mg/dL)		Unable to read and write/illiterate	Odds Ratio	3.12	1.52	8.50	Being unable to read and write/illiterate was significantly associated with poor glycaemic control
									No formal education		2.28	2.14	32.60	Having informal education was significantly associated with poor glycaemic control
									Primary level		1.03	0.64	2.14	Being of primary education was significantly associated with poor glycaemic control
									Secondary level		1.04	0.26	17.48	Being of secondary level of education was not significantly associated with poor glycaemic control
Fseha [40]	2017	Cross-sectional	Ethiopia	Type 2 diabetes; 22–60 years	200	FBS	Good (FBS 70–130 mg/dl), poor (FBS > 130 mg/dl)		Formal education	Odds Ratio	1.054	0.492	2.261	Having had formal education was not significantly associated with glycaemic control
Kassahun [48]	2016	Cross-sectional	Ethiopia	Type 2 diabetes; ≥ 18 years	309	FBG	Poor control (mean FBG > 130 mg/dl)		Illiterate	Odds Ratio	3.45	1.01	11.91	Being illiterate has been found significantly associated with poor glycaemic control
									Able to read and write		0.81	0.20	3.26	No significant association between glycaemic control and being able to read and write
									Primary level		2.45	0.85	7.03	No significant association between glycaemic control and having reached 1–8 years of education
									Secondary level		1.97	0.69	5.55	No significant association between glycaemic control and having reachd 9–12 years of eductaion
Kimando [51]	2017	Cross-sectional	Kenya	Type 2 diabetes without overt complications; ≥ 30 years	385	HbA1c	Sub-optimal control (HbA1c > 7.0%)		No education	Odds Ratio	1.0	0.5	1.9	No significant association between glycaemic control and being illiterate
									Secondary level		1.0	0.5	2.0	No significant association between glycaemic control and having a secondary level of education
									Tertiairy level		0.9	0.3	2.8	No significant association between glycaemic control and being of tertiary level
Mashele [56]	2019	Cross-sectional	South Africa	Type 2 diabetes; 35–74 years	176	HbA1c	Optimal control (HbA1c < 7%); poor control (HbA1c ≥ 7%)		Level of education	Odds Ratio	1.014	0.985	1.045	The level of education had no significant relationship with glycaemic control
Mphwanthe [61]	2020	Cross-sectional	Malawi	Type 2 diabetes; ≥ 25 years	428	HbA1c	Poor control (HbA1c ≥ 8%)		Secondary level and above	ß coefficient	− 0.300	− 0.576	− 0.024	Being of secondary and above level of education had no significant association with glycaemic control
Mwavua [64]	2016	Cross-sectional	Kenya	Type 2 diabetes; ≥ 18 years	200	HbA1c	Good control (HbA1c < 7%); poor control (HbA1c ≥ 7%)		primary level and above	Odds Ratio	0.8	0.3	1.8	Having highest level of education below primary had no significant relationship with glycaemic control
Omar [65]	2018	Cross-sectional	Sudan	Type 2 diabetes; ≥ 18 years	339	HbA1c	Good control (HbA1c < 7%); poor control (HbA1c ≥ 7%)		Less or equal to secondary level	Odds Ratio	1.35	0.76	2.242	Being of an education less or equal to secondary level had no significant association with glycaemic control
Rwegerera [73]	2019	Cross-sectional	Botswana	Type 2 diabetes	368	HbA1c	desirable (< 7%); suboptimal (7–9%); poor (≥ 9%)		Primary to secondary completed	Odds Ratio	1.44	0.44	4.74	No singificant relationship between having completed primary to secondary school and glycaemic control
									College/University/Postgraduate completed		1.14	0.42	3.1	No singificant relationship between having completed College/University/Postgraduate and glycaemic control
Tefera [77]	2020	Cross-sectional	Ethiopia	Type 2 diabetes; ≥ 18 years	400	FPG	Controlled (FPG:80–130 mg/dl)		Elementary school	Odds Ratio	0.20	0.02	1.65	No significant association between glycaemic control and having reached elementary school
									High school		0.20	0.02	1.63	No significant association between glycaemic control and having reached high school
									Higher education		0.45	0.05	3.86	No significant association between glycaemic control and having reached higher institution
									Able to read and write		0.12	0.01	1.05	No significant association between glycaemic control and being able to read and write
Yosef [87]	2021	Cross-sectional	Ethiopia	Type 2 diabetes; ≥ 18 years	245	FBG	Good control (FBG:70–130 mg/dL)		No formal education		3.12	1.53	6.35	Significant association between no formal education and poor glycaemic control
BeLue [27]	2016	Cross-sectional	Senegal	Type 2 diabetes	106	HbA1c	Glycemic control (HbA1c < 7%)	Unsatisfied household situation	Unsatisfied household situation	ß coefficient	− 0.701	-1.266	− 0.146	Having Unsatisfied household situation had no significant association with glycaemic control
Kamuhabwa [47]	2014	Cross-sectional	Tanzania	Type 2 diabetes; ≥ 18 years	469	FBG	Good control (FBG ≤ 130 mg/dL or 7.2 mmol/L); poor control (FBG > 130 mg/dL or 7.2 mmol/L)	Health insurance	Absence of health insurance	Odds Ratio	1.861	1.044	3.318	Absence of health insurance was significantly associated with poor glycaemic control
Mobula [58]	2018	Cross-sectional	Ghana	Hypertensive and Type 2 diabetes; ≥ 18 years	1226	HbA1c	Poor control (HBA1c ≥ 7%)			Odds Ratio	1.41	1.09	1.82	The absence of health insurance was significantly associated with poor glycaemic control
Ayele [25]	2019	Cross-sectional	Ethiopia	Type 2 diabetes; > 18 years	275	FBG	Good control (FBG level between 70 and 130 mg/dL); poor control (FBG greater than 130 or less than 70 mg/ dL)	Distance from home to the health structure	Distance less than 100 km	Odds Ratio	13.195	3.193	54.517	Residing from less than 100 km from the hospital was significantly associated with glycaemic control
Mphwanthe [61]	2020	Cross-sectional	Malawi	Type 2 diabetes; ≥ 25 years	428	HbA1c	Poor control (HbA1c ≥ 8%)		Distance ≥ 5 kilometers	ß coefficient	− 0.167	− 0.428	0.094	Residing at a distance to the hospital ≥ 5 km was not significantly associated with glycaemic control
Fseha [40]	2017	Cross-sectional	Ethiopia	Type 2 diabetes; 22–60 years	200	FBS	Good (FBS 70–130 mg/dl), poor (FBS > 130 mg/dl)	Wealth status	Medium	Odds Ratio	2.335	0.933	5.844	Medium wealth status had no significant association with glycaemic control
									Rich		0.679	0.313	1.471	Rich Wealth status had no significant association with glycaemic control
Camara [32]	2014	Cross-sectional	Cameroon and Guinea	Type 2 diabetes mellitus; ≥ 16 years	1267	HbA1c	Good control (HbA1C < 7.0% or < 53 mmol/mol)	Country of residence	Guinea	Odds Ratio	2.62	1.90	3.61	Residing in Guinea was significantly associated with poor glycaemic control
Osuji [68]	2018	Cross-sectional	Nigeria	Type 2 diabetes; ≥ 18 years	316	HbA1c	Good (HbA1c < 7%); poor (HbA1c ≥ 7%)	Family support	Perceived family support	Odds Ratio	112.51	46.638	271.440	Perceived family support was significantly associated with good glycaemic control
Botchway [31]	2021	Cross-sectional	Ghana	Type 2 diabetes; ≥ 18 years	254	HbA1c	No threshold	Religion	Frequency participating in religious activities	ß coefficient	− 0.22	− 0.32	− 0.12	Frequency of participating in religious activities had a significant association with good glycaemic control
Botchway [31]	2021	Cross-sectional	Ghana	Type 2 diabetes; ≥ 18 years	254	HbA1c	No threshold	Social support	Social support	ß coefficient	0.25	0.08	0.42	Social support had no significany association with glycaemic control
Botchway [31]	2021	Cross-sectional	Ghana	Type 2 diabetes; ≥ 18 years	254	HbA1c	No threshold	Alternative care	Frequency seeking traditional medicine practitioners	ß coefficient	1.40	0.92	1.88	The frequency of seeking Traditional Medicine practitioners had a significant association with poor glycaemic control

*HbA1c* Haemoglobin A1c, *FBG* Fasting blood glucose, *FBS* Fasting blood sugar, *FPG* Fasting plasma glucose, *MWK* Malawian Kwanza Rates (1 MWK = 0.00122305 USD), *ETB* Ethiopian Birr Rates (1 ETB = 0.0211419 USD)

**Table 3:** Lifestyle factors and glycaemic control in sub-Saharan Africa

First author name	Year	Study design	Study setting	Study population	Sample size	Measurement of glycemic control	Definition of glycaemic control	Generic factor	Specific factors	Measure of association	Point estimate	Lower bound	Upper bound	Association with glycemic control
Abera [88]	2022	Cross-sectional	Ethiopia	Type 2 diabetes	325	HbA1c	Good control (HbA1c < 7%); poor control (HbA1c ≥ 7%)	Dietary adherence	Poor dietary adherence	Odds Ratio	1.97	1.28	3.52	Poor dietary adherence was significantly associated with poor glycaemic control
Achila [17]	2020	Cross-sectional	Eritrea	Type 2 diabetes; 20–88 years	309	HbA1c	Poor control (HbA1c ≥ 7%)		Good dietary adherence	Odds Ratio	2.4	0.84	6.86	Following diet as prescribed is not significabtly associated with glycaemic control
Biru [29]	2017	Cross-sectional	Ethiopia	Type 2 diabetes; ≥ 18 years	322	FBG	Good control (FBG ≤ 110 mg/dL)			Odds Ratio	3.27	1.23	8.67	Good dietary adherence was found significantly associated with good glycaemic control
Eticha [36]	2016	Cross-sectional	Ethiopia	Type 2 diabetes; ≥ 18 years	384	HbA1c	Good control (HbA1c < 7%); poor control (HbA1c ≥ 7%)			Odds Ratio	0.3	0.1	0.5	Following recommended diet was significabtly associated with good glycaemic control
Fseha [40]	2017	Cross-sectional	Ethiopia	Type 2 diabetes; 22–60 years	200	FBS	Good (FBS 70–130 mg/dl), Poor (FBS > 130 mg/dl)			Odds Ratio	2.529	1.267	5.046	Taking meal appropriately was significantly associated with good glycaemic control
Mohammed [60]	2020	Cross-sectional	Ethiopia	Type 2 diabetes	307	FBG	Good control (average of last three FBG results between 70 mg/dL and 130 mg/dL)			Odds Ratio	3.56	1.75	8.23	Dietary adherence was found significantly associated with good glycemic control
Shimels [75]	2018	Cross-sectional	Ethiopia	Type 2 diabetes; ≥ 18 years	361	FPG	glycemic control (FPG: 100–130 mg/dl)			Odds Ratio	1.63	0.96	2.75	Good dietary adherence was found not significantly associated with glycaemic control
Demoz [34]	2019	Cross-sectional	Ethiopia	Type 2 diabetes; ≥ 18 years	357	HbA1c and FBG	Adequate control (average fasting blood glucose 70–130 mg/dL or HbA1c < 7%); Poor control (average fasting blood glucose > 130 or < 70 mg/dL or HbA1c > 7%)		Poor dietary adherence	Odds Ratio	3.44	0.71	1.55	Poor adherence to dietary plan was not significantly associated with glycaemic control
Doglikuu [35]	2021	Cross-sectional	Ghana	Type 2 diabetes; ≥ 18 years	530	HbA1C	Low, moderate, and high		Low adherence to diabetics’ feeding recommendation	Odds Ratio	2.56	1.44	4.56	Low adherence to diabetics’ feeding recommendation was associated significantly with poor glycaemic control
									Low adherence to fruit and vegetables		2.71	1.48	4.99	Low adherence to fruit and vegetables was associated significantly with poor glycaemic control
									Low adherence to whole grain, beans, starchyfruits and plantain		3.29	1.81	6.02	Low adherence to whole grain, beans, starchyfruits and plantain was associated significantly with poor glycaemic control
									Low adherence to foods prepared with walnut, canola, sunflower, cotton seed and fish oils		2.62	1.49	4.58	Low adherence to foods prepared with walnut, canola, sunflower, cotton seed and fish oils was associated significantly with poor glycaemic control
Fekadu [39]	2019	Cross-sectional	Ethiopia	Type 2 diabetes; 18–86 years	228	FBG	Good control (FBG: 70–130 mg/dL); poor control (< 70 mg/dL and > 130 mg/dL)		Inadequate dietary adherence	Odds Ratio	1.82	0.31	2.15	Following an healthful eating plan for 0–3 days is not significantly associated with glycaemic control
Kefale [49]	2019	Cross-sectional	Ethiopia	Type 2 diabetes; ≥ 18 years	169	FBS	Glycemic control (FBS ≤ 130 mg/dl)		No adherence to diet	Odds Ratio	0.8	1.267	5.046	No adherence to diet was found not significantly associated with glycaemic control
Mphwanthe [62]	2020	Cross- sectional	Malawi	Type 2 diabetes	428	HbA1c	Poor control (HbA1c ≥ 8%)		Number of meals	Odds Ratio	2.680	1.145	4.970	Number of meals was significantly associated with good glycaemic control
									Carbohydrates (CHO) percentage per day		1.167	1.107	1.231	CHO % of energy/day was found significantly associated with good glycaemic control
									Preventive diet score		1.015	0.730	1.412	Preventive diet score was not associated with glycaemic control
									Fat pourcentage of energy per day		1.063	0.968	1.168	Fat % of energy/day was not associated with glycaemic control
									Polyunsaturated fat (PUFA) (g/day)		1.113	0.828	1.496	PUFA (g/day) was not associated with glycaemic control
									Amount of fruit and vegetables (g/day)		0.432	0.165	1.132	Amount of fruit and vegetables (g/day) was not associated with glycaemic control
									Followed diet recommendation		0.996	0.532	1.865	Followed diet recommendation was found not significantly associated with glycaemic control
Omar [67]	2018	Cross-sectional	Sudan	Type 2 diabetes; ≥ 18 years	339	HbA1c	Good control (HbA1c < 7%); Poor control (HbA1c ≥ 7%)		Adding sugar to food	Odds Ratio	1.73	1.07	2.80	Adding sugar to food was found significantly associated with poor glycemic control
Abera [88]	2022	Cross-sectional	Ethiopia	Type 2 diabetes	325	HbA1c	Good control (HbA1c < 7%); Poor control (HbA1c ≥ 7%)		diet adherence of 0–3 days per week	Odds Ratio	1.97	1.28	3.52	Diet adherence of 0–3 days per week is associated with poor glycaemic control
Adeniyi [18]	2016	Cross-sectional	South Africa	Type 2 diabetes; ≥ 30 years at diagnostic of diabetes	327	HbA1c	Good control (HbA1c ≤ 7%); poor control (HbA1c > 7%); moderately poor (HbA1c = 7–8.9%); critically poor (HbA1c ≥ 9%)	Physical activity	Sedentary habits	Odds Ratio	21	7.2	61.3	Sedentary habits was found significantly associated with poor glycaemic control
Biru [29]	2017	Cross-sectional	Ethiopia	Type 2 diabetes; ≥ 18 years	322	FBG	Good control (FBG ≤ 110 mg/dL)		Practice of exercise	Odds Ratio	3.37	1.39	8.20	Adherence to exercice was associated with good glycaemic control
Demoz [34]	2019	Cross-sectional	Ethiopia	Type 2 diabetes; ≥ 18 years	357	HbA1c and FBG	Adequate control ( average fasting blood glucose 70–130 mg/dL or HbA1c < 7%); Poor control (average fasting blood glucose > 130 or < 70 mg/dL or HbA1c > 7%)			Odds Ratio	2.92	0.78	1.10	Exercising was found not significantly associated with glycaemic control
Eticha [36]	2016	Cross-sectional	Ethiopia	Type 2 diabetes; ≥ 18 years	384	HbA1c	Good control (HbA1c < 7%); poor control (HbA1c ≥ 7%)			Odds Ratio	0.1	0.1	0.2	Participating in physical exercise was found to be significantly associated with good glycaemic control
Shimels [75]	2018	Cross-sectional	Ethiopia	Type 2 diabetes; ≥ 18 years	361	FPG	Glycemic control (FPG: 100–130 mg/dl)			Odds Ratio	1.00	0.47	2.13	Being active was found not significantly associated with glycaemic control
Kefale [49]	2019	Cross-sectional	Ethiopia	Type 2 diabetes; ≥ 18 years	169	FBS	Glycemic control (FBS ≤ 130 mg/dl in all these most three recent measurements)			Odds Ratio	1.2	0.5	2.6	Exercising regularly was not significantly associated with glycaemic control
Fekadu [39]	2019	Cross-sectional	Ethiopia	Type 2 diabetes; 18–86 years	228	FBG	Good control (FBG: 70–130 mg/dL); poor control (< 70 mg/dL and > 130 mg/dL)		Inadequate physical activity	Odds Ratio	3.19	1.05	19.84	Doing exercise planned 0–3 days was significantly associated with poor glycaemic control (p:0.019)
Mamo [53]	2019	Case–control	Ethiopia	Type 2 diabetes with poor glycemic control (cases) and without good glycemic control (controls); > 18 years	410	FBG	Good control (average fasting blood glucose of 80–130 mg/dL); poor control (average fasting blood glucose of > 130 mg/dL)			Odds Ratio	4.79	1.70	13.53	Inadequate physical activity was significantly associated with poor glycaemic control
Fseha [40]	2017	Cross-sectional	Ethiopia	Type 2 diabetes; 22–60 years	200	FBS	Good (FBS 70–130 mg/dl), Poor (FBS > 130 mg/dl)		Moderate physical activity	Odds Ratio	2.927	1.335	6.420	Moderate physical exercise was found to be significantly associated with good glycaemic control
Mphwanthe [62]	2020	Cross- sectional	Malawi	Type 2 diabetes; ≥ 25 years	428	HbA1c	Poor control (HbA1c ≥ 8%)		Physical activity level	ß coefficient	− 0.143	− 0.186	− 0.100	Physical activity level was significantly correlated with good glycaemic control (p:0.001)
BeLue [128]	2016	Cross-sectional	Senegal	Type 2 diabetes	106	HbA1c	Glycemic control (HbA1c < 7%)	Smoking	Not smoking	ß coefficient	− 0.25	-1.25	0.75	Being a no smoker was not found significantly associated with glycaemic control
Fekadu [39]	2019	Cross-sectional	Ethiopia	Type 2 diabetes; 18–86 years	228	FBG	Good control (FBG: 70–130 mg/dL); poor control (< 70 mg/dL and > 130 mg/dL)		Smoking	Odds Ratio	4.51	0.00	0.50	Smoking was found significantly associated with poor glycaemic control (p:0.022)
Woldu [83]	2014	Cross-sectional	Ethiopia	Type 2 diabetes	102	FBG	Poor control (FBG level of > 126 mg/dl)			Odds Ratio	2.7	0.264	27.102	Smoking was not significantly associated with glycaemic control
Fekadu [39]	2019	Cross-sectional	Ethiopia	Type 2 diabetes; 18–86 years	228	FBG	Good control (FBG: 70–130 mg/dL); poor control (< 70 mg/dL and > 130 mg/dL)	Alcohol consumption	Acohol consumption	Odds Ratio	1.44	1.24	19.02	Alcohol consumption was not significantly associated with glycaemic control (p:0.177)
Biru [29]	2017	Cross-sectional	Ethiopia	Type 2 diabetes; ≥ 18 years	322	FBG	Good control (FBG ≤ 110 mg/dL)		History of alcohol consumption	Odds Ratio	0.15	0.03	0.65	The fact to ever had drunk alcohol was found significantly associated with poor glycaemic control
Kefale [49]	2019	Cross-sectional	Ethiopia	Type 2 diabetes; ≥ 18 years	169	FBS	Glycemic control (FBS ≤ 130 mg/dl)	Use of illicit substance	Use of illicit substance	Odds Ratio	0.5	0.2	1.2	Previous use of substance was not significantly associated with glycaemic control

*HbA1c* Haemoglobin A1c, *FBG* Fasting blood glucose, *FBS* Fasting blood sugar, *FPG* Fasting plasma glucose

**Table 4:** Clinical factors and glycaemic control in sub-Saharan Africa

First author name	Year	Study design	Study setting	Study population	Sample size	Measurement of glycemic control	Definition of glycaemic control	Generic factor	Specific factors	Measure of association	Point estimate	Lower bound	Upper bound	Association with glycemic control
Thuita [80]	2019	Cross- sectional	Kenya	Type 2 diabetes; 20–79 years	153	HbA1c	Good control (HbA1c < 7%); poor control (HbA1c > 7%)	Past history of diabetes	Existence of family history of diabetes	β coefficient	0.119	0.066	0.874	A family history of diabetes was significantly associated with poor glycaemic control (p:0.017)
Adeniyi [18]	2016	Cross-sectional	South Africa	Type 2 diabetes; ≥ 30 years at diagnostic of DM	327	HbA1c	Good control (HbA1c ≤ 7%); poor control (HbA1c > 7%); moderately poor (HbA1c = 7–8.9%); critically poor (HbA1c ≥ 9%)	Duration of diabetes	Longer duration	Odds Ratio	35.8	4.4	294.2	Longer duration of type 2 diabetes was significantly associated with poor glycaemic control
BeLue [27]	2016	Cross-sectional	Senegal	Type 2 diabetes	106	HbA1c	Glycemic control (HbA1c < 7%)			ß coefficient	0.14	0.09	0.19	Longer duration of diabetes was significantly associated with poor glycaemic control (p < 0.05)
Botchway [31]	2021	Cross-sectional	Ghana	Type 2 diabetes; ≥ 18 years	254	HbA1c	No threshold			ß coefficient	0.04	0.02	0.06	The duration of diabetes was not significantly associated with glycaemic control
Mashele [56]	2019	Cross-sectional	South Africa	Type 2 diabetes; 35–74 years	176	HbA1c	Optimal control (HbA1c < 7%); poor control (HbA1c ≥ 7%)			Odds Ratio	1.011	1.37	2.624	Duration of diabetes was not significantly associated with glycaemic control (p:0.497)
Mobula [58]	2018	Cross- sectional	Ghana	hypertensive and Type 2 diabetes; ≥ 18 years	1226	HbA1c	Poor control (HBA1c ≥ 7%)			Odds Ratio	1.04	1.02	1.06	Duration of diabetes was significantly associated with poor glycaemic control (p:0.0005)
Mphwanthe [61]	2020	Cross- sectional	Malawi	Type 2 diabetes; ≥ 25 years	428	HbA1c	HbA1c clinically elevated ≥ 8%			ß coefficient	0.091	0.061	0.121	Duration of diabetes (p: .003) was significantly associated with poor glycaemic control (p: .003)
Omar [67]	2018	Cross-sectional	Sudan	Type 2 diabetes; ≥ 18 years	339	HbA1c	Good control (HbA1c < 7%); Poor control (HbA1c ≥ 7%)			Odds Ratio	1.04	0.99	1.10	Duration of diabetes, years was not significantly associated with glycaemic control
Mwita [65]	2019	Cross-sectional	Botswana	Type 2 diabetes; ≥ 18 years	500	HbA1c	Optimal glycaemic (HbA1c < 7%)			Odds Ratio	0.98	0.92	1.03	Diabetes duration,years was not significantly associated with glycaemic control
Belay [26]	2017	Cross-sectional	Ethiopia	Type 2 diabetes; 18–80 years	188	FPG	Good:FPG < 130 mg/dL; poor: FPG ≥ 130 mg/dL		Duration 5–10 years	Odds Ratio	2.6	1.12	6.01	Duration of diabetes 5–10 yrs was found significantly associated with poor glycaemic control
									Duration ˃10 years		3.4	1.3	9.0	Duration of diabetes > 10 yrs was found significantly associated with poor glycaemic control
Camara [32]	2014	Cross- sectional	Cameroon and Guinea	Type 2 diabetes mellitus; ≥ 16 years	1267	HbA1c	Good control (HbA1C < 7.0% or < 53 mmol/mol)		Duration ≥ 3 years	Odds Ratio	2.36	1.74	3.20	Duration of diabetes of more or equal to 3 yrs was significantly associated with poor glycaemic control
Eticha [36]	2016	Cross- sectional	Ethiopia	Type 2 diabetes; ≥ 18 years	384	HbA1c	Good control (HbA1c < 7%); poor control (HbA1c ≥ 7%)		Duration ≥ 7 years	Odds Ratio	0.8	0.4	1.6	Duration of diabetes of more or equal to 7 yrs was not significantly associated with glycaemic control
Fseha [40]	2017	Cross-sectional	Ethiopia	Type 2 diabetes; 22–60 years	200	FBS	Good (FBS 70–130 mg/dl), Poor (FBS > 130 mg/dl)			Odds Ratio	0.460	0.216	1.987	Duration of diabetes of more or equal to 7 yrs was not significantly associated with glycaemic control
Kefale [49]	2019	Case–control	Ethiopia	Type 2 diabetes; ≥ 18 years	169	FBS	Glycemic control (FBS ≤ 130 mg/dl)		Duration ≥ 5 years	Odds Ratio	1.9	0.7	5.5	Diabetes duration of more or equal to 5 years was not significantly associated with glycaemic control
Kimando [51]	2017	Cross- sectional	Kenya	Type 2 diabetes without overt complications; ≥ 30 years	385	HbA1c	Sub-optimal control (HbA1c > 7.0%)		Duration ˃ 5 years	Odds Ratio	1.1	0.7	1.6	Duration of diabetes of more than 5 years was not significantly associated with glycaemic control
Mamo [53]	2019	Case–control	Ethiopia	Type 2 diabetes with poor glycemic control (cases) and without good glycemic control (controls); > 18 years	410	FBG	Good control (average fasting blood glucose of 80–130 mg/dL); poor control (average fasting blood glucose of > 130 mg/dL)		Duration ˃ 7 years	Odds Ratio	3.08	1.33	7.16	Duration of diabetes of more than 7 years was not significantly associated with glycaemic control
Mwavua [64]	2016	Cross-sectional	Kenya	Type 2 diabetes; ≥ 18 years	200	HbA1c	Good control (HbA1c < 7%); poor control (HbA1c ≥ 7%)		Duration ≤ 10 years	Odds Ratio	0.5	0.2	1.3	Diabetes duration less or equal to 10 years was not significantly associated with glycaemic control
Noor [66]	2017	Cross-sectional	Sudan	Type 2 diabetes	387	HbA1c	Controlled < 7% Uncontrolled > 7%		Duration ≥ 5 years	Odds Ratio	0.505	0.223	1.145	Diabetes duration of 5 years or more was not significantly associated with glycaemic control
Otieno [69]	2017	Cross-sectional	Kenya	Type 2 diabetes; ≥ 30 years	220	HbA1c	Good control (HbA1c ≤ 7%); poor/suboptimal (HbA1c > 7%)		Duration 5–10 years	Odds Ratio	2.5	0.4	14.4	In patients with comorbid depression, duration of diabetes 5–10 years was not significantly associated with glycemic control
									Duration ˃ 10 years		1.3	0.3	5.3	In patients with comorbid depression, duration of diabetes > 10 years was not significantly associated with glycemic control
									Duration 5–10 years		1.0	0.4	2.1	In patients without depression, duration of diabetes 5–10 years was not significantly associated with glycemic control
									Duration ˃ 10 years		1.9	0.8	4.6	In patients without depression, duration of diabetes > 10 years was not significantly associated with glycemic control
Rwegerera [73]	2019	Cross-sectional	Botswana	Type 2 diabetes	368	HbA1c	desirable (< 7%); suboptimal (7–9%); poor (≥ 9%)		5–10 years	Odds Ratio	0.34	0.13	0.86	Having a duration of diabetes of 5–10 yrs was associated with poor glycaemic control
									˃10 years		0.42	0.15	1.15	Having a duration of diabetes of more than 10 yrs was not associated with glycaemic control
Tefera [77]	2020	Cross-sectional	Ethiopia	Type 2 diabetes; ≥ 18 years	400	FPG	Controlled (FPG:80–130 mg/dl)		Duration 5–10 years	Odds Ratio	1.95	0.87	4.40	Duration of diabetes 5–10 years was found not significantly associated with glycaemic control
									Duration ˃ 10 years		2.86	0.99	8.23	Duration of diabetes > 10 years was found not significantly associated with glycaemic control
Tekalegn [78]	2018	Cross-sectional	Ethiopia	Type 2 diabetes; ≥ 15 years	422	FBG	Good control (average FBG:70–130 mg/dL); poor control (average FBG > 130 or < 70 mg/dL)		Duration 5–10 years	Odds Ratio	2.72	1.16	6.32	Duration of diabetes from 5 to 10 years was significantly associated with poor glycaemic control
									Duration ˃ 10 years		1.7	0.8	3.7	Duration of diabetes > 10 years was not significantly associated with glycaemic control
Abera [88]	2022	Cross-sectional	Ethiopia	Type 2 diabetes	325	HbA1c	Good control (HbA1c < 7%); Poor control (HbA1c ≥ 7%)		Duration > 10 years	Odds Ratio	3.15	2.22	6.54	Significant association between diabetes duration of more than 10 years and poor glycaemic control
Abebe [89]	2022	Cross-sectional	Ethiopia	Type 2 diabetes; > 18 years	138	FBG	Controlled (FPG:80–130 mg/dl)		≥ 10 years	Odds Ratio	2.6	0.85	8.23	No significant relationship between duration of 10 years and more, and glycaemic control
									5–10 years		0.5	0.17	1.53	No significant relationship between duration of 5–10 years and glycaemic control
Fekadu [39]	2019	Cross-sectional	Ethiopia	Type 2 diabetes; 18–86 years	228	FBG	Good control (FBG: 70–130 mg/dL); poor control (< 70 mg/dL and > 130 mg/dL)	Duration of treatment	Treatment ˃ 10 years	Odds Ratio	3.94	1.51	27.83	Longer duration of diabetes treatment > 10 years was significantly associated with poor glycaemic control (p:0.012)
									Treatment 5–10 years		1.19	1.06	26.24	Duration of treatment of 5–10 years was not associated with glycaemic control (p:0.642)
Rwegerera [73]	2019	Cross-sectional	Botswana	Type 2 diabetes	368	HbA1c	desirable (< 7%); suboptimal (7–9%); poor (≥ 9%)		Treatment ˂ 3 years	Odds Ratio	1.54	0.68	3.45	Being on treatment at block 6 Clinic few than 3 yrs was not associated with glycaemic control
Shimels [75]	2018	Cross-sectional	Ethiopia	Type 2 diabetes; ≥ 18 years	361	FPG	Good control (FPG: 100–130 mg/dl)		Treatment ≥ 11 years	Odds Ratio	0.83	0.37	1.83	Treatment duration ≥ 11 yrs was not significantly associated with glycaemic control
									Treatment 6–10 years		0.82	0.43	1.56	Treatment duration 6–10 yrs was not significantly associated with glycaemic control
Yigazu [85]	2017	Cross-sectional	Ethiopia	Type 2 diabetes; 18–80 years	174	FBG	Controlled (average FBG: 80–130 mg/dL); Uncontrolled (average FBG > 130 or < 70 mg/dL)		Treatment ˂5 years	Odds Ratio	2.03	0.85	4.84	Duration of treatment < 5 years was not significantly associated with glycaemic control
Mphwanthe [61]	2020	Cross-sectional	Malawi	Type 2 diabetes; ≥ 25 years	428	HbA1c	Poor control (HbA1c ≥ 8%)	Self-report of glycaemic control	Fluctuating/unstable blood glucose levels	ß coefficient	1.760	1.479	2.041	Always having fluctuating/unstable blood glucose levels (p: < .001) was significantly associated with poor glycaemic control (p: < .001)
Mphwanthe [61]	2020	Cross-sectional	Malawi	Type 2 diabetes; ≥ 25 years	428	HbA1c	Poor control (HbA1c ≥ 8%)		No improvement of blood glucose levels from diagnosis	ß coefficient	0.968	0.621	1.307	Having blood glucose levels not improved from diagnosis was significantly associated with poor glycaemic control (p:0.004)
Mamo [53]	2019	Case–control	Ethiopia	Type 2 diabetes with poor glycemic control (cases) and without good glycemic control (controls); > 18 years	410	FBG	Good control (average fasting blood glucose of 80–130 mg/dL); poor control (average fasting blood glucose of > 130 mg/dL)	Presence of comorbidities	Presence of comorbidities	Odds Ratio	2.56	1.10	5.96	The presence of comorbidity was significantly associated with poor glycaemic control
Shimels [75]	2018	Cross-sectional	Ethiopia	Type 2 diabetes; ≥ 18 years	361	FPG	Glycemic control (FPG: 100–130 mg/dl)			Odds Ratio	0.16	0.09	0.27	The presence of comorbidity was significantly associated with poor glycaemic control
Tefera [77]	2010	Cross-sectional	Ethiopia	Type 2 diabetes; ≥ 18 years	400	FPG	Controlled (FPG:80–130 mg/dl)			Odds Ratio	0.33	0.15	0.73	The presence of morbidity was significantly associated with poor glycaemic control
Yosef [87]	2021	Cross-sectional	Ethiopia	Type 2 diabetes; ≥ 18 years	245	FBG	Good control (FBG:70–130 mg/dL)			Odds Ratio	1.61	0.8	3.22	The presence of comorbidity was significantly associated with poor glycaemic control
Botchway [31]	2021	Cross-sectional	Ghana	Type 2 diabetes; ≥ 18 years	254	HbA1c	No threshold	Number of comorbidities	Number of comorbidities	ß coefficient	0.15	− 0.13	0.43	The number of type 2 diabetes comorbidities was not significantly associated with glycaemic control
Otieno [69]	2017	Cross-sectional	Kenya	Type 2 diabetes; ≥ 30 years	220	HbA1c	Good control (HbA1c ≤ 7%); poor/suboptimal (HbA1c > 7%)		Having 1 comorbidity in comorbid depression	Odds Ratio	1.6	0.2	15.1	In patients with depression, having 1 comorbidity was not significantly associated with glycaemic control
									Having 2 or more in comorbid depression		1.4	0.4	5.7	In patients with depression, having 2 or more comorbidities was not significantly associated with glycaemic control
									Having 1 comorbidity in patients without depression		1.1	0.3	4.1	In patients without depression, having 1 comorbidity was not significantly associated with glycaemic control
									Having 2 or more in patients without depression		5.2	0.6	43.1	In patients wihtout depression, having 2 or more comorbidities was not significantly associated with glycaemic control
Achila [17]	2020	Cross-sectional	Eritrea	Type 2 diabetes; 20–88 years	309	HbA1c	Poor control (HbA1c ≥ 7%)	Hypertension	Absence of hypertension	Odds Ratio	1.94	1.06	3.56	The absence of hypertension was significantly associated with poor glycaemic control
BeLue [27]	2016	Cross-sectional	Senegal	Type 2 diabetes	106	HbA1c	Glycemic control (HbA1c < 7%)			ß coefficient	0.76	− 0.18	1.7	The absence of hypertension was not significantly with glycaemic control
Eticha [36]	2016	Cross- sectional	Ethiopia	Type 2 diabetes; ≥ 18 years	384	HbA1c	Good control (HbA1c < 7%); poor control (HbA1c ≥ 7%)		Presence of hypertension	Odds Ratio	2.2	1.1	4.4	The presence of hypertension was associated significantly with poor glycaemic control
Mobula [58]	2018	Cross- sectional	Ghana	hypertensive and Type 2 diabetes; ≥ 18 years	1226	HbA1c	Poor control (HBA1c ≥ 7%)			Odds Ratio	0.69	0.50	0.95	Dual diagnosis of diabetes and hypertension was significantly associated with good glycaemic control
Demoz [34]	2019	Cross-sectional	Ethiopia	Type 2 diabetes; ≥ 18 years	357	HbA1c and FBG	Adequate control ( average fasting blood glucose 70–130 mg/dL or HbA1c < 7%); Poor control (average fasting blood glucose > 130 or < 70 mg/dL or HbA1c > 7%)		Uncontrolled blood pressure	Odds Ratio	4.51	0.89	1.94	Uncontrolled BP was not found significantly associated with glycaemic control
Rwegerera [73]	2019	Cross-sectional	Ethiopia	Type 2 diabetes	368	HbA1c	Desirable (< 7%); suboptimal (7–9%); poor (≥ 9%)			Odds Ratio	1.04	0.67	1.59	Uncontrolled BP was not found significantly associated with glycaemic control
Anioke [22]	2019	Cross-sectional	Nigeria	Type 2 diabetes; ≥ 30 years	140	HbA1c	Good control (HbA1c < 7%); poor control (HbA1c ≥ 7%)		High systolic blood pressure	Odds Ratio	1.01	0.98	1.05	High systolic blood pressure was not significantly associated with glycaemic control
Mwita [65]	2019	Cross-sectional	Botswana	Type 2 diabetes; ≥ 18 years	500	HbA1c	Optimal glycaemic (HbA1c < 7%)		Optimal hypertension	Odds Ratio	1.61	0.63	4.13	Optimal hypertension control was not significantly associated with glycaemic control
Adeniyi [18]	2016	Cross-sectional	South Africa	Type 2 diabetes; ≥ 30 years at diagnostic of diabetes	327	HbA1c	Good control (HbA1c ≤ 7%); poor control (HbA1c > 7%); moderately poor (HbA1c = 7–8.9%); critically poor (HbA1c ≥ 9%)	Dyslipidemia	Elevated LDL	Odds Ratio	10.3	4.4	23.8	Elevated low-density lipoprotein cholesterol was significantly associated with poor glycaemic control
Demoz [34]	2019	Cross-sectional	Ethiopia	Type 2 diabetes; ≥ 18 years	357	HbA1c and FBG	Adequate control ( average fasting blood glucose 70–130 mg/dL or HbA1c < 7%); Poor control (average fasting blood glucose > 130 or < 70 mg/dL or HbA1c > 7%)		Poor lipid control	Odds Ratio	2.13	0.57	1.34	Poor lipid control was not significantly associated with glycaemic control
Eticha [36]	2016	Cross- sectional	Ethiopia	Type 2 diabetes; ≥ 18 years	384	HbA1c	Good control (HbA1c < 7%); poor control (HbA1c ≥ 7%)		Presence of dyslipidemia	Odds Ratio	1.5	0.8	2.8	Presence of dyslipidemia was not significantly associated with glycaemic control
Mamo [53]	2019	Case–control	Ethiopia	Type 2 diabetes with poor glycemic control (cases) and without good glycemic control (controls); > 18 years	410	FBG	Good control (average fasting blood glucose of 80–130 mg/dL); poor control (average fasting blood glucose of > 130 mg/dL)		High Total Cholesterol	Odds Ratio	3.62	1.46	8.97	Total cholesterol ≥ 200 mg/dl was significantly associated with poor glycaemic control
Mwita [65]	2019	Cross-sectional	Botswana	Type 2 diabetes; ≥ 18 years	500	HbA1c	Optimal glycaemic (HbA1c < 7%)		Optimal LDL-C control	Odds Ratio	2.20	0.64	7.57	Optimal LDL-C-control was not significantly associated with glycaemic control
Noor [66]	2017	Cross-sectional	Sudan	Type 2 diabetes	387	HbA1c	Controlled < 7% Uncontrolled > 7%		Elevated Triglycerides	Odds Ratio	0.879	0.353	2.188	TG level > 150 was not significantly associated with glycaemic control
Woldu [83]	2014	Cross-sectional	Ethiopia	Type 2 diabetes	102	FBG	Poor control (FBG level of > 126 mg/dl)		Hyperlipidemia	Odds Ratio	5	1.145	20.462	Hyperlipidemia was significantly associated with poor glycaemic control
Abebe [89]	2022	Cross-sectional	Ethiopia	Type diabetes, > 18 years	138	FBG	Good control (mean FBG:80–130 mg/dl); poor control (FBG < 80 mg/dl or > 130 mg/dl)	Body mass index	Obesity	Odds Ratio	4.1	1.2	14.11	Significant association between being obesity and poor glycaemic control
									Overweight	Odds Ratio	1.7	0.49	6.32	No significant association between being overweight and poor glycaemic control
Adeniyi [18]	2016	Cross-sectional	South Africa	Type 2 diabetes; ≥ 30 years at diagnostic of diabetes	327	HbA1c	Good control (HbA1c ≤ 7%); poor control (HbA1c > 7%); moderately poor (HbA1c = 7–8.9%); critically poor (HbA1c ≥ 9%)		Overweight/obesity	Odds Ratio	22.3	1.9	261.2	Overweight/obesity was significantly associated with poor glycaemic control
BeLue [27]	2016	Cross-sectional	Senegal	Type 2 diabetes	106	HbA1c	Glycemic control (HbA1c < 7%)		BMI	ß coefficient	− 0.10	− 0.17	− 0.03	BMI was not significantly associated with glycaemic control
Demoz [34]	2019	Cross-sectional	Ethiopia	Type 2 diabetes; ≥ 18 years	357	HbA1c and FBG	Adequate control ( average fasting blood glucose 70–130 mg/dL or HbA1c < 7%); Poor control (average fasting blood glucose > 130 or < 70 mg/dL or HbA1c > 7%)		Obesity	Odds Ratio	1.68	1.01	2.55	Obesity was singificantly associated with poor glycaemic control
									Overweight		3.51	1.82	4.01	Being overweight was not significantly associated with glycaemic control
Eticha [35]	2016	Cross- sectional	Ethiopia	Type 2 diabetes; ≥ 18 years	384	HbA1c	Good control (HbA1c < 7%); poor control (HbA1c ≥ 7%)		Normal BMI	Odds Ratio	0.3	0.1	1.8	Having a normal BMI was not significantly associated with glycaemic control
									Overweight		0.8	0.1	4.6	Being overweight was not significantly associated with glycaemic control
Kamuhabwa [47]	2014	Cross- sectional	Tanzania	Type 2 diabetes; ≥ 18 years	469	FBG	Good control (FBG ≤ 130 mg/dL or 7.2 mmol/L); poor control (FBG > 130 mg/dL or 7.2 mmol/L)		Obesity	Odds Ratio	2.347	1.274	4.324	Being obese was significantly associated with poor glycaemic control
									Normal BMI		2.234	1.278	3.904	Having a normal BMI was significantly associated with poor glycaemic control
Mphwanthe [61]	2020	Cross- sectional	Malawi	Type 2 diabetes; ≥ 25 years	428	HbA1c	HbA1c clinically elevated ≥ 8%		Underweight	ß coefficient	2.330	1.645	3.015	Underweight status was significantly associated with poor glycaemic control (p: .001)
									Overweight/obesity		− 0.143	− 0.419	0.133	Overweight/obese status was not significantly associated with glycaemic control (p: .605)
Otieno [69]	2017	Cross-sectional	Kenya	Type 2 diabetes; ≥ 30 years	220	HbA1c	Good control (HbA1c ≤ 7%); poor/suboptimal (HbA1c > 7%)		Overweight/obesity in patients with depression	Odds Ratio	1.7	0.4	6.5	In patients with depression, being overweight/obese (AOR:1.7 (0.4–6.5)) was not significantly associated with glycaemic control
									Overweight/obesity in patients without depression		2.0	1.0	4.0	In patients without depression, being overweight/obese was significantly associated with poor glycaemic control
Rwegerera [73]	2019	Cross-sectional	Botswana	Type 2 diabetes	368	HbA1c	Desirable (< 7%); suboptimal (7–9%); poor (≥ 9%)		Overweight/ Obese	Odds Ratio	1.38	0.53	3.59	Being overweight/obese was not significantly associated with glycaemic control
Yosef [87]	2021	Cross-sectional	Ethiopia	Type 2 diabetes; ≥ 18 years	245	FBG	Good control (FBG:70–130 mg/dL)		Overweight	Odds Ratio	2.6	1.32	5.1	Being overweight was not significantly associated with poor glycaemic control
									Obese	Odds Ratio	3.44	1.44	8.21	Being obese was not significantly associated with poor glycaemic control
Achila [17]	2020	Cross-sectional	Eritrea	Type 2 diabetes; 20–88 years	309	HbA1c	Poor control (HbA1c ≥ 7%)	WHR/WC	Abnormal WHR	Odds Ratio	3.01	1.15	7.92	Abnormal WHR was associated significantly with poor glycaemic control
Anioke [22]	2019	Cross-sectional	Nigeria	Type 2 diabetes; ≥ 30 years	140	HbA1c	Good control (HbA1c < 7%); poor control (HbA1c ≥ 7%)		Low WHR	Odds Ratio	2.65	0.42	16.88	Low WHR was not significantly associated with glycaemic control
									Medium WHR		1.82	0.47	6.98	Medium WHR was not significantly associated with glycaemic control
BeLue [27]	2016	Cross-sectional	Senegal	Type 2 diabetes	106	HbA1c	Glycemic control (HbA1c < 7%)		Waist circumference	ß coefficient	0.04	0.03	0.05	Waist circumference was not significantly associated with glycaemic control
Blum [30]	2019	Cross-sectional	D R Congo	Type 2 diabetes; ≥ 18 years	319	HbA1c	Poor control (HbA1c ≥ 9%)		Abdominal obesity	Odds Ratio	0.61	0.33	1.12	Abdominal obesity was not significantly associated with glycaemic control
Mamo [53]	2019	Case–control	Ethiopia	Type 2 diabetes with poor glycemic control (cases) and without good glycemic control (controls); > 18 years	410	FBG	Good control (average fasting blood glucose of 80–130 mg/dL); poor control (average fasting blood glucose of > 130 mg/dL)		High WHR	Odds Ratio	3.52	1.23	10.11	Waist to hip ratio of 0.9 or greater for male and 0.85 or greater for female was significantly associated with poor glycaemic control
Mashele [56]	2019	Cross-sectional	South Africa	Type 2 diabetes; 35–74 years	176	HbA1c	Optimal control (HbA1c < 7%); poor control (HbA1c ≥ 7%)		Increased WC	Odds Ratio	1.089	1.030	1.151	Increased waist circumference was significantly associated with poor glycaemic control
Adejumo [17]	2012	cross-sectional	Nigeria	type 2 diabetes with normal renal function; ≥ 18 years	72	HbA1c	good control (HbA1c ≤ 7.5%); poor control (HbA1c > 7.5%)	Other comorbidities	Incidence of anaemia	Odds Ration	10.8	1.28	91.20	Incidence of aneamia was significantly associated with poor glycemic Control (p < 0.05)
Afolabi [19]	2018	cross-sectional	Nigeria	type 2 diabetes; 40–80 years	80	HbA1c	Good control (< 7%); poor control (≥ 7%)		Presence of Non Alcoholic Fatty Liver Disease	Odds Ratio	3.473	1.017	11.864	Non alcoholic Fatty Liver Disease was sgnificantly associated with poor glycaemic control (p:0.047)
Akabwai [20]	2016	Cross-sectional	Uganda	Type 2 diabetes; ≥ 18 years	280	HbA1c	Good control (< 7%); poor control (≥ 7%)		Presence of vitamin B12 deficiency	Odds Ratio	3.29	1.44	7.51	Presence of vitamin B12 deficiency was significantly associated with poor glycaemic control (p: 0.005))
Akpalu [21]	2018	Cross-sectional	Ghana	Type 2 diabetes; 30–65 years	400	HbA1c	Good control (HbA1c < 7%)		Presence of depression	Odds Ratio	1.04	0.85	2.29	Presence of depression was not significantly associated with glycaemic control
Biadgo [28]	2018	Cross- sectional	Ethiopia	Type 2 diabetes	159	FBS	Adequate control (FBS ≤ 130 mg/dl); poor control (FBS > 130 mg/dl)		Metabolic syndrome	Odds Ratio	2.53	1.01	6.32	Metabolic syndrome (IDF criteria) was significantly associated with poor glycaemic control
Dagnew [33]	2017	Comparative Cross-sectional	Ethiopia	Type 2 diabetes and healthy relatives; ≥ 30 years	210	FBG	FBG < 126 mg/dl versus FBG ≥ 126 mg/dl		Presence of cognitive impairment	Odds Ratio	4.43	1.14	17.18	Poor glycaemic control was significantly associated with the presence of cognitive impairment
Kalain [46]	2015	Cross- sectional	South Africa	Type 2 diabetes; ≥ 18 years	200	HbA1c	Good control (HbA1c < 7%)		Presence of Congestive Cardiac Failure (CCF)	Odds Ratio	3.172564			Presence of CCF was associated with glycaemic control (p:0.035); 95% CI not provided
Khoza [50]	2018	Case control	South Africa	Type 2 diabetes; > 30 years	320	HbA1c and glucose	No threshold		HIV infection	β coefficient	0.040			HIV-positive was significantly associated with poor glycaemic control (p:0.01); 95% CI not provided
										Odds Ratio	4.29	1	18.32	HIV-negative status was not associated with glycaemic control
Sarfo-Kantanka [74]	2017	Case–control	Ghana	Type 2 diabetes; 40–80 years	302	FBG and HbA1c	No threshold		Thyroid autoimmunity	Odds Ratio	1.46	1.23	1.73	Poor glycaemic control (p < 0.0001) was significantly associated with thyroid autoimmunity (p < 0.0001)
Woldu [83]	2014	Cross-sectional	Ethiopia	Type 2 diabetes	102	FBG	Poor control (FBG level of > 126 mg/dl)		Presence of dyspesia	Odds Ratio	4	0.026	6.000	Presence of dyspepsia was not significantly associated with glycaemic control
Demoz [34]	2019	Cross-sectional	Ethiopia	Type 2 diabetes; ≥ 18 years	357	HbA1c and FBG	Adequate control ( average fasting blood glucose 70–130 mg/dL or HbA1c < 7%); Poor control (average fasting blood glucose > 130 or < 70 mg/dL or HbA1c > 7%)	Complications	Presence of complications	Odds Ratio	2.0	0.69	1.06	Presence of complications was not associated with glycaemic control
Rwegerera [73]	2019	Cross-setctional	Ethiopia	Type 2 diabetes	368	HbA1c	desirable (< 7%); suboptimal (7–9%); poor (≥ 9%)			Odds Ratio	0.76	0.44	1.3	Absence of complication was not associated with glycaemic control
Tefera [77]	2020	Cross-sectional	Ethiopia	Type 2 diabetes; ≥ 18 years	400	FPG	Controlled (FPG:80–130 mg/dl)			Odds Ratio	0.77	0.26	2.34	Presence of complications was not significantly associated with glycaemic control
Eticha [36]	2016	Cross-sectional	Ethiopia	Type 2 diabetes; ≥ 18 years	384	HbA1c	Good control (HbA1c < 7%); poor control (HbA1c ≥ 7%)		Presence of renal disease	Odds Ratio	0.9	0.4	2.2	Presence of renal disease was not significantly associated with glycaemic control
Eticha [36]	2016	Cross-sectional	Ethiopia	Type 2 diabetes; ≥ 18 years	384	HbA1c	Good control (HbA1c < 7%); poor control (HbA1c ≥ 7%)		Presence of DKA	Odds Ratio	2.6	0.9	7.1	Presence of DKA was not significantly associated with glycaemic control
Woldu [83]	2014	Cross-sectional	Ethiopia	Type 2 diabetes	102	FBG	Poor control (FBG level of > 126 mg/dl)			Odds Ratio	0.6	0.015	22.402	Presence of DKA was not significantly associated with glycaemic control
Eticha [36]	2016	Cross-sectional	Ethiopia	Type 2 diabetes; ≥ 18 years	384	HbA1c	Good control (HbA1c < 7%); poor control (HbA1c ≥ 7%)		Presence of peripheral neuropathy	Odds Ratio	1.1	0.5	2.2	Presence of peripheral neuropathy was not significantly associated with glycaemic control
Woldu [83]	2014	Cross-sectional	Ethiopia	Type 2 diabetes	102	FBG	Poor control (FBG level of > 126 mg/dl)			Odds Ratio	579	116.8	2870	Presence of peripheral neuropathy was significanty associated with poor glycaemic control
Mohamed [59]	2013	Case–control	Sudan	Type 2 diabetes and non-diabetic controls	457	HbA1c	Well-controlled (HbA1c < 8%); poorly controlled (HbA1c > 8%)		High levels of mobility index	Odds Ratio	2.60	1.21	5.55	Poor glycaemic control was significantly associated with high levels of mobility index
Gebremedhin [42]	2019	Cross-sectional	Ethiopia	Type 2 diabetes; ≥ 18 years	267	FBG	No threshold	Examination findings	Health related quality of life	ß coefficient	− 0.08	− 0.12	− 0.04	Overall health related quality of life was inversely associated and FBG (p < 0.001)
Oyewole [70]	2019	Cross-sectional	Nigeria	Type 2 diabetes; ≥ 21 years	162	HbA1c and FBG	No threshold		Global disability burden	ß coefficient	0.061	0.057	0.067	Disability burden was significantly associated with poor glycaemic control (p: 0.0001)
Ramkisson [72]	2016	Cross-sectional	South Africa	Type 2 diabetes; ≥ 18 years	401	HbA1c	No threshold		Diabetes related distress	Odds Ratio	1.04	1.00	1.09	Diabetes related distress was not significantly associated with glycaemic control
Achila [17]	2020	Cross-sectional	Eritrea	Type 2 diabetes; 20–88 years	309	HbA1c	Poor control (HbA1c ≥ 7%)	Laboratory findings	estimated Glomerular Filtration Rate (eGFR)	Odds Ratio	0.99	0.98	1	A unit reduction in eGFR was also associated with HbA1c ≥ 7% (p:0:031))
Mwita [65]	2019	Cross-sectional	Botswana	Type 2 diabetes; ≥ 18 years	500	HbA1c	Optimal glycaemic (HbA1c < 7%)			Odds Ratio	1.00	0.99	1.02	No significant association between eGFR and glycaemic control (p: 0.412)
Inih [45]	2018	Cross-sectional	Nigeria	Type 2 diabetes; men	164	HbA1c	Poor control (HbA1c > 7%)		Hypogonadism	Odds Ratio	3.85	0.13	0.55	Poor glycaemic control was significantly associated with hypogonadism (p < 0.001)
									Low sperm count		0.118	− 5.85	5.61	No significant association between low sperm count with HbA1c (p:0.97)

*HbA1c* Haemoglobin A1c, *FBG* Fasting blood glucose, *FBS* Fasting blood sugar, *FPG* Fasting plasma glucose

**Table 5:** Adherence to treatment plans and glycaemic control in sub-Saharan Africa

First author name	Year	Study design	Study setting	Study population	Sample size	Measurement of glycemic control	Definition of glycemic control	Generic factor	Specific factors	Measure of association	Point estimate	Lower bound	Upper bound	Association with glycemic control
Yigazu [85]	2017	Cross-sectional	Ethiopia	Type 2 diabetes; 18–80 years	174	FBG	Controlled (average FBG: 80–130 mg/dL); Uncontrolled (average FBG > 130 or < 70 mg/dL)	Adherence to follow-up	Adherence to regular follow-up	Odds Ratio	2.42	1.08	5.44	Adherence to regular follow-up was associated with good glycaemic control
Kefale [49]	2019	Case–control	Ethiopia	Type 2 diabetes; ≥ 18 years	169	FBS	glycemic control (FBS ≤ 130 mg/dl in all these most three recent measurements)		Having regular diabetic care of ˃ 1 month	Odds Ratio	0.4	0.2	0.9	Having regular diabetic care follow up of every > 1 month was negatively associated with poor glycaemic control
Abebe [89]	2022	Cross-sectional	Ethiopia	Type 2 diabetes; > 18 years	138	FBG	Good control (mean FBG:80–130 mg/dl); poor control (FBG < 80 mg/dl or > 130 mg/dl)	Adherence to treatment	Drug adherence	Odds Ratio	3.08	1.22	7.08	Absence of drug adherence was significantly associated with poor glycaemic control
Demoz [34]	2019	Cross-sectional	Ethiopia	Type 2 diabetes; ≥ 18 years	357	HbA1c and FBG	Adequate control ( average fasting blood glucose 70–130 mg/dL or HbA1c < 7%); Poor control (average fasting blood glucose > 130 or < 70 mg/dL or HbA1c > 7%)		Low medication adherence	Odds Ratio	5.10	1.18	6.55	Low adherence was significanty associated with poor glycaemic control
Kamuhabwa [47]	2014	Cross-sectional	Tanzania	Type 2 diabetes; ≥ 18 years	469	FBG	Good control (FBG ≤ 130 mg/dL or 7.2 mmol/L); poor control (FBG > 130 mg/dL or 7.2 mmol/L)		Low medication adherence	Odds Ratio	2.084	1.069	4.060	Low medication adherence was significantly associated with poor glycaemic control
Kassahun [48]	2016	Cross-sectional	Ethiopia	Type 2 diabetes; ≥ 18 years	309	FBG	Poor control (mean FBG > 130 mg/dl)		Low medication adherence	Odds Ratio	5.08	2.02	12.79	Low adherence was significantly associated with poor glycaemic control
									Medium medication adherence		3.49	1.72	7.09	Medium adherence was significantly associated with poor glycaemic control
Yimam [86]	2020	Cross-sectional	Ethiopia	Type 2 diabetes with hypertension; ≥ 18 years	300	FBG	Good control (mean FBG:80–130 mg/dl); poor control (FBG < 80 mg/dl or > 130 mg/dl)		Low medication adherence	Odds Ratio	4.26	1.70	10.65	Low adherence to treatment was significantly associated with poor glycaemic control
									Medium medication adherence		2.43	0.97	6.07	Medium adherence to medication was not associated with glycaemic control
Mamo [53]	2019	Case–control	Ethiopia	Type 2 diabetes with poor glycemic control (cases) and without good glycemic control (controls); > 18 years	410	FBG	Good control (average fasting blood glucose of 80–130 mg/dL); poor control (average fasting blood glucose of > 130 mg/dL)		No adherence to medication	Odds Ratio	0.67	0.26	1.69	No adherence to antidiabetic medication was not associated with glycaemic control
Fseha [40]	2017	Cross-sectional	Ethiopia	Type 2 diabetes; 22–60 years	200	FBS	Good (FBS 70–130 mg/dl), Poor (FBS > 130 mg/dl)		Good medication adherence	Odds Ratio	2.033	1.025	4.034	Medical adherence was associated with good glycaemic control (p:0.001)
Shimels [75]	2018	Cross-sectional	Ethiopia	Type 2 diabetes; ≥ 18 years	361	FPG	glycemic control (FPG: 100–130 mg/dl)		Good medication adherence	Odds Ratio	1.38	0.62	3.09	Good medical adherence was not associated with glycaemic control
Tefera [77]	2020	Cross-sectional	Ethiopia	Type 2 diabetes; ≥ 18 years	400	FPG	Controlled (FPG:80–130 mg/dl)		Good medication adherence	Odds Ratio	1.61	1.04	4.79	Good adherence level was significantly associated with good glycaemic control
									Intermediate adherence		0.19	0.06	0.59	Intermediate adherence was not associated with glycaemic control
Teklay [79]	2013	Cross-sectional	Ethiopia	Type 2 diabetes; ≥ 18 years	267	FBG	Controlled (average FBG: 70–130 mg/dl), Uncontrolled (average FBG > 130 mg/dl)		Good medication adherence		1.469	0.732	2.949	Poor glycaemic control was not associated with adherence to treatment (p:0.280)
Id [44]	2021	Cross-sectional	Ethiopia	Type 2 diabetes; > 18 years	394	FBS	Good blood glucose control (< 154 mg/dl); Poor blood glucose control (≥ 154 mg/dl)	Practice of recommendations	Poor level of practice	Odds Ratio	1.693	1.126	2.545	Poor level of practice was significantly associated with poor glycaemic control

**Table 6:** Treatment modalities and glycaemic control in sub-Saharan Africa

First author name	Year	Study design	Study setting	Study population	Sample size	Measurement of glycemic control	Definition of glycemic control	Generic factor	Specific factors	Measure of association	Point estimate	Lower bound	Upper bound	Association with glycemic control
Mobula [58]	2018	Cross-sectional	Ghana	Hypertensive and Type 2 diabetes; ≥ 18 years	1226	HbA1c	Poor control (HBA1c ≥ 7%)	Number of diabetes medicine	Number of diabetes medicines	Odds Ratio	1.73	1.45	2.07	number of diabetes medicines was associated with poor glycaemic control
Biru [29]	2017	Cross-sectional	Ethiopia	Type 2 diabetes; ≥ 18 years	322	FBG	Good control (FBG ≤ 110 mg/dL)		2	Odds Ratio	1.30	0.26	6.54	Being on 2 antidiabetes medications was not associated with glycaemic control
									3		1.26	0.22	7.33	Being on 3 antidiabetes medications was not associated with glycaemic control
									4		1.56	0.29	8.32	Being on 4 antidiabetes medications was not associated with glycaemic control
									5		3.16	0.59	16.96	Being on 5 antidiabetes medications was not associated with glycaemic control
									≥ 5		1.18	0.19	7.14	Being on ˃ 5 antidiabetes medications was not associated with glycaemic control
Rwegerera [73]	2019	Cross-sectional	Ethiopia	Type 2 diabetes	368	HbA1c	Desirable (< 7%); suboptimal (7–9%); poor (≥ 9%)		2 Oral hypoglycemic agents	Odds Ratio	0.47	0.18	1.2	Being on two oral hypoglycemic agents was not associated with glycaemic control
Kefale [49]	2019	Case–control	Ethiopia	Type 2 diabetes; ≥ 18 years	169	FBS	Good control (FBS ≤ 130 mg/dl)		Addition of a second antidiabetic medication	Odds Ratio	2.9	1.3	6.2	Addition of a second antidiabetic medication was associated with poor glycaemic control
Kamuhabwa [47]	2014	Cross-sectional	Tanzania	Type 2 diabetes; ≥ 18 years	469	FBG	Good control (FBG ≤ 130 mg/dL or 7.2 mmol/L); poor control (FBG > 130 mg/dL or 7.2 mmol/L)	Type of diabetes regimen	Combinaison therapy of Oral Hypoglycemic Agents (OHA)	Odds Ratio	2.528	1.475	4.531	Being on combinaison therapy of Oral Hypoglycemic Agents was associated with poor glycaemic control
Kassahun [48]	2016	Cross-sectional	Ethiopia	Type 2 diabetes; ≥ 18 years	309	FBG	Poor control (mean FBG > 130 mg/dl)		Insulin and oral medication	Odds Ratio	4.59	1.05	20.14	Taking Insulin and oral medication was significantly associated with poor glycaemic control
									Insulin alone		1.77	0.60	5.19	Use of insuline alone was not significantly associated with glycaemic control
Mamo [53]	2019	Case–control	Ethiopia	Type 2 diabetes with poor glycemic control (cases) and without good glycemic control (controls); > 18 years	410	FBG	Good control (average fasting blood glucose of 80–130 mg/dL); poor control (average fasting blood glucose of > 130 mg/dL)		Insulin	Odds Ratio	4.48	1.52	13.16	Being on insulin was significantly associated with poor glycaemic control
									Metformin plus Glibenclamide		9.22	2.90	29.35	Being on metformin plus Glibenclamide was significantly associated with poor glycaemic control
									Glibenclamide alone		3.57	0.18	68.66	Being on glibenclamide alone was not significantly associated with glycaemic control
									Metformin plus Insulin		3.73	0.87	16.05	Being on metformin plus insulin was not significantly associated with glycaemic control
Mwita [65]	2019	Cross-sectional	Botswana	Type 2 diabetes; ≥ 18 years	500	HbA1c	Optimal glycaemic (HbA1c < 7%)		Insulin plus OHA	Odds Ratio	0.34	0.07	1.70	Use of insulin plus OHA was not significantly associated with glycaemic control
									OHA alone		0.90	0.46	1.74	Use of OHA alone was not significantly associated with glycaemic control
Id [44]	2021	Cross-sectional	Ethiopia	Type 2 diabetes; > 18 years	394	FBS	Good blood glucose control (< 154 mg/dl); poor blood glucose control (≥ 154 mg/dl)		Insulin plus Oral Antidiabetic Drugs (OAD)	Odds Ratio	2.177	1.104	4.294	Being on Insulin plus OAD was significantly associated with poor glycaemic control
									Insuline alone		0.87	0.54	1.37	Being treated with Insulin alone was not significantly associated with glycaemic control
Otieno [69]	2017	Cross-sectional	Kenya	Type 2 diabetes; ≥ 30 years	220	HbA1c	Good control (HbA1c ≤ 7%); poor/suboptimal (HbA1c > 7%)		Insuline alone or combined with OADs	Odds Ratio	20.0	2.4	167.4	For the patients with comorbid depression, being on insulin therapy alone or combined with OADs was associated with poor glycaemic control
									Insulin alone or combined with OADs		1.9	0.9	3.7	For the patients without depression, being on insulin therapy alone or combined with OADs was not associated with poor glycaemic control
Rwegerera [73]	2019	Cross-sectional	Ethiopia	Type 2 diabetes	368	HbA1c	Desirable (< 7%); suboptimal (7–9%); poor (≥ 9%)		Diet/Oral hypoglycemic agents	Odds Ratio	6.41	2.28	18	Being on diet/oral hypoglycemic agents was significantly associated with good glycaemic control
Shimels [75]	2018	Cross-sectional	Ethiopia	Type 2 diabetes; ≥ 18 years	361	FPG	Good control (FPG: 100–130 mg/dl)		Insulin plus oral combinaison therapy	Odds Ratio	0.33	0.14	0.77	Being on insulin plus oral combination therapy was significantly associated with good glycaemic control
									Oral combinaison therapy		0.37	0.18	0.76	Being on oral combination therapy was significantly associated with good glycaemic control
Tekalegn [78]	2018	Cross-sectional	Ethiopia	Type 2 diabetes; ≥ 15 years	422	FBG	Good control (average FBG:70–130 mg/dL); poor control (average FBG > 130 or < 70 mg/dL)		Insulin alone	Odds Ratio	3.01	1.5	5.99	Being on insulin was significantly associated with poor glycaemic control
									Insuline and OADs		1.2	0.24	6.27	Being on insulin and OHA was not associated with glycaemic control
									Diet only		2.9	0.86	9.9	Being on diet only was not associated with glycaemic control
Abera [88]	2022	Cross-sectional	Ethiopia	Type 2 diabetes	325	HbA1c	Poor control (HBA1c ≥ 7%)		Insulin	Odds Ratio	3.07	2.1	6.12	Significant association between being on insulin and poor glycaemi control
									OHA& Insulin		2.36	0.82	5.97	
									Diet/exercise		0.91	0.52	3.53	
Yimam [86]	2020	Cross-sectional	Ethiopia	Type 2 diabetes with hypertension; ≥ 18 years	300	FBG	Good control (mean FBG:80–130 mg/dl); poor control (FBG < 80 mg/dl or > 130 mg/dl)	Side-effects	Presence of Drug Related Problems	Odds Ratio	2.29	1.20	4.39	Having drug related problems was found significantly associated with poor glycaemic control
Khoza [50]	2018	Case–control	South Africa	Type 2 diabetes; > 30 years	320	HbA1c and glucose	No threshold	Treatment for comorbid conditions	Use of statins	ß coefficient	0.030			The use of statins was significantly correlated with higher HbA1c level (p:0.002); 95% CI not provided
Khoza [50]	2018	Case–control	South Africa	Type 2 diabetes; > 30 years	320	HbA1c and glucose	No threshold		Use of Anti-HT	ß coefficient	0.069			The use of anti hypertensive treatment was significantly correlated with glucose level (p:0.0006); 95% CI not provided
Anyanwu [23]	2016	Randomized controlled trial	Nigeria	Type 2 diabetes with poor glycemic control and vitamin D deficiency; 35–65 years	42	HbA1c	No threshold		Effect of Vitamin D supplementation	Mean difference	− 0.66	− 2.66	1.34	No significant drop in the mean HbA1c level in the treatment group after 12 weeks of Vitamin D3 supplementation compared to the placebo group
Tsobgny-Tsague [82]	2018	Randomized controlled trial	Cameroon	Type 2 diabetes with poor glycaemic control and moderate to severe chronic periodontitis	34	HbA1c	No threshold		Nonsurgical periodontal treatment	Mean difference	3.0	0.6	5.4	Significant reduction in HbA1c in the treatment group at 3 months
Adeniyi [18]	2016	Cross-sectional	South Africa	Type 2 diabetes; ≥ 30 years at diagnostic of DM	327	HbA1c	Good control (HbA1c ≤ 7%); poor control (HbA1c > 7%); moderately poor (HbA1c = 7–8.9%); critically poor (HbA1c ≥ 9%)	Information on diabetes	Source of diabetes information	Odds Ratio	3.2	1.4	7.0	Diabetes information from non-health workers was significantly associated with poor glycaemic control
Tefera [77]	2020	Cross-sectional	Ethiopia	Type 2 diabetes; ≥ 18 years	400	FPG	Controlled (FPG:80–130 mg/dl)		Diabetes health literacy	Odds Ratio	1.85	1.09	3.40	Patients with high diabetes literacy were 1.85 times more likely to achieve glycemic targets than lower diabetic literacy patients
Yosef [87]	2021	Cross-sectional	Ethiopia	Type 2 diabetes; ≥ 18 years	245	FBG	Good control(FBG:70–130 mg/dL)		Diabetes Health education at Health Institution	Odds Ratio	1.21	0.67	2.18	Absence of Diabetes Health education at Health Institution was not associated with glycaemic control
Id [44]	2021	Cross-sectional	Ethiopia	Type 2 diabetes; > 18 years	394	FBS	Good blood glucose control (< 154 mg/dl); Poor blood glucose control (≥ 154 mg/dl)	Pharmacist advice	No clarity of pharmacist's advice	Odds Ratio	1.857	1.100	3.132	No Clarity of Pharmacist’s advice about drug was significantly associated with poor glycaemic control
Abera [88]	2022	Cross-sectional	Ethiopia	Type 2 diabetes	325	HbA1c	Poor control (HBA1c ≥ 7%)	Definition of glycaemic goal	Failure to set glycaemic goal	Odds Ratio	3.42	2.17	5.97	Failure to set glycaemic goal was significantly associated with poor glycaemic control

**Table 7:** Glycaemic optimization interventions and glycaemic control in sub-Saharan Africa

First author name	Year	Study design	Study setting	Study population	Sample size	Measurement of glycemic control	Definition of glycemic control	Generic factor	Specific factors	Measure of association	Point estimate	Lower bound	Upper bound	Association with glycemic control
Mash [55]	2014	Pragmatic randomized controlled trial	South Africa	Type 2 diabetes	422	HbA1c	No threshold	Educational intervention	Effectiveness of group education	Mean difference	0.01	− 0.2	0.28	No significant difference between the intervention and control groups in reduction of HbA1c level by 1%
Thuita [81]	2020	Randomized controlled trail	Kenya	Type 2 diabetes; 20–79 years	153	HbA1C and FBG	Good control (HbA1c < 7%); poor control (HbA1c > 7%)		Effect of a nutrition education programme on the Metabolic syndrome in type 2 diabetes patients	Odds Ratio	2.04	0.84	4.92	No significant difference in the prevalence of high HbA1c between groups at six months post intervention (Nutrition education group versus Control)
											2.08	0.85	5.09	No significant difference in the prevalence of high HbA1c between groups at six months post intervention (Nutrition education peer to peer support group (NEP) versus Control)
Gathu [41]	2018	Randomized controlled trial	Kenya	Sub-optimally controlled Type 2 diabetes; 18–65 years	140	HbA1c	No threshold		Diabetes self-management education	Mean difference	0.37	− 0.45	1.19	No significant difference in the primary outcome (HbA1c) between the two groups
Muchiri [63]	2016	Randomized controlled trial	South Africa	type 2 diabetes; 40–70 years	82	HbA1c	No threshold		Effect of a participant-customised nutrition education programme	Mean difference	− 0.64	− 0.19	1.15	No significant difference between the intervention and control groups for HbA1c at 6 months (p = 0·13)
											− 0.63	− 0.26	1.5	No significant difference between the intervention and control groups for HbA1c at 12 months (p:0.16)
Hailu [43]	2018	Controlled before-and-after study	Ethiopia	Type 2 diabetes mellitus; > 30 years	220	FBS	No threshold		Nurse-led diabetes self-management education	Mean difference	27	17	37	Significant difference in the intervention group compared to the control group at 9 months
Fseha [40]	2017	Cross-sectional	Ethiopia	Type 2 diabetes; 22–60 years	200	FBS	Good (FBS 70–130 mg/dl), Poor (FBS > 130 mg/dl)	Self-management of diabetes	Home glucose monitoring	Odds Ratio	1.697	0.852	3.353	Home glucose monitoring was not associated with glycaemic control
Fseha [40]	2020	Cross-sectional	Malawi	Type 2 diabetes; ≥ 25 years	428	HbA1c	Poor control (HbA1c clinically elevated ≥ 8%)		Additional blood glucose monitoring	ß coefficient	− 0.359	− 0.609	− 0.149	Additional blood glucose monitoring at private clinic/home/diabetes peer groups was not associated with glycaemic control
Mwavua [64]	2016	Cross-sectional	Kenya	Type 2 diabetes; ≥ 18 years	200	HbA1c	Good control (HbA1c < 7%); poor control (HbA1c ≥ 7%)		Monthly self-monitoring	Odds ratio	2.6	0.6	10.7	Monthly self-monitoring was not significantly associated with glycaemic control
									Absence of self-monitoring	Odds Ratio	3.3	0.8	12.6	Absence of self-monitoring was not associated with glycaemic control
Maharaj [52]	2016	randomized controlled trial	Nigeria	Non-insulin dependent type 2 diabetes; 30–58 years	90	HbA1c	No threshold	Exercise programs	Effect of rebound exercise	Mean difference	0.904	0.832	0.985	Significant improvements at 9 weeks post-intervention in mean HbA1c in the exercise group
Yan [84]	2014	Randomized controlled trial	Mozambique	Type 2 diabetes; 40–70 years	41	HbA1c	No threshold		Effect of Aerobic Training	Mean difference	− 1.0	− 1.3	− 0.7	Significant reduction of plasma glucose at 120 min (Glu 120) following glucose load in the exercise group after training
Siddiqui [76]	2018	Quasi-experimental study	South Africa	Type 2 diabetes; 18–65 years	95	HbA1C	No threshold		Level of physical activity measured with a pedometer	Mean difference	− 1.0420	− 1.2225	− 0.86	Change in HbA1c over the three-month period was significant in the intervention group
Fayehun [38]	2018	Randomized trial	Nigeria	Type 2 diabetes; 33–64 years	46	HbA1c	No threshold		Physical activity with a 10,000 steps each day	Mean difference	− 0.74	− 1.32	− 0.02	Endline HbA1c was lower in the intervention group than in the control group
Ezema [37]	2014	Randomized trial	Nigeria	Type 2 diabetes; 40–55 years	54	FBS	No threshold		Aerobic exercise training (V02 max)	Pearson product moment correlation test	− 0.252			Significant effect of the exercise training program on FBS (p:0.001); 95% CI not provided
Mayet [57]	2012	Quasi-experimental study	South Africa	Type 2 diabetes	600	HbA1c	No threshold	Medical intervention	Insulin therapy initiation	Paired t-test				Mean HbA1c at insulin initiation was 10.29% (± 2.42), and 10.63% (± 1.93) after adjustment of insulin dose (p-value > 0.05)
Rambiritch [71]	2014	A 12-week, prospective, single-center, open-label, dose-escalation study	South Africa	Poorly controlled type 2 diabetes requiring oral antidibetic medications; > 20 years	22	FBG	No threshold		Dose escalation of Glibenclamide	Analysis of variance	19.61			Significant decrease in percentage of glucose from dose zero to 2.5 mg (P ≤ 0.001); no significant decrease for the 2.5–5 mg, 5–10 mg, and 10–20 mg doses. 95% CI provided
Assah [24]	2015	Non-randomized controlled trial	Cameroon	Type 2 diabetes	192	HbA1c	No threshold	Multi level peer support	Effectiveness of a community-based multilevel peer support intervention	Mean difference	− 1.7	− 2.2	− 1.3	Significant reduction in HbA1c in the intervention group compared with controls
Mash [54]	2016	Quasi-experimental study	South Africa	Type 2 diabetes; > 18 years	600	HbA1c	No threshold	Laboratory testing	Introducing point-of-care (POC) testing for HbA1c	Mean difference	0.00	-1.5	1.5	No significant difference in Mean difference in HbA1c result (%) at 18 months in the intervention group

#### Sociodemographic characteristics

Table 2 presents the sociodemographic factors with respect to their relationship with glycaemic control. Five studies assessed the relationship between increasing age and glycaemic control [27, 31, 34, 58, 61], two found that it was negatively associated with glycosylated haemoglobin [31, 61], and one found that it was associated with good glycaemic control [57]. Older age was associated with poor glycaemic control in twelve studies [22, 29, 32, 36, 39, 65, 68, 69, 73, 77, 83, 86]. Eight studies assessed the relationship between the female gender and glycaemic control [18, 29, 34, 51, 61, 64, 65, 73], two studies found that the female gender was significantly associated with poor glycaemic control [18, 34], and one study linked it to good glycaemic control [28]. Male gender with respect to glycaemic control was assessed by eleven studies [27, 31, 39, 44, 58, 66, 75, 77, 83, 85, 87]; two studies associated it with good glycaemic control [58, 75], while two studies linked it to poor glycaemic control [27, 87]. Fifteen studies assessed the relationship between educational level and glycaemic control; in one study, primary, secondary, or tertiary education levels were associated with good glycaemic control [28]. A lack of formal education and a low level of education were associated with poor glycaemic control in three studies [39, 48, 87]. In respectively two studies, low monthly income [18, 87], absence of health insurance [47, 58], and being a farmer [25, 48] were associated with poor glycaemic control. In respectively one study, living in urban areas [49], and a high frequency of seeking traditional medicine practitioners [30] were associated with poor glycaemic control. Residing less than 100 kms from a health facility [25], residing in Guinea compared to residing in Cameroon [32], self-reporting a positive perception of family support [68], and the frequency of participating in religious activities [31] were associated with good glycaemic control in respectively one study.

#### Lifestyle factors

The lifestyle factors assessed were dietary adherence, the practice of exercise, smoking, and alcohol consumption (Table 3). Good dietary adherence was associated with good glycaemic control in five studies [29, 36, 40, 61, 86] while low adherence to dietary recommendations was associated with poor glycaemic control in two studies [35, 67]. Exercise was associated with good glycaemic control in two studies [29, 36]. The inadequate practice of exercise was associated with poor glycaemic control in two studies [39, 53]. In respectively one study, smoking [39], and alcohol consumption [29] were associated with poor glycaemic control.

#### Clinical factors

The clinical factors—history of diabetes and comorbidities—with respect to glycaemic control are summarized in Table 4. A family history of diabetes was significantly associated with poor glycaemic control in one study [80]. A long duration of diabetes was associated with poor glycaemic control in seven studies [18, 26, 27, 32, 58, 61]. As a corollary, treatment of > 10 years was associated with poor glycaemic in one study [38]. In one study, patients who always had fluctuating/unstable blood glucose levels or had blood glucose levels not improved from diagnosis were prone to poor glycaemic control [60].

Four studies found that the presence of comorbidities was associated with poor glycaemic control [53, 75, 77, 87]. The presence of hypertension led to poor glycaemic control in one study [16]. Dyslipidaemia was associated with poor glycaemic control in three studies [18, 53, 82]. Concerning body mass index (BMI), all categories, such as being underweight [60], having a normal BMI [46], or being overweight/obese [18, 34, 87] have been significantly associated with poor glycaemic control. Central obesity was associated with poor glycaemic control in four studies [16, 30, 52, 56]. In respectively one study, the presence of anaemia [17], non-alcoholic fatty liver disease [19], vitamin B12 deficiency [20], metabolic syndrome [28], cognitive impairment [32], congestive cardiac failure [46], HIV infection [46], thyroid autoimmunity [74], and hypogonadism [45] had a significant association with poor glycaemic control. The presence of peripheral neuropathy [83] or a high-level tooth mobility index [59] was associated with poor glycaemic control. Overall health-related quality of life was inversely associated with FBG [42]. The global disability burden was significantly associated with poor glycaemic control [70]. A unit reduction in the estimated glomerular filtration rate (eGFR) was also associated with HbA1c ≥ 7% [16].

#### Adherence to treatment plans

Adherence modalities, as represented by adherence to scheduled appointments or medication adherence, are presented in Table 5. Regular attendance at scheduled appointments was associated with good glycaemic control in two studies [49, 85]. Good medication adherence was associated with good glycaemic control in two studies [40, 77], while two other studies showed no association [75, 78]. Low medication adherence had a significant association with poor glycaemic control in three studies [33, 48, 86]. Medium medication adherence was associated with poor glycaemic control in one study [48].

#### Treatment modalities

The findings on the treatment modalities with respect to glycaemic control are summarized in Table 6. The pill burden was associated with poor glycaemic control in one study. Combination therapy with oral hypoglycaemic agents (OHA) was associated with poor glycaemic control in two studies [48, 53] while it was linked to good glycaemic control in one study [75]. Insulin plus OHA was associated with poor glycaemic control in three studies [44, 48, 69], while it was linked to good glycaemic control in one study [75]. The use of insulin alone was associated with poor glycaemic control in two studies [53, 78]. The presence of drug-related problems was associated with poor glycaemic control as shown in one study [86]. Rwegerera et al. found that being on diet and OHA was associated with suboptimal glycaemic control [73]. A South African study found that the use of statin and antihypertensives was associated with higher glycaemic levels [50]. Non-surgical periodontal management was associated with good glycaemic control after three months in one study [82]. Diabetes information from non-health workers was significantly associated with poor glycaemic control [18], while having a high diabetes health literacy [77] was significantly associated with good glycaemic control. In one study, the absence of clarity in pharmacists’ advice was associated with poor glycaemic control [44].

### Reported glycaemic control optimization interventions

The interventions retrieved from the included studies are presented along with their effect on glycaemic control in Table 7. Only one study [43] out of five reported an educational program associated with good glycaemic control. None of the self-management programs was associated with glycaemic control as found in three studies [40, 61, 62]. All exercise programs were associated with improved glycaemic control as found in four studies [37, 38, 52, 76, 84]. Adding a second OHA was associated with poor glycaemic control in one study [49]. The effectiveness of a community-based multilevel peer support intervention was associated with a significant reduction in glycosylated haemoglobin in the intervention group in one study [24].

## Discussion

We sought to determine the prevalence and factors associated with glycaemic control in sub-Saharan Africa (SSA) in the past 10 years (2012–2022). Our review shows that poor glycaemic control is common in SSA with only 10–60% of patients having optimal glycaemic control. In addition, glycaemic control was associated with sociodemographic factors (younger and older age, gender, lower income, absence of health insurance, low level of education, place of residence, family support, coping strategies), lifestyle (dietary adherence, practice of exercise, smoking, alcohol consumption), clinical factors (family history of diabetes, longer duration of diabetes, presence of comorbidities/complications), adherence (adhering to follow-up appointments and medication), treatment modalities (pill burden, treatment regimen, use of statins or anti-hypertensives, drug-related problems, diabetes information from non-health workers, high diabetes health literacy, absence of clarity in pharmacists’ advice, failure to set glycaemic goals), and reported glycaemic control optimization interventions.

The assessment of glycaemic control was variable in the studies included in our review; in only 43 (58.1%) studies, glycosylated haemoglobin was used. This renders it difficult to estimate the real extent of glycaemic control, compare the results and, even in daily clinical practice, manage patients [90]. Nevertheless, our estimated prevalence of good glycaemic control in SSA is similar to the prevalence found by a meta-analysis in Ethiopia in which only one-third of patients were adequately controlled [90] and in a study in Central, East and West Africa with approximately 29% of good glycaemic control [91]. The prevalence of poor glycaemic control in sub-Saharan Africa is far lower than that found in eight European countries by The Panorama study (62.6%) [10], and in the United States of America by Fang and colleagues (55.3%) in 2015–2018 [92]. The poor glycaemic control in sub-Saharan Africa is the result of poor quality of diabetes care due, in turn, to a weak disease management framework and fragmented health systems [93]. Changes are required in the organization of healthcare systems in sub-Saharan African countries for better management of non-communicable diseases in general, with effective implementation of diabetes care into primary care [4, 93].

Several studies have reported significant associations between sociodemographic factors and glycaemic control. Advancing in age was negatively associated with poor glycaemic control, indicating the vulnerability of young patients as found by several studies [93]. Young patients are confronted with many barriers to effective self-management. Older age was associated with poor glycaemic control in our review, corroborating the findings of several studies and explained by insulin resistance and the presence of comorbidities [8, 90, 95]. Although both genders were linked to poor and good glycaemic control in our review, it is recognized that women are traditionally prone to poor glycaemic control [96]. Women with type 2 diabetes in sub-Saharan Africa have a greater risk of death due to poor access to care [97]. Thus, young, and older patients along with women represented vulnerable categories, in terms of propensity to poor glycaemic control and issues of accessing care. Caution must be taken when managing diabetes in sub-Saharan Africa to ease access to care and provide adequate responses to the needs of these categories.

Poor socioeconomic conditions (low income, poor education) have been associated with poor glycaemic control due to poor access to adequate care and poor health-seeking behaviors [18, 48, 87, 98, 99]. Increasing universal health coverage could address these problems and lead to better outcomes [100]. Factors such as food insecurity and depression have been identified as mediators in the relationship between poor living conditions and glycaemic control [98]*.* Family support and adequate coping strategies such as participation in religious activities were beneficial for glycaemic control and could act through these mediating factors. Management interventions to optimize glycaemic control for patients with type 2 diabetes with poor socioeconomic conditions should consider these interconnected factors.

A long distance from home to the healthcare facility has been associated with poor glycaemic control while having less distance was found to be beneficial in many studies, as the latter favors access, adherence and monitoring of care [101, 102]. However, for the nearness of health facilities to have a meaningful impact, these facilities must have adequate equipment, and trained personnel for diabetes care [32].

As expected, adherence to dietary recommendations and physical exercise have been associated with good glycaemic control [103, 104]. In our review smoking was associated with poor glycaemic control. The literature shows that smoking has a confusing relationship with poor glycaemic control [104]. Indeed, if smoking was related to poor glycaemic control due to reduced effectiveness of insulin, quitting smoking has also been linked to poorer glycaemic control [106, 107]. Nevertheless, smoking cessation is one goal of diabetes care. One study in our review linked patients who ever drunk alcohol regularly to poor glycaemic control [29], and the author did not provide details on the quantity used and the term. The literature showed that drinking moderately in the short or medium-term did not affect glycaemic control [108]. Current guidelines support moderate alcohol consumption as excessive chronic alcohol consumption or acute intoxication that adversely has detrimental effects on all organs and affects mortality and morbidity [109, 110]. In sub-Saharan Africa, careful recommendations on alcohol use need to be developed for patients with type 2 diabetes as alcohol might represent a concurrent source of expenses of the few resources available. The real nature of alcoholic beverages found in sub-Saharan Africa is not accurately known.

Several studies confirmed our findings concerning clinical factors with respect to glycaemic control. With a longer duration of diabetes, there is a deterioration of the function of the pancreas due to failure in beta cells, and the emergence of disease-related complications, which in turn can have effects on glycaemic control [8, 111]. The presence of comorbidities/complications poses a problem with respect to pill burden, adherence to treatment and cost, or as an intricate mechanism linked to beta-cell impairment or aggravation of insulin resistance [8, 94, 112–117]. In sub-Saharan Africa, there is a high proportion of undiagnosed diabetes mellitus, and the diagnosis is often delayed. At diagnosis, many patients will present with complications or comorbidities, thus complicating the management and the attainment of glycaemic targets [118]. Strategies to improve the diagnosis of diabetes mellitus must be considered by policy makers in sub-Saharan Africa for a reduction in diabetes-related complications and mortality [118].

Good medication adherence was associated with good glycaemic control. This finding is in line with that found in several studies [111, 119, 120], particularly that good adherence improves glycaemic control, leads to fewer emergency department visits, decreases hospitalizations, and lowers medical costs [121]. In sub-Saharan Africa, medication adherence is confronted by the issues of access and affordability of drugs [122]. The organization of a regular and reliable system for the supply of medicines at affordable prices, even in remote areas is essential to improve diabetes care.

Oral hypoglycaemic agents (OHAs), either insulin only or combined with the former, were associated with poor glycaemic control in our review. In the included studies, the matters surrounding medication use, such as adherence, reason for prescribing one agent or a combination, were not reported. The use of statins and some antihypertensive agents (thiazide diuretics and non-selective beta-blockers) are linked to comorbidities, with hypertension being the most frequent [123] and having been linked in several studies with high levels of glycosylated haemoglobin [124–126]. Since many patients have comorbidities, present late and may need these adjunct treatments, these findings have implications for the management of patients with type 2 diabetes in sub-Saharan Africa. They call for the judicious use of these agents and adherence of healthcare professionals to evidence-based diabetes care in SSA.

Concerning the reported interventions, all the exercise programs, and one educational program for self-management were associated with good glycaemic control. Nevertheless, the full integration of exercise into routine healthcare in Africa is challenged by poor knowledge and attitudes of patients and healthcare providers [127]. In the same way, self-management of diabetes is poor in Africa as it faces numerous barriers [128, 129]. Peer-support interventions have been increasingly recognized worldwide, but one may note that the transferability of interventions across different cultures might be difficult [24]. Research is needed to identify effective interventions to optimize glycaemic control in the context of sub-Saharan Africa.

To the best of our knowledge, this systematic review is the first to provide a prevalence estimate of glycaemic control and an overview of factors associated with glycaemic control in patients with type 2 diabetes in SSA. The review only considered studies in which multivariate analysis was performed in the data analysis and therefore excluded factors without an uncertain link to glycaemic control. The findings of this systematic review are also as good as the quality of the studies included, more so that most (70.2%) were of moderate quality. Most of the studies were observational and one cannot ascertain causality between factors identified and glycaemic control. Glycaemic control was assessed using different methods across the studies, with only 58.1% of the included studies using the recommended glycosylated haemoglobin. There were also different thresholds for glycaemic control through studies even if the same glycaemic control assessment was used. These variations in assessment standards have the potential for errors in estimates and misclassifications.

Beyond these limitations, this systematic review is, to our knowledge, the first to provide a broad view of the extent and multifactorial drivers of glycaemic control among patients with type 2 diabetes in SSA. The review highlights the need for changes in the organization of the healthcare systems in sub-Saharan Africa while ensuring effective funding. Health providers must be trained, and health facilities equipped for adequate diabetes care. The screening of diabetes mellitus must be improved as well as access to care for vulnerable patients. While this review highlights the need for multipronged interventions to improve glycaemic control and diabetes care in this region, further studies are needed to assess their feasibility, effectiveness, affordability and acceptability.

## Conclusion

Suboptimal glycaemic control is pervasive among patients with type-2 diabetes in sub-Saharan Africa and poses a significant public health challenge. While urgent interventions are required to optimize glycaemic control in this region, these should consider sociodemographic, lifestyle, clinical, and treatment-related factors.

## Supplementary Information

**Additional file 1: Table S1.** Search strategy in the included databases. A description of the strategy used for the literature search.

**Additional file 2: Table S2.** General characteristics of excluded studies and reason for exclusion. A description of the excluded studies and the reason for their exclusion.

**Additional file 3: Table S3.** Data for metanalysis of proportions of glycaemic control in included studies. Data extracted for metaanalysis in the individual studies.

**Additional file 4: Table S4.** Assessment of methodological quality for included cross-sectional studies. Assessment of the risk of bias for cross-sectional studies with the Joanna Briggs checklist.

**Additional file 5: Table S5.** Assessment of methodological quality of quasi-experimental studies. Assessment of the risk of bias for quasi-experimental studies with the Joanna Briggs checklist.

**Additional file 6: Table S6.** Assessment of methodological quality for included randomized controlled trials. Assessment of the risk of bias for randomized controlled trials

**Additional file 7: Table S7.** Assessment of methodological quality of case–control studies. The cross-sectional studies assessed with the Joanna Briggs checklist.

**Additional file 8: Table S8.** Assessment of methodological quality of cohort study. Assessment of the risk of bias for cohort study through the Joanna Briggs checklist.

**Additional file 9. **List of references of excluded studies. The list of excluded references

## Data Availability

The dataset(s) supporting the conclusions of this article is(are) included within the article and in the supplementary files. The protocol can be accessed from request to the corresponding author.

## Abbreviations

BMI: Body mass index
EGFR: Estimated Glomerular Filtration Rate
FBG: Fasting blood glucose
FBS: Fasting blood sugar
FPG: Fasting plasma glucose
Ghb: : Glycosylated haemoglobin
GLUT2: Glucose transporter 2
HbA1c: Haemoglobin A1c
JMF: Joel Msafiri Francis
JPF: Jean-Pierre FINA
LDL-C: Low-Density Lipoprotein-Cholesterol
Mh: : MesH term
Niddm: : Non insulin dependent diabetes mellitus
OBO: Olufemi Babatunde Omole
OHA: Oral Hypoglycaemic Agents
PRISMA: Preferred Reporting Items for Systematic Reviews and Meta-analyses
SSA: Sub-Saharan Africa
T2d: Type 2 diabetes mellitus
Tw: Text word
WHO: World Health Organization
