# Supplementary material for: Glycaemic control among type 2 diabetes patients in sub-Saharan Africa from 2012 to 2022: a systematic review and meta-analysis
Source: Diabetol Metab Syndr. 2022 Sep 20;14:134. doi: 10.1186/s13098-022-00902-0 (PMC9487067; doi:10.1186/s13098-022-00902-0)
Supplement: Supplementary file 1 — Additional file 1: Table S1. Search strategy in the included databases. A description of the strategy used for the literature search. [file 13098_2022_902_MOESM1_ESM.docx]

**Additional file 1: Table S**1. Search strategy in included databases

|  | **Search strategy** |
| --- | --- |
| African Index Medicus | tw:(tw:((tw:(glycemic control)) OR (tw:(glycaemic control)) OR (tw:(blood glucose control)) AND (tw:(type 2 diabetes)) OR (tw:(type ii diabetes mellitus)) OR (tw:(diabetes mellitus, type 2))) AND (year_cluster:[2011 TO 2022])) |
| Africa-Wide Information | (glycaemic control OR glycemic control or hba1c OR ghb OR blood sugar OR blood glucose OR glycated haemoglobin OR haemoglobin a1c) AND (type 2 diabetes OR type 2 diabetes mellitus OR t2dm OR t2d OR niddm OR non-insulin dependent diabetes mellitus) AND Africa  Limits: 2011-2022 |
| Global Health | (glycaemic control OR glycemic control OR hba1c OR ghb OR blood sugar OR blood glucose OR glycated hemoglobin OR haemoglobin a1c) AND (type 2 diabetes OR type 2 diabetes mellitus OR t2dm OR t2d OR niddm OR non-insulin dependent diabetes mellitus) AND Africa  Limits: January 2011-February 2022 |
| PubMed | (((((((((((((((((((((Type 2 diabetes mellitus [tw]) OR (Type II diabetes mellitus [tw])) OR (Non insulin dependent diabetes mellitus [tw])) OR (Ketosis resitant diabetes mellitus [tw])) OR (Type 2 diabetes [tw])) OR (Type II diabetes [tw])) OR (Type 2 diabetes mellitus [mh])) AND (Cause [tw])) OR (Etiology [tw])) OR (Determinant [tw])) OR (Factor [tw])) OR (Contributor [tw])) OR (Predictor [tw])) OR (Risk factor [tw])) OR (Risk factor [mh])) AND (Glycaemic control [tw])) OR (Blood glucose control [tw])) OR (Glycemic control [tw])) OR (Glycemic control [mh])) AND (sub Saharan africa [tw])) OR (Africa south of the sahara [tw])) OR (Africa south of the sahara [mh])  Filters applied: Clinical Trial, Observational Study, Randomized Controlled Trial, from 2011/1/1-2022/2/28, Female, Male, Adult: 19+ years. |
| Web of Science | glycaemic OR glycemic OR blood OR glucose AND control AND type 2 diabetes OR non insulin dependent diabetes OR ketosis resistant diabetes OR type II diabetes AND africa south of the sahara OR sub saharan africa (All Fields) and 2022 or 2021 or 2020 or 2019 or 2018 or 2017 or 2016 or 2015 or 2014 or 2013 or 2012 or 2011 (Publication Years) and Articles (Document Types) and ETHIOPIA or UGANDA or KENYA or GHANA or TANZANIA or CAMEROON or MALAWI or SUDAN or ZAMBIA or ZIMBABWE or RWANDA or MOZAMBIQUE or DEM REP CONGO or BOTSWANA or BURKINA FASO or GAMBIA or BENIN or COTE IVOIRE or SENEGAL or MALI or REP CONGO or GABON or NAMIBIA or TOGO or NIGER or LIBERIA or ESWATINI or MADAGASCAR or GUINEA or LESOTHO or ANGOLA or ERITREA or BURUNDI or CHAD or GUINEA BISSAU or SWAZILAND or CENT AFR REPUBL or SOMALIA or SOUTH SUDAN or SOUTH AFRICA or NIGERIA (Countries/Regions) and Endocrinology Metabolism or Health Care Sciences Services (Web of Science Categories) |

Legend: tw: text word, DM: diabetes mellitus, t2d: type 2 diabetes mellitus, niddm: non insulin dependent diabetes mellitus, hba1c: haemoglobin glycosylated, ghb: glycosylated haemoglobin, mh: MesH term
