# Supplementary material for: Glycaemic control among type 2 diabetes patients in sub-Saharan Africa from 2012 to 2022: a systematic review and meta-analysis
Source: Diabetol Metab Syndr. 2022 Sep 20;14:134. doi: 10.1186/s13098-022-00902-0 (PMC9487067; doi:10.1186/s13098-022-00902-0)
Supplement: Supplementary file 2 — Additional file 2: Table S2. General characteristics of excluded studies and reason for exclusion. A description of the excluded studies and the reason for their exclusion. [file 13098_2022_902_MOESM2_ESM.docx]

**Additional file 2: Table S2.** General characteristics of excluded studies and reason for exclusion

| **First author last name** | **Study type** | **Publication  year** | **Study setting** | **Study Population** | **Sample size** | **Reason for exclusion** |
| --- | --- | --- | --- | --- | --- | --- |
| Abubakar [1] | Retrospective case-control study | 2014 | Nigeria | Type 2 diabetes; 30-70 years; treatment failure with metformin and glibenclamide | 520 | Ineligible data analysis |
| Abutair [2] | Randomized controlled trial | 2018 | Palestine | Type 2 diabetes | 40 | Setting outside sub-Saharan Africa |
| Adeniyi [3] | Experimental  study | 2013 | Nigeria | Type 2 diabetes | 29 | Another outcome: influence of gender on time for glycaemic improvement |
| Adisa [4] | Cross-sectional | 2017 | Nigeria | Hypertension, type 2 diabetes, and Type 2 diabetes comorbid with hypertension | 450 | Ineligible data analysis |
| Adisa [5] | Cross-sectional | 2016 | Nigeria | Type 2 diabetes; 51-70 years | 176 | Ineligible data analysis |
| Adisa [6] | Prospective cross-sectional and retrospective Review | 2013 | Nigeria | Type 2 diabetes | 176 | Ineligible data analysis |
| Aga [7] | Cross-sectional, correlational study | 2020 | USA | Adult patients with comorbid heart failure and Type 2 diabetes | 180 | Setting outside sub-Saharan Africa |
| Amendezo [8] | Randomized Controlled Trial | 2017 | Rwanda | Types 1 and 2 diabetes | 223 | Another type of diabetes included |
| Anakwue [9] | Cross-sectional | 2019 | Nigeria | Types 1 and 2 diabetes | 84 | Another type of diabetes included |
| Anetor [10] | Case-control | 2016 | Nigeria | Type 2 diabetes and healthy controls | 65 | Ineligible data analysis |
| Angamo [11] | Cross-sectional | 2013 | Ethiopia | Types 1 and 2 diabetes | 284 | Another type of diabetes included |
| Anyakudo [12] | Experimental controlled study | 2014 | Nigeria | Male Type 2 diabetes | 20 | Another outcome: postprandial glycemic index of food processing methods |
| Asfaw [13] | Cross-sectional | 2014 | Ethiopia | Type 2 diabetes | 103 | Ineligible data analysis |
| Awadalla [14] | Cross-sectional | 2017 | Sudan | Type 2 diabetes | 424 | Ineligible data analysis |
| Awadalla [15] | Cross-sectional | 2018 | Sudan | Type 2 diabetes; 20-75 years | 188 | Ineligible data analysis |
| Awodele [16] | Descriptive and prospective study | 2015 | Nigeria | Type 2 diabetes; 18 years | 152 | Another outcome: medication adherence |
| Azar [17] | Randomized, parallel, open-label, active-controlled trial | 2016 | Algeria, Israel, India, Lebanon, Malaysia, South Africa, and United Arab Emirates | Type 2 diabetes; 18-80 years | 343 | An international study with sites outside sub-Saharan Africa |
| Azenabor [18] | Cross-sectional | 2011 | Nigeria | Type 2 diabetes; 40-60 years | 300 | Ineligible data analysis |
| Azubike [19] | Cohort study | 2013 | Nigeria | Type 2 diabetes with diabetic nephropathy | 22 | Another outcome: progression glycaemia in diabetic nephropathy |
| Bello [20] | Cross-sectional | 2019 | Nigeria | Types 1 and 2 diabetes | 175 | Another type of diabetes included |
| Biadgo [21] | Cross-sectional | 2016 | Ethiopia | Type 2 diabetes and healthy controls | 296 | Ineligible data analysis |
| Botha [22] | Cohort study | 2017 | Scotland | Type 2 diabetes | 23,208 | Setting outside Sub-Saharan Africa |
| Bulbulia [23] | Retrospective cross sectional clinical audit | 2020 | South Africa | Type 2 diabetes on either insulin only therapy or insulin and metformin therapy; 30-88 years | 321 | Ineligible data analysis |
| Correia [24] | Audit | 2020 | Guinea-Bissau | Types 1 and 2 diabetes | 63 | Another type of diabetes included |
| Dalia [25] | Cross-sectional and case-control | 2019 | Sudan | Type 2 diabetes and healthy controls | 120 | Ineligible data analysis |
| Darko [26] | Nested case-control | 2015 | Ghana | Type 2 diabetes and controls; 25-70 years | 189 | Another outcome: variations in the levels of InterLeukin-6 and Tumor Necrosis Factor-α in patients with type 2 diabetes |
| Danquah [27] | Cross-sectional | 2012 | Ghana | Type 2 diabetes | 675 | Another outcome: prevalence of diabetes |
| Diaf [28] |  | 2015 | Libya | Type 2 diabetes | 238 | Setting outside sub-Saharan Africa |
| Dickie [29] | Cohort study | 2014 | South Africa | Healthy women without diabetes | 297 | Study population other than patients with diabetes |
| Diouf [30] | Cross-sectional | 2015 | Senegal | Type 2 diabetes; ≥33 years | 195 | Ineligible data analysis |
| Diouf [31] | Retrospective study | 2013 | Senegal | Type 2 diabetes; 36-83 years | 130 | Ineligible data analysis |
| Distiller [32] | Cross-sectional | 2016 | South Africa | Types 1 and 2 diabetes | 50 | Another type of diabetes included; another outcome |
| Edo [33] | Randomized trial | 2011 | Nigeria | Type 2 diabetes | 10 | Another outcome: plasma glucose response |
| Elkhidir [34] | Case-control | 2017 | Sudan | Type 2 diabetes  and healthy controls; 44-60 years | 87 | Ineligible data analysis |
| Essien [35] | Randomized controlled trial | 2017 | Nigeria | Types 1 and 2 diabetes | 104 | Another type of diabetes included |
| Ewenighi [36] | 12-week diabetic  management therapy | 2013 | Nigeria | Type 2 diabetes; ≥18 years | 52 | Another outcome: responses to glycemic control therapy according to age, gender, level of adiposity, and duration of diabetes |
| Ezenwaka [37] | Cross-sectional | 2014 | Nigeria | Type 2 diabetes; 40-84 years | 89 | Ineligible data analysis |
| Farmer [38] | Protocol study | 2019 | South Africa and Malawi | Type 2 diabetes |  | Study type: study protocol |
| Fondjo [39] | Cross-sectional | 2018 | Ghana | Type 2 diabetes, women; ≥25 years | 192 | Ineligible data analysis |
| Geneto [40] | Comparative Cross-sectional | 2015 | Ethiopia | Type 2 diabetes and healthy controls; 20-76 years | 108 | Ineligible data analysis |
| Gill [41] | Retrospective study | 2012 | South Africa | Type 2 diabetes | 120 | Another outcome: effect of the introduction of a standard monitoring protocol on the investigations performed on the metabolic control of type 2 diabetes |
| Hall [42] | Cross-sectional | 2017 | Cameroon | Type 2 diabetes | 261 | Ineligible data analysis |
| Idonije [43] | Case-control | 2011 | Nigeria | Type 2 diabetes | 130 | Another outcome: comparison of plasma glucose level, creatinine, and urea among diabetic and healthy patients |
| Iloh [44] | Descriptive study | 2017 | Nigeria | Type 2 diabetes; ≥18 years | 120 | Ineligible data analysis |
| Iloh [45] | Cross-sectional | 2018 | Nigeria | Type 2 diabetes | 120 | Ineligible data analysis |
| Iwuala [46] | Cross-sectional | 2015 | Nigeria | Type 2 diabetes | 100 | Ineligible data analysis |
| Jackson [47] | Cross-sectional | 2021 | Nigeria | Type 2 diabetes | 226 | Ineligible data analysis |
| Jemal [48] | Cross-sectional | 2017 | Ethiopia | Type 2 diabetes; ≥18 years | 196 | Ineligible data analysis |
| Karau [49] | Cross-sectional | 2019 | Kenya | Type 2 diabetes; >18 years | 151 | Ineligible data analysis |
| Leulseged [50] | Retrospective chart Review | 2019 | Ethiopia | Type 2 diabetes; >18 years | 686 | Another outcome: time to optimal glycaemic control and associated factors |
| Madela [51] | Cross-sectional | 2020 | South Africa | General Population | 6481 | Another outcome: screening  general population for  hypertension and diabetes |
| Makan [52] | prospective study | 2013 | South Africa | Type 2 diabetes | 14 | Study type: conference proceeding |
| Mashitisho [53] | Review | 2016 |  | Type 2 diabetes |  | Study type: a clinical review |
| Matheka [54] | Cross-sectional | 2013 | Kenya | Types 1 and 2 diabetes | 198 | Another type of diabetes included; another outcome: use and level of glycosylated haemoglobin (HbA1c) |
| Matshipi [55] | Longitudinal study | 2017 | South Africa | General Population | 713 | Other population |
| Mbwete [56] | Cross-sectional | 2020 | Tanzania | Type 2 diabetes and hypertension; >18 years | 161 | Ineligible data analysis |
| Mbouemboue [57] | Cross-sectional | 2018 | Cameroon | General Population | 948 | Other population; another outcome: prevalence of diabetes in a community setting |
| Mels [58] | Cohort study | 2013 | South Africa | General Population | 409 | Other Population |
| Mogre [59] | Psychometric evaluation of the summary of diabetes self-care activities | 2019 | Ghana | Type 2 diabetes | 187 | Another outcome: Psychometric evaluation of the summary of diabetes self-care activities |
| Mohamed [60] | Intervention study | 2012 | Egypt | Type 2 diabetes and normal subjects | 16 | Setting outside sub-Saharan Africa |
| Monanabela [61] | Retrospective,  descriptive study | 2019 | South Africa | Type 2 diabetes | 575 | Another outcome: HbA1c and fasting plasma glucose results in guiding treatment changes |
| Motta [62] | Cohort study | 2017 | South Africa | Types 1 and 2 diabetes |  | Another type of diabetes included |
| Moustafa [63] | Randomized controlled trial | 2019 | Egypt | Type 2 diabetes |  | Setting outside sub-Saharan Africa |
| Muddu [64] | Cross-sectional | 2018 | Uganda | Types 1 and 2 diabetes | 201 | Another type of diabetes included |
| Mullugeta [65] | Cross-sectional | 2012 | Ethiopia | Type 2 diabetes | 165 | Ineligible data analysis |
| Munyogwa [66] | Cross-sectional | 2020 | Tanzania | Type 2 diabetes; ≥18 years | 330 | Ineligible data analysis |
| Musenge [67] | Cross-sectional | 2016 | Zambia | Types 1 and 2 diabetes | 198 | Another type of diabetes included |
| Neboh [68] |  | 2012 | Nigeria | Type 2 diabetes | 130 | Another outcome: comparison of type 2 diabetic patients and healthy individuals |
| Nduati [69] | Cross-sectional | 2016 | Kenya | Type 2 diabetes; ≥35 years | 149 | Ineligible data analysis |
| Ngala [70] | Case-control | 2014 | Ghana | Type 2 diabetes | 200 | Another outcome: effect on diabetes |
| Ng'ang'a [71] | study protocol | 2020 | Rwanda | Insulin-dependent Type 2 diabetes | 82 | Study type: study protocol |
| Nganou-Gnindjio [72] | Cross-sectional | 2018 | Cameroon | Type 2 diabetes; 43-62 years | 54 | Ineligible data analysis |
| Nielsen [73] | Comparative Cross-sectional | 2015 | Uganda | People living in diabetic households | 90 | Other Population |
| Nkoana [74] | Prospective  observational study | 2020 | South Africa | Types 1 and 2 diabetes | 96 | Another outcome: relationship between self-monitored fasting blood glucose (FBG) levels and HbA1c values |
| Odume [75] | Descriptive Cross-sectional | 2015 | Nigeria | Type 2 diabetes;18-70 years | 145 | Ineligible data analysis |
| Ofori [76] | Cross-sectional | 2019 | Ghana | Type 2 diabetes and healthy controls | 161 | Ineligible data analysis |
| Okoro [77] | Cross-sectional | 2019 | Nigeria | Type 2 diabetes  and healthy health workers (controls); 35-75 years | 180 | Ineligible data analysis |
| Olaniyan [78] | Descriptive study | 2019 | Nigeria | Type 2 diabetes; ≥18 years | 189 | Ineligible data analysis |
| Omar [79] | Cross-sectional | 2019 | Sudan | Type 2 diabetes | 600 | Another outcome than glycaemic control |
| Osei-Yeboah [80] | Cross-sectional | 2019 | Ghana | Type 2 diabetes; 27-84 years | 150 | Ineligible data analysis |
| Osei-Yeboah [81] | Cross-sectional | 2018 | Ghana | Healthcare workers | 112 | Other Population |
| Osman [82] | Descriptive study | 2013 | Sudan | Types 1 and 2 diabetes | 400 | Another type of diabetes included |
| Owolabi [83] | Randomized controlled trial | 2019 | South Africa | Adult with uncontrolled diabetes; types 1 and 2 diabetes | 216 | Another outcome: Efficacy, acceptability, and feasibility of daily text-messaging  in promoting glycaemic control and Other clinical outcomes; another type of diabetes included |
| Paruk [84] | Retrospective observational study | 2017 | South Africa | Type 2 diabetes |  | Another outcome: patterns of treatment and control |
| Pengpid [85] | Study protocol | 2014 | South Africa | General population | 300 | Study type: study protocol; other population |
| Philis-Tsimikas [86] | 26-week, open-label trial | 2013 | Multicentre study | Type 2 diabetes uncontrolled on oral antidiabetic agents | 458 | Other Setting |
| Pillay [87] | Cross-sectional | 2018 | South Africa | Types 1 and 2 diabetes | 744 | Another type of diabetes included |
| Pinchevsky [88] | Descriptive Cross-sectional | 2013 | South Africa | Retrospective | 666 | Another outcome: identifying treatment gaps in the management of type 2 diabetes after implementation of guidelines |
| Pinchevsky [89] | Cross-sectional | 2015 | South Africa | Type 2 diabetes; ≥18 years | 261 | Ineligible data analysis |
| Pinchevsky [90] | Retrospective study | 2016 | South Africa | Type 2 diabetes; ≥18 years | 261 | Ineligible data analysis |
| Pinchevsky [91] | Cross-sectional | 2017 | South Africa | Type 2 diabetes; ≥18 years | 519 | Another outcome: treatment gaps in the management of type 2 diabetes |
| Pirie [92] | Cross-sectional | 2014 | South Africa | Type 2 diabetes | 292 | Another outcome: a predictor of diabetic retinopathy |
| Ramkisson [93] | Cross-sectional | 2017 | South Africa | Type 2 diabetes; ≥18 years | 401 | Ineligible data analysis |
| Rwegerera [94] | Cross-sectional | 2021 | Botswana | Type 2 diabetes with and without HIV infection | 357 | Ineligible data analysis |
| Sadik [95] | Quantitively exploratory descriptive  research study | 2017 | Sudan | Type 2 diabetes with manifestations suggestive of  acute myocardial  ischemia and healthy controls | 140 | Ineligible data analysis |
| Shimels [96] | Cross-sectional | 2021 | Ethiopia | Diabetic hypertensive patients | 409 | Another outcome: adherence to medication, glycaemic control not assessed |
| Randeree [97] | Prospective, open-label,  non-interventional, observational,  24-week study | 2013 | International study | Type 2 diabetes | 2,026 | Setting outside Sub-Saharan Africa |
| Segal [98] | Non-Randomized interventional study | 2012 | South Africa | Types 1 and 2 diabetes | 77 | Another type of diabetes included |
| Sobngwi [99] | Cross-sectional | 2012 | East Africa (Tanzania and Kenya), Central Africa (Cameroon) and West Africa (Ghana, Senegal, and Nigeria) | Type 2 diabetes | 2352 | Another outcome: factors of glycaemic control not assessed |
| Tayo [100] | Cross-sectional | 2017 | South Africa | Types 1 and 2 diabetes | 97 | Another type of diabetes included |
| Udoh [101] | Cross-sectional | 2019 | Nigeria | Type 2 diabetes; ≥40 years | 208 | Ineligible data analysis |
| Unung [102] | Case-control | 2020 | Nigeria | Type 2 diabetes with or without Diabetic retinopathy; non-diabetic normohypertensive control; 30-80 years | 150 | Ineligible data analysis |
| van den Berg [103] | Cross-sectional | 2019 | Lesotho | Type 2 diabetes | 122 | Another outcome: a descriptive study, factors of glycaemic control not assessed |
| Woyesa [104] | Cross-sectional | 2017 | Ethiopia | Type 2 diabetes mellitus; ≥30-80 years | 319 | Ineligible data analysis |
| Yameogo [105] | Cross-sectional | 2012 | Senegal | Type 2 diabetes; 40-85 years | 318 | Ineligible data analysis |
