# Supplementary material for: Glycaemic control among type 2 diabetes patients in sub-Saharan Africa from 2012 to 2022: a systematic review and meta-analysis
Source: Diabetol Metab Syndr. 2022 Sep 20;14:134. doi: 10.1186/s13098-022-00902-0 (PMC9487067; doi:10.1186/s13098-022-00902-0)
Supplement: Supplementary file 3 — Additional file 3: Table S3. Data for metanalysis of proportions of glycaemic control in included studies. Data extracted for metaanalysis in the individual studies. [file 13098_2022_902_MOESM3_ESM.docx]

**Additional file 3: Table S3.** Data for metaanalysis of proportions of glycaemic control in included studies

| **Study** | **n** | **N** | **study population** | **method** | **Country** | **Type** | **Region** |
| --- | --- | --- | --- | --- | --- | --- | --- |
| Achila 2020 | 72 | 309 | Adults with type 2 diabetes | hba1c | Eritrea | cross-sectional | Eastern |
| Abebe 2022 | 35 | 138 | Adults with type 2 diabetes | fbg | Ethiopia | cross-sectional | Eastern |
| Abera 2022 | 85 | 325 | Adults with type 2 diabetes | hba1c | Ethiopia | cross-sectional | Eastern |
| Adejumo 2012 | 64 | 144 | Adults with type 2 diabetes and normal renal function | hba1c | Nigeria | cross-sectional | Western |
| Adeniyi 2016 | 53 | 327 | ≥ 30 years of age and type 2 diabetes | hba1c | South Africa | cross-sectional | Southern |
| Afolabi 2018 | 29 | 80 | 40-80 years of age and type 2 diabetes | hba1c | Nigeria | cross-sectional | Western |
| Akabwai 2016 | 75 | 279 | Adults with type 2 diabetes | hba1c | Uganda | cross-sectional | Central |
| Anioke 2019 | 23 | 138 | ≥ 30 years of age and type 2 diabetes | hba1c | Nigeria | cross-sectional | Western |
| Ayele 2019 | 118 | 275 | Adults with type 2 diabetes | fbg | Ethiopia | cross-sectional | Eastern |
| Belay 2017 | 62 | 188 | Adults with type 2 diabetes | fbg | Ethiopia | cross-sectional | Eastern |
| BeLue 2016 | 26 | 106 | Adults with type 2 diabetes | hba1c | Senegal | cross-sectional | Western |
| Biadgo 2018 | 65 | 159 | Adults with type 2 diabetes | fbg | Ethiopia | cross-sectional | Eastern |
| Biru 2017 | 41 | 322 | Adults with type 2 diabetes | fbg | Ethiopia | cross-sectional | Eastern |
| Blum 2019 | 45 | 319 | Adults with type 2 diabetes | hba1c | DR Congo | cross-sectional | Central |
| Botchway 2021 | 58 | 234 | Adults with type 2 diabetes | hba1c | Ghana | cross-sectional | Western |
| Camara 2014 | 328 | 1267 | ≥16 years type 2 diabetes | hba1c | Cameroon and Guinea | cross-sectional | Central |
| Dagnew 2017 | 33 | 105 | ≥30 years type 2 diabetes | fbg | Ethiopia | cross-sectional | Eastern |
| Demoz 2019 | 113 | 357 | Adults with type 2 diabetes | hba1c | Ethiopia | cross-sectional | Eastern |
| Eticha 2016 | 197 | 384 | Adults with type 2 diabetes | hba1c | Ethiopia | cross-sectional | Eastern |
| Fekadu 2019 | 80 | 228 | adult type diabetes | fbg | Ethiopia | cross-sectional | Eastern |
| Fseha 2017 | 73 | 200 | Adults with type 2 diabetes | fbg | Ethiopia | cross-sectional | Eastern |
| Id 2018 | 178 | 394 | Adults with type 2 diabetes | fbg | Ethiopia | cross-sectional | Eastern |
| Inih 2018 | 52 | 108 | adult males  with type 2 diabetes | hba1c | Nigeria | cross-sectional | Western |
| Kalain 2020 | 49 | 200 | Adults with type 2 diabetes | hba1c | South Africa | cross-sectional | Southern |
| Kamuhabwa 2014 | 142 | 469 | Adults with type 2 diabetes | fbg | Tanzania | cross-sectional | Eastern |
| Kassahun 2016 | 90 | 309 | Adults with type 2 diabetes | fbg | Ethiopia | cross-sectional | Eastern |
| Khoza 2018 | 71 | 320 | >30 years age and type 2 diabetes | hba1c | South Africa | case-control | Southern |
| Kimando 2017 | 152 | 385 | ≥30 years age and type 2 diabetes | hba1c | Kenya | cross-sectional | Eastern |
| Mash 2014 | 275 | 1570 | Adults with type 2 diabetes | hba1c | South Africa | Pragmatic  randomized  controlled trial | Southern |
| Mashele 2019 | 27 | 176 | Adults with type 2 diabetes | hba1c | South Africa | cross-sectional | Southern |
| Mobula 2018 | 368 | 1226 | Adults with type 2 diabetes | hba1c | Ghana | cross-sectional | Western |
| Mohamed 2013 | 137 | 457 | Adults with type 2 diabetes, and non-diabetic controls | hba1c | Sudan | case-control | Central |
| Mohammed 2020 | 115 | 307 | Adults with type 2 diabetes | fbg | Ethiopia | cross-sectional | Eastern |
| Mphwanthe 2020 | 170 | 428 | ≥25 years age and type 2 diabetes | hba1c | Malawi | cross-sectional | Southern |
| Mwavua 2016 | 34 | 200 | Adults with type 2 diabetes | hba1c | Kenya | cross-sectional | Eastern |
| Mwita 2019 | 159 | 500 | Adults with type 2 diabetes | hba1c | Botswana | cross-sectional | Southern |
| Noor 2017 | 59 | 387 | Adults with type 2 diabetes | hba1c | Sudan | cross-sectional | Central |
| Omar 2018 | 96 | 339 | Adults with type 2 diabetes | hba1c | Sudan | cross-sectional | Central |
| Osuji 2018 | 127 | 316 | Adults with type 2 diabetes | hba1c | Nigeria | cross-sectional | Western |
| Otieno 2017 | 67 | 220 | ≥30 years age and type 2 diabetes | hba1c | Kenya | cross-sectional | Eastern |
| Oyewole 2019 | 21 | 70 | ≥21 years age and type 2 diabetes | fbg | Nigeria | cross-sectional | Western |
| Rwegerera 2019 | 136 | 368 | Adults with type 2 diabetes | hba1c | Botswana | cross-sectional | Southern |
| Shimels 2018 | 142 | 414 | Adults with type 2 diabetes | fbg | Ethiopia | cross-sectional | Eastern |
| Tefera 2020 | 72 | 400 | Adults with type 2 diabetes | fbg | Ethiopia | cross-sectional | Eastern |
| Tekalegn 2018 | 83 | 412 | ≥15 years age and type 2 diabetes | fbg | Ethiopia | cross-sectional | Eastern |
| Teklay 2013 | 96 | 267 | Adults with type 2 diabetes | fbg | Ethiopia | cross-sectional | Eastern |
| Thuita 2019 | 34 | 153 | 20-79 years age and type 2 diabetes | hba1c | Kenya | cross-sectional | Eastern |
| Woldu 2014 | 51 | 102 | Adults with type 2 diabetes | fbg | Ethiopia | cross-sectional | Eastern |
| Yigazu 2017 | 71 | 174 | Adults with type 2 diabetes | fbg | Ethiopia | cross-sectional | Eastern |
| Yimam 2020 | 120 | 300 | Adults with type 2 diabetes | fbg | Ethiopia | cross-sectional | Eastern |
| Yosef 2021 | 88 | 245 | Adults with type 2 diabetes | fbg | Ethiopia | cross-sectional | Eastern |
