# Supplementary material for: Glycaemic control among type 2 diabetes patients in sub-Saharan Africa from 2012 to 2022: a systematic review and meta-analysis
Source: Diabetol Metab Syndr. 2022 Sep 20;14:134. doi: 10.1186/s13098-022-00902-0 (PMC9487067; doi:10.1186/s13098-022-00902-0)
Supplement: Supplementary file 4 — Additional file 4: Table S4. Assessment of methodological quality for included cross-sectional studies. Assessment of the risk of bias for cross-sectional studies with the Joanna Briggs checklist. [file 13098_2022_902_MOESM4_ESM.docx]

**Additional file 4: Table S4** Assessment of methodological quality for included cross-sectional studies

|  | **First author  surname** | **Year of publication** | **Q1** | **Q2** | **Q3** | **Q4** | **Q5** | **Q6** | **Q7** | **Q8** | **Quality  of study** |
| --- | --- | --- | --- | --- | --- | --- | --- | --- | --- | --- | --- |
| 1 | Achila [16] | 2020 | Y | Y | N | Y | N | N | Y | Y | Moderate |
| 2 | Adejumo [17] | 2012 | Y | N | U | Y | N | N | U | Y | Poor |
| 3 | Adeniyi [18] | 2016 | Y | Y | Y | Y | N | Y | Y | Y | Good |
| 4 | Afolabi [19] | 2018 | Y | N | Y | Y | N | N | Y | Y | Moderate |
| 5 | Akabwai [20] | 2016 | Y | Y | Y | Y | N | N | Y | Y | Moderate |
| 6 | Akpalu [21] | 2018 | Y | N | Y | Y | Y | Y | Y | Y | Good |
| 7 | Anioke [22] | 2019 | Y | Y | Y | Y | Y | Y | Y | Y | Good |
| 8 | Ayele [25] | 2019 | Y | Y | Y | Y | N | N | Y | Y | Moderate |
| 9 | Belay [26] | 2017 | Y | Y | U | Y | N | Y | N | Y | Moderate |
| 10 | BeLue [27] | 2016 | Y | Y | Y | Y | N | N | Y | Y | Moderate |
| 11 | Biadgo B [28] | 2018 | Y | Y | Y | Y | N | N | N | Y | Moderate |
| 12 | Biru [29] | 2017 | Y | N | U | Y | N | N | U | Y | Poor |
| 13 | Blum [30] | 2018 | Y | Y | Y | Y | N | N | Y | Y | Moderate |
| 14 | Botchway [31] | 2021 | Y | Y | Y | Y | Y | Y | Y | Y | Good |
| 15 | Camara [32] | 2015 | Y | Y | U | Y | N | N | Y | Y | Moderate |
| 16 | Dagnew [33] | 2017 | Y | Y | Y | Y | N | N | N | Y | Moderate |
| 17 | Demoz [34] | 2019 | Y | Y | Y | Y | N | N | U | Y | Moderate |
| 18 | Doglikuu [35] | 2021 | N | Y | Y | Y | N | N | Y | Y | Moderate |
| 19 | Eticha [36] | 2016 | Y | Y | U | Y | N | N | U | Y | Poor |
| 20 | Fekadu [39] | 2019 | Y | Y | Y | Y | N | N | N | Y | Moderate |
| 21 | Fseha [40] | 2017 | Y | Y | Y | Y | N | N | N | Y | Moderate |
| 22 | Gebremedhin [42] | 2019 | Y | Y | Y | Y | N | N | N | Y | Moderate |
| 23 | Id [44] | 2021 | Y | Y | Y | Y | N | N | N | Y | Moderate |
| 24 | Inih [45] | 2018 | N | N | Y | Y | N | N | Y | Y | Poor |
| 25 | Kalain [46] | 2020 | Y | Y | U | Y | N | N | U | Y | Poor |
| 26 | Kamuhabwa [47] | 2014 | Y | Y | N | Y | N | Y | N | Y | Moderate |
| 27 | Kassahun [48] | 2016 | Y | Y | Y | Y | N | N | N | Y | Moderate |
| 28 | Kefale [49] | 2019 | Y | Y | Y | Y | N | N | N | Y | Moderate |
| 29 | Khoza [50] | 2018 | Y | Y | Y | Y | N | N | Y | Y | Moderate |
| 30 | Kimando [51] | 2017 | Y | Y | Y | Y | N | N | Y | Y | Moderate |
| 31 | Mashele [56] | 2019 | Y | Y | Y | Y | N | N | Y | Y | Moderate |
| 32 | Mobula [58] | 2018 | Y | Y | Y | Y | N | N | Y | Y | Moderate |
| 33 | Mohammed [60] | 2020 | Y | Y | Y | Y | N | N | N | Y | Moderate |
| 34 | Mphwanthe G[61] | 2020 | N | N | Y | Y | Y | Y | Y | Y | Moderate |
| 35 | Mphwantwe[62] | 2020 | Y | Y | Y | Y | N | N | Y | Y | Moderate |
| 36 | Mwavua [64] | 2016 | Y | Y | Y | Y | N | N | Y | Y | Moderate |
| 37 | Mwita [65] | 2019 | Y | Y | Y | Y | N | N | U | Y | Moderate |
| 38 | Noor [66] | 2017 | Y | Y | Y | Y | N | N | Y | Y | Moderate |
| 39 | Omar [67] | 2018 | Y | Y | Y | Y | N | N | U | Y | Moderate |
| 40 | Osuji [68] | 2018 | Y | Y | Y | Y | N | N | Y | Y | Moderate |
| 41 | Otieno [69] | 2017 | Y | Y | Y | Y | N | N | Y | Y | Moderate |
| 42 | Oyewole [70] | 2019 | N | Y | N | Y | N | N | Y | Y | Poor |
| 43 | Ramkisson [72] | 2016 | Y | N | N | Y | N | Y | Y | Y | Moderate |
| 44 | Rwegerera [73] | 2019 | Y | Y | N | Y | N | N | Y | Y | Moderate |
| 45 | Shimels [75] | 2018 | Y | Y | Y | Y | N | N | N | Y | Moderate |
| 46 | Tefera [77] | 2020 | Y | Y | Y | Y | N | N | N | Y | Moderate |
| 47 | Tekalegn [78] | 2018 | Y | Y | Y | Y | N | Y | N | Y | Moderate |
| 48 | Teklay [79] | 2013 | Y | Y | Y | Y | N | N | N | Y | Moderate |
| 49 | Thuita [80] | 2019 | Y | N | Y | Y | N | N | Y | Y | Moderate |
| 50 | Woldu [83] | 2014 | Y | Y | Y | Y | N | N | N | Y | Moderate |
| 51 | Yigazu [85] | 2017 | Y | Y | Y | Y | N | N | N | Y | Moderate |
| 52 | Yimam [86] | 2020 | Y | Y | Y | Y | N | N | N | Y | Moderate |
| 53 | Yosef [87] | 2021 | Y | Y | Y | Y | N | N | N | Y | Moderate |
| 54 | Abera [88] | 2022 | Y | Y | Y | Y | N | Y | Y | Y | Good |
| 55 | Abebe [89] | 2022 | Y | Y | Y | Y | N | N | N | Y | Moderate |
| All (%) | | | 93 | 85 | 80 | 100 | 7 | 18 | 51 | 100 |  |

Legend: Q1. Were the criteria for inclusion in the sample clearly defined? Q2. Were the study subjects and the setting described in detail? Q3. Was the exposure measured in a valid and reliable way? Q4. Were objective, standard criteria used for measurement of the condition? Q5. Were confounding factors identified? Q6. Were strategies to deal with confounding factors stated? Q7. Were the outcomes measured in a valid and reliable way? Q8. Was appropriate statistical analysis used? Y: Yes, N: No, U: Unknown.
