# Supplementary material for: Glycaemic control among type 2 diabetes patients in sub-Saharan Africa from 2012 to 2022: a systematic review and meta-analysis
Source: Diabetol Metab Syndr. 2022 Sep 20;14:134. doi: 10.1186/s13098-022-00902-0 (PMC9487067; doi:10.1186/s13098-022-00902-0)
Supplement: Supplementary file 5 — Additional file 5: Table S5. Assessment of methodological quality of quasi-experimental studies. Assessment of the risk of bias for quasi-experimental studies with the Joanna Briggs checklist. [file 13098_2022_902_MOESM5_ESM.docx]

**Additional file 5: Table S5** Assessment of methodological quality of quasi-experimental studies

|  | **First author  surname** | **Year of publication** | **Q1** | **Q2** | **Q3** | **Q4** | **Q5** | **Q6** | **Q7** | **Q8** | **Q9** | **Quality  of study** |
| --- | --- | --- | --- | --- | --- | --- | --- | --- | --- | --- | --- | --- |
| 1 | Assah [24] | 2015 | Y | Y | Y | Y | Y | Y | N | U | Y | Moderate |
| 2 | Hailu [43] | 2018 | Y | Y | Y | Y | Y | Y | Y | Y | Y | Good |
| 3 | Mash [54] | 2016 | Y | Y | Y | Y | Y | Y | Y | Y | Y | Good |
| 4 | Rambiritch [71] | 2014 | Y | Y | Y | N | Y | Y | Y | N | Y | Moderate |
| All (%) | | | 100 | 100 | 100 | 75 | 100 | 100 | 75 | 50 | 100 |  |

Legend: Q1. Is it clear in the study what is the ‘cause’ and what is the ‘effect’ (i.e. there is no confusion about which variable comes first)? Q2. 2. Were the participants included in any comparisons similar? Q3. Were the participants included in any comparisons receiving similar treatment/care, other than the exposure or intervention of interest? Q4. Was there a control group? Q5. Were there multiple measurements of the outcome both pre- and post-intervention/exposure? Q6. Was follow-up complete and if not, were differences between groups in terms of their follow-up adequately described and analysed? Q7. Were the outcomes of participants included in any comparisons measured in the same way? Q8. Were outcomes measured in a reliable way? Q9. Was appropriate statistical analysis used? Y: Yes, N: No, U: Unknown.
