# Supplementary material for: Glycaemic control among type 2 diabetes patients in sub-Saharan Africa from 2012 to 2022: a systematic review and meta-analysis
Source: Diabetol Metab Syndr. 2022 Sep 20;14:134. doi: 10.1186/s13098-022-00902-0 (PMC9487067; doi:10.1186/s13098-022-00902-0)
Supplement: Supplementary file 6 — Additional file 6: Table S6. Assessment of methodological quality for included randomized controlled trials. Assessment of the risk of bias for randomized controlled trials [file 13098_2022_902_MOESM6_ESM.docx]

**Additional file 6: Table S6.**Assessment of methodological quality for included randomized controlled trials

|  | **First author  surname** | **Year of publication** | **Q1** | **Q2** | **Q3** | **Q4** | **Q5** | **Q6** | **Q7** | **Q8** | **Q9** | **Q10** | **Q11** | **Q12** | **Q13** | **Quality  of study** |
| --- | --- | --- | --- | --- | --- | --- | --- | --- | --- | --- | --- | --- | --- | --- | --- | --- |
| 1 | Anyanwu [23] | 2016 | Y | U | Y | Y | N | N | Y | Y | Y | Y | Y | Y | Y | Moderate |
| 2 | Ezema [37] | 2014 | Y | U | Y | N | N | N | Y | Y | Y | Y | N | Y | Y | Moderate |
| 3 | Fayehun [38] | 2018 | Y | Y | Y | Y | Y | U | Y | Y | Y | Y | Y | Y | Y | Good |
| 4 | Gathu [41] | 2018 | Y | Y | Y | Y | Y | U | Y | Y | Y | Y | Y | Y | Y | Good |
| 5 | Maharaj [52] | 2016 | Y | U | Y | Y | Y | U | Y | Y | Y | Y | Y | Y | Y | Good |
| 6 | Mash [55] | 2014 | Y | Y | N | N | N | N | Y | Y | Y | Y | Y | Y | Y | Moderate |
| 7 | Muchiri [63] | 2016 | Y | Y | Y | N | Y | N | Y | Y | Y | Y | Y | Y | Y | Good |
| 8 | Thuita [81] | 2020 | Y | Y | Y | N | N | N | Y | Y | Y | Y | Y | Y | Y | Moderate |
| 9 | Tsobgny-Tsague [82] | 2018 | Y | Y | Y | Y | Y | Y | Y | Y | Y | Y | Y | Y | Y | Good |
| 10 | Yan [84] | 2014 | Y | N | N | N | N | N | N | Y | Y | Y | Y | Y | Y | Moderate |
| All (%) | | | 100 | 60 | 80 | 50 | 50 | 10 | 90 | 100 | 100 | 100 | 90 | 100 | 100 |  |

Legend: Q1. Was true randomization used for the assignment of participants to treatment groups? Q2. Was allocation to treatment
groups concealed? Q3. Were treatment groups similar at the baseline? Q4. Were participants blind to treatment assignment?
Q5. Were those delivering treatment blind to treatment assignment? Q6. Were outcomes assessors blind to treatment assignment?
Q7. Were treatment groups treated identically other than the intervention of interest? Q8. Was follow up complete and if not,
were differences between groups in terms of their follow up adequately described and analysed? Q9. Were participants analysed
in the groups to which they were randomized? Q10. Were outcomes measured in the same way for treatment groups?
Q11. Were outcomes measured in a reliable way? Q12. Was appropriate statistical analysis used? Q13. Was the trial design
appropriate, and any deviations from the standard RCT design (individual randomization, parallel groups) accounted for in the
conduct and analysis of the trial? Y: Yes, N: No, U: Unknown.
