# Supplementary material for: Glycaemic control among type 2 diabetes patients in sub-Saharan Africa from 2012 to 2022: a systematic review and meta-analysis
Source: Diabetol Metab Syndr. 2022 Sep 20;14:134. doi: 10.1186/s13098-022-00902-0 (PMC9487067; doi:10.1186/s13098-022-00902-0)
Supplement: Supplementary file 7 — Additional file 7: Table S7. Assessment of methodological quality of case–control studies. The cross-sectional studies assessed with the Joanna Briggs checklist. [file 13098_2022_902_MOESM7_ESM.docx]

**Additional file 7: Table S7.** Assessment of methodological quality of case-control studies

|  | **First author  surname** | **Year of publication** | **Q1** | **Q2** | **Q3** | **Q4** | **Q5** | **Q6** | **Q7** | **Q8** | **Q9** | **Q10** | **Quality of study** |
| --- | --- | --- | --- | --- | --- | --- | --- | --- | --- | --- | --- | --- | --- |
| 1 | Mamo [53] | 2019 | Y | N | Y | Y | Y | N | Y | N | Y | Y | Moderate |
| 2 | Mohamed [59] | 2013 | Y | Y | Y | Y | Y | Y | Y | Y | N | Y | Good |
| 3 | Sarfo-Kantanka [74] | 2017 | Y | Y | Y | Y | Y | N | Y | Y | N | Y | Good |
| All (%) | | | 100 | 66.7 | 100 | 100 | 100 | 33.3 | 100 | 66.7 | 33.3 | 100 |  |

Legend: Q1. Were the groups comparable other than the presence of disease in cases or the absence of disease in controls? Q2. Were cases and controls matched appropriately? Q3. Were the same criteria used for the identification of cases and controls? Q4. Was exposure measured in a standard, valid and reliable way? Q5. Was exposure measured in the same way for cases and controls? Q6. Were confounding factors identified? Q7. Were strategies to deal with confounding factors stated? Q8. Were outcomes assessed in a standard, valid and reliable way for cases and controls?
