# Supplementary material for: Glycaemic control among type 2 diabetes patients in sub-Saharan Africa from 2012 to 2022: a systematic review and meta-analysis
Source: Diabetol Metab Syndr. 2022 Sep 20;14:134. doi: 10.1186/s13098-022-00902-0 (PMC9487067; doi:10.1186/s13098-022-00902-0)
Supplement: Supplementary file 8 — Additional file 8: Table S8. Assessment of methodological quality of cohort study. Assessment of the risk of bias for cohort study through the Joanna Briggs checklist. [file 13098_2022_902_MOESM8_ESM.docx]

**Additional file 8: Table S8.** Assessment of methodological quality of cohort study

|  | **First author surname** | **Year of publication** | **Q1** | **Q2** | **Q3** | **Q4** | **Q5** | **Q6** | **Q7** | **Q8** | **Q9** | **Q10** | **Q11** | **Quality of study** |
| --- | --- | --- | --- | --- | --- | --- | --- | --- | --- | --- | --- | --- | --- | --- |
| 1 | Siddiqui [76] | 2018 | N | Y | Y | N | N | Y | Y | Y | N | N | Y | Moderate |
| 2 | Mayet [57] | 2012 | Y | Y | Y | N | N | N | Y | Y | Y | N | Y | Moderate |
| All (%) | | | 50 | 100 | 100 | 0 | 0 | 50 | 100 | 100 | 50 | 0 | 100 |  |

Legend: Q1. Were the two groups similar and recruited from the same population? Q2. Were the exposures measured similarly to assign people to both exposed and unexposed groups? Q3. Was the exposure measured in a valid and reliable way? Q4. Were confounding factors identified? Q5. Were strategies to deal with confounding factors stated? Q6. Were the groups/participants free of the outcome at the start of the study (or at the moment of exposure)? Q7. Were the outcomes measured in a valid and reliable way? Q8. Was the follow-up time reported and sufficient to be long enough for outcomes to occur? Q9. Was follow-up complete, and if not, were the reasons to loss to follow-up described and explored? Q10. Were strategies to address incomplete follow-up utilized? Q11. Was appropriate statistical analysis used? Y: Yes, N: No.
