# Supplementary material for: Glycaemic control among type 2 diabetes patients in sub-Saharan Africa from 2012 to 2022: a systematic review and meta-analysis
Source: Diabetol Metab Syndr. 2022 Sep 20;14:134. doi: 10.1186/s13098-022-00902-0 (PMC9487067; doi:10.1186/s13098-022-00902-0)
Supplement: Supplementary file 9 — Additional file 9. List of references of excluded studies. The list of excluded references [file 13098_2022_902_MOESM9_ESM.docx]

LIST OF REFERENCES OF EXCLUDED STUDIES

1. Abubakar B, Giaze RT, Haliru A. Clinical investigation of treatment failure in type 2 diabetic patients treated with metformin and glibenclamide at a hospital in northwestern Nigeria. Trop J Pharm Res. 2014;13(9):1521–6.

2. Abutair AS, Naser IA, Hamed AT. The effect of soluble fiber supplementation on metabolic syndrome profile among newly diagnosed type 2 diabetes patients. Clin Nutr Res [Internet]. 2018;7(1):31–9. Available from: https://e-cnr.org/search.php?where=aview&id=10.7762/cnr.2018.7.1.31&code=9994CNR&vmode=FULL

3. Adeniyi AF, Uloko AE, Ogwumike OO, Sanya AO, Fasanmade AA. Time course of improvement of metabolic parameters after a 12 week physical exercise Programme in patients with type 2 diabetes: The influence of gender in a Nigerian population. Biomed Res Int [Internet]. 2013 [cited 2021 Apr 19];2013. Available from: /pmc/articles/PMC3773397/

4. Adisa R, Olajide OO, Fakeye TO. Social Support, Treatment Adherence and Outcome among Hypertensive and Type 2 Diabetes Patients in Ambulatory Care Settings in southwestern Nigeria. Ghana Med J. 2017;51(2):64–77.

5. Adisa R, Fakeye TO. Do diabetes-specialty clinics differ in management approach and outcome? A cross-sectional assessment of ambulatory type 2 diabetes patients in two teaching hospitals in Nigeria. Ghana Med J [Internet]. 2016;50(2):90–102. Available from: http://www.ghanamedj.org/articles/June2016/Final Diabetes Specialist Clinics.pdf

6. Adisa R, Fakeye TO. Effect of number and type of antidiabetes medications on adherence and glycemia of

ambulatory type 2 diabetes patients in southwestern Nigeria. Pharm Pract (Granada) [Internet]. 2013 Sep.

19 [cited 2022 Apr. 22];11(3):156-65. Available from:

<https://www.pharmacypractice.org/index.php/pp/article/view/346>

7. Aga F, Dunbar SB, Kebede T, Higgins MK, Gary R. Sociodemographic and clinical correlates of diabetes self-efficacy in adults with type 2 diabetes and comorbid heart failure. Res Nurs Health. 2020;43(1):79–89.

8. AmendezoEtienne, Walker Timothy D, Karamuka V, Robinson B, Kavabushi P, Ntirenganya C, et al. Effects of a lifestyle education program on glycemic control among patients with diabetes at Kigali University Hospital, Rwanda: A randomized controlled trial. Diabetes Res Clin Pract. 2017;126:129–37.

9. Anakwue RC, Young EE, Ezenduka CC, Okoli CI, Maduforo AN, Nnakenyi ID, et al. Assessment of patients knowledge and attitude towards diabetes and its relationship with glycemic control: a cross-sectional study in a Nigerian tertiary hospital. Niger J Med [Internet]. 2019; Available from: <https://search.bvsalud.org/gim/resource/fr/afr-201545>

10. Anetor JI, Uche CZ, Ayita EB, Adedapo SK, Adeleye JO, Anetor GO, et al. Cadmium Level, Glycemic Control, and Indices of Renal Function in Treated Type NN Diabetics: Implications for Polluted Environments. Front Public Heal. 2016;4.

11. Angamo MT, Melese BH, Ayen WY. Determinants of Glycemic Control among Insulin Treated Diabetic Patients in Southwest Ethiopia: Hospital Based Cross Sectional Study. PLoS One. 2013;8(4).

12. Anyakudo MMC. Effects of food processing methods on diets proximate nutrient composition and glycemic profile in male type 2 diabetic subjects. Br J Appl Sci Technol [Internet]. 2014;4(27):3995–4005. Available from: http://www.sciencedomain.org/abstract.php?iid=613&id=5&aid=5514

13. Asfaw A, Mamo N, Anshabo A, Hawaze S. Assessment of type II diabetes mellitus drug therapy in diabetes clinic of a tertiary care teaching hospital in Addis Ababa. Arch Pharm Pract. 2014;5(3):113.

14. Awadalla H, Noor SK, Elmadhoun WM, Almobarak AO, Elmak NE, Abdelaziz SI, et al. Diabetes complications in Sudanese individuals with type 2 diabetes: Overlooked problems in sub-Saharan Africa? Diabetes Metab Syndr Clin Res Rev. 2017;11:S1047–51.

15. Awadalla H, Noor SK, Elmadhoun WM, Bushara SO, Almobarak AO, Sulaiman AA, et al. Comparison of serum lipid profile in type 2 diabetes with and without adequate diabetes control in Sudanese population in north of Sudan. Diabetes Metab Syndr Clin Res Rev. 2018;12(6):961–4.

16. Awodele O, Osuolale JA. Medication adherence in type 2 diabetes patients: study of patients in Alimosho General Hospital, Igando, Lagos, Nigeria. Afr Health Sci. 2015;15(2):513–22.

17. Azar ST, Echtay A, Wan Bebakar WM, Al Araj S, Berrah A, Omar M, et al. Efficacy and safety of liraglutide compared to sulphonylurea during Ramadan in patients with type 2 diabetes (LIRA-Ramadan): a randomized trial. Diabetes Obes Metab. 2016 Jan 1;18(10):1025–33.

18. Azenabor A, Ogbera AO, Ogunyemi EO, Okafor CJ. Oxidative stress and glycaemic control determinants in Type 2 DM. Pakistan J Med Sci. 2011;27(4):739–43.

19. Azubike CO, Unuigbe EI. Progression of diabetic nephropathy: A twelve-year follow-up of type2 diabetic patients. J Med Biomed Res. 2013;12(1):105–12.

20. Bello-Ovosi BO, Ovosi JO, Ogunsina MA, Asuke S, Ibrahim MS. Prevalence and pattern of dyslipidemia in patients with type 2 diabetes mellitus in Zaria, Northwestern Nigeria. Pan Afr Med J [Internet]. 2019;34. Available from: https://www.panafrican-med-journal.com/content/article/34/123/full/

21. Biadgo B, Melku M, Abebe SM, Abebe M. Hematological indices and their correlation with fasting blood glucose level and anthropometric measurements in type 2 diabetes mellitus patients in Gondar, Northwest Ethiopia. Diabetes Metab Syndr Obesity-Targets Ther. 2016;9:91–9.

22. Botha S, Welsh P, Logue J, North-West University SA. The effect of non-surgical weight management on weight and glycaemic control in people with type 2 diabetes: Three-year outcomes from a real-life programme. Journal of Endocrinology, Metabolism and Diabetes of South Africa. 2017;22(10).

23. Bulbulia S, Variava F, Bayat Z. Are type 2 diabetic patients meeting targets? A Helen Joseph Hospital Diabetic Clinic Audit. J Endocrinol Metab Diabetes South Africa. 2020;25(1):12–7.

24. Correia JC, Lopes A, Nhabali A, Madrigal V, Errasti CR, Brady E, et al. Implementation and evaluation of a specialized diabetes clinic in Guinea-Bissau: lessons learnt from the field. Pan Afr Med J [Internet]. 2020;37. Available from: https://www.panafrican-med-journal.com/content/article/37/126/full/

25. Dalia IWA, Abdelmula MA, Zeinab AE, AbdElkarim AA. Association of vitamin D with diabetic neuropathy among Sudanese patients with type 2 diabetes mellitus. Niger J Basic Clin Sci [Internet]. 2019;16(2):78–82. Available from: http://www.njbcs.net/article.asp?issn=0331-8540

26. Darko SN, Yar DD, Owusu-Dabo E, Awuah AA-AA, Dapaah W, Addofoh N, et al. Variations in levels of IL-6 and TNF-α in type 2 diabetes mellitus between rural and urban Ashanti Region of Ghana. BMC Endocr Disord [Internet]. 2015 Sep;15(1):1–7. Available from: http://dx.doi.org/10.1186/s12902-015-0047-9

27. Danquah I, Bedu-Addo G, Terpe KJ, Micah F, Amoako YA, Awuku YA, et al. Diabetes mellitus type 2 in urban Ghana: characteristics and associated factors. BMC Public Health [Internet]. 2012;12(210):(20 March 2012). Available from: http://www.biomedcentral.com/content/pdf/1471-2458-12-210.pdf

28. Diaf M, Khaled BM, Sellam F. Impact of corpulence parameters and haemoglobin A1c on metabolic control in type 2 diabetic patients: Comparison of apolipoprotein B/A-I ratio with fasting and postprandial conventional lipid ratios. Libyan J Med [Internet]. 2015 [cited 2021 Apr 17];10(1). Available from: https://pubmed.ncbi.nlm.nih.gov/28349802/

29. Dickie K, Micklesfield LK, Chantler S, Lambert E V, Goedecke JH. Meeting physical activity guidelines is associated with reduced risk for cardiovascular disease in black South African women; a 5.5-year follow-up study. BMC public health. 2014;14: 498.

30. Diouf NN, Lo G, Djité M, Tine JAD, Diatta A. Evaluation de la microalbuminurie et du profil lipidique chez les diabétiques de type 2 diabetics. 2015;10–3.

31. Diouf NN, Boye O, Soumboundou M, Guèye MW, Sawaré EM. Article original Evaluation de l ’ équilibre glycémique chez les sujets âgés présentant un diabète de type 2 Assessment of glycemic control in elderly patients with type 2 diabetes. 2013;3(2):269–72.

32. Distiller LA, Cranston I, Mazze R. First Clinical Experience with Retrospective Flash Glucose Monitoring (FGM) Analysis in South Africa: Characterizing Glycemic Control with Ambulatory Glucose Profile. J Diabetes Sci Technol. 2016;10(6):1294–302.

33. Edo A, Adediran OS. Dyslipidaemia among Nigerian oil workers with type 2 diabetes mellitus. West Afr J Med. 2011;30(3):206–9.

34. Elkhidir AE, Eltaher HB, Mohamed AO. Association of lipocalin-2 level, glycemic status and obesity in type 2 diabetes mellitus. BMC Res Notes [Internet]. 2017 Jul 14 [cited 2021 Apr 17];10(1):285. Available from: http://bmcresnotes.biomedcentral.com/articles/10.1186/s13104-017-2604-y

35. Essien O, Otu A, Umoh V, Enang O, Hicks JP, Walley J. Intensive patient education improves glycaemic control in diabetes compared to conventional education: A randomised controlled trial in a nigerian tertiary care hospital. PLoS One. 2017;12(1):1–12.

36. Ewenighi CO, Uchechukwu D, Adejumo BI, Onyeanusi JC, Nnatuanya IN, Onoh OG, et al. Responses to glycemic control therapy according to age, gender, level of adiposity, and duration of diabetes in type 2 diabetic patients. Indian J Med Sci [Internet]. 2013;67(3/4):61–9. Available from: http://www.indianjmedsci.org/article.asp?issn=0019-5359

37. Ezenwaka CE, Okoye O, Esonwune C, Dioka C, Onuoha P, Osuji C, et al. Is diabetes patients’ knowledge of laboratory tests for monitoring blood glucose levels associated with better glycaemic control? Arch Physiol Biochem. 2014;120(2):86–90.

38. Farmer A, Bobrow K, Leon N, Williams N, Phiri E, Namadingo H, et al. Mobile Messaging Support Versus Usual Care for People With Type 2 Diabetes on Glycemic Control: Protocol for a Multicenter Randomized Controlled Trial. Jmir Res Protoc. 2019;8(6).

39. Fondjo LA, Sakyi SA, Owiredu WKBA, Laing EF, Owiredu E-W, Awusi EK, et al. Evaluating Vitamin D Status in Pre-and Postmenopausal Type 2 Diabetics and Its Association with Glucose Homeostasis. 2018; Available from: https://doi.org/10.1155/2018/9369282

40. Geneto M, Umeta M, Kebede T, Azazh A, Nagphaul R, Mohammed SF. A comparative study on serum level concentration of micronutrients like zinc, copper and chromium status in type 2 diabetic patients in diabetes & endocrinology unit, Tikur Anbessa Specialized Hospital, Ethiopia. J Pharm Nutr Sci. 2015;5(2):95–102.

41. Gill JM, Ross A, Pirie F, Esterhuizen T. The effect of the introduction of a standard monitoring protocol on the investigations performed on the metabolic control of type 2 diabetes at Addington Hospital Medical Outpatients Department, Durban, South Africa. Vol. 54, South African Family Practice. 2012;54.

42. Hall KK, Tambekou J, Penn L, Camara A, Balde NM, Sobngwi E. Association between depression, glycaemic control and the prevalence of diabetic retinopathy in a diabetic population in Cameroon. South African Journal of Psychiatry. 2017;23.

43. Idonije BO, Festus O, Oluba OM. Plasma glucose, creatinine and urea levels in type 2 diabetic patients attending a Nigerian teaching hospital. Res J Med Sci. 2011;5(1):1–3.

44. Iloh G. Family functionality, medication adherence and blood glucose control among ambulatory type 2 diabetic patients in a Nigerian hospital. J Basic Clin Pharm. 2017;8(3):149–53.

45. Iloh GP, Collins P, Amadi A. Family functionality, medication adherence, and blood glucose control among ambulatory type 2 diabetic patients in a primary care clinic in Nigeria. Int J Heal Allied Sci. 2018;7(1):23.

46. Iwuala SO, Olamoyegun MA, Sabir AA, Fasanmade OA. The relationship between self-monitoring of blood glucose and glycaemic control among patients attending an urban diabetes clinic in Nigeria. Ann Afr Med. 2015;14(4):182–7.

47. Jackson IL, Onung SI, Oiwoh EP. Self-care activities, glycaemic control and health-related quality of life of patients with type 2 diabetes in a tertiary hospital in Nigeria. Diabetes Metab Syndr Res Rev. 2021;15(1):137–43.

48. Jemal A, Abdela J, Sisay M. Adherence to Oral Antidiabetic Medications among Type 2 Diabetic (T2DM) Patients in Chronic Ambulatory Wards of Hiwot Fana Specialized University Hospital, Harar, Eastern Ethiopia: A Cross Sectional Study. J Diabetes Metab. 2017;8(1).

49. Karau PB, Kirna B, Amayo E, Joshi M, Ngare S, Muriira G. The prevalence of vitamin D deficiency among patients with type 2 diabetes seen at a referral hospital in Kenya. Pan Afr Med J. 2019;34:1–11.

50. Leulseged Id TW, Id BTA. Time to optimal glycaemic control and prognostic factors among type 2 diabetes mellitus patients in public teaching hospitals in Addis Ababa, Ethiopia. 2019; Available from: https://doi.org/10.1371/journal.pone.0220309

51. Madela S, James S, Sewpaul R, Madela S, Reddy P. Early detection, care and control of hypertension and diabetes in South Africa: A community-based approach. African J Prim Heal Care &amp; Fam Med [Internet]. 2020; Available from: https://search.bvsalud.org/gim/resource/fr/afr-202250

52. Makan G, Bayat Z, Arumugam A, Henning E, Helen Joseph Hospital SA. Effect of a multi-disciplinary approach in the treatment of type 2 diabetes mellitus. Journal of Endocrinology, Metabolism and Diabetes of South Africa. 2013;18:1.

53. Mashitisho MLI, Mashitisho BG, University SMHS. Early insulin therapy in patients with type 2 diabetes mellitus. J Endocrinol Metab Diabetes South Africa. 2016 Jan 1;21(1):50–4.

54. Matheka DM, Kilonzo JM, Munguti CM, Mwangi PW. Pattern, knowledge and practices of HbA1C testing among diabetic patients in a Kenyan tertiary referral hospital. Global Health [Internet]. 2013;9(1):1. Available from: Globalization and Health

55. Matshipi M, Monyeki KD, Kemper H. The relationship between physical activity and plasma glucose level amongst ellisras rural young adult males and females: Ellisras longitudinal study. Int J Environ Res Public Health [Internet]. 2017 Feb 16 [cited 2021 Apr 17];14(2). Available from: https://pubmed.ncbi.nlm.nih.gov/28212346/

56. Mbwete GW, Kilonzo KG, Shao ER, Chamba NG. Suboptimal Blood Pressure Control, Associated Factors, and Choice of Antihypertensive Drugs among Type 2 Diabetic Patients at KCMC, Tanzania. J Diabetes Res. 2020;2020.

57. Mbouemboue OP, Tsougmo JON, Sakinatou I, Ndolok AC V, Tamanji MT. Prevalence, treatment, and control of diabetes in a community setting in Ngaoundéré, Cameroon. Med Sante Trop [Internet]. 2018;28(4):379–84. Available from: http://www.medsantetrop.com

58. Mels CM, Schutte AE, Schutte R, Huisman HW, Smith W, Fourie CM, et al. The link between vascular deterioration and branched chain amino acids in a population with high glycated haemoglobin: the SABPA study. Amino Acids. 2013;45:1405–13.

59. Mogre V, Abanga ZO, Tzelepis F, Johnson NA, Paul C. Psychometric evaluation of the summary of diabetes self-care activities measure in Ghanaian adults living with type 2 diabetes. Diabetes Res Clin Pract. 2019 Jan 1;149:98–106.

60. Mohamed DA, Al-Okbi SY, El-Hariri DM, Mousa II. Potential health benefits of bread supplemented with defatted flaxseeds under dietary regimen in normal and type 2 diabetic subjects. Polish J Food Nutr Sci [Internet]. 2012;62(2):103–8. Available from: http://www.pan.olsztyn.pl/journal

61. Monanabela KB, van Huyssteen M, Coetzee R. Describing medicine therapy management of type 2 diabetes mellitus at primary health care facilities in cape town. Heal SA Gesondheid. 2019;24:1–8.

62. Motta LA, Shephard MDSS, Brink J, Lawson S, Rheeder P, MottaLara A, et al. Point-of-care testing improves diabetes management in a primary care clinic in South Africa. Prim Care Diabetes. 2017 Jan 1 ;11(3):248–53.

63. Moustafa HAM, El-Wakeel LM, Halawa MR, Sabri NA, El-Bahy AZ, Singab AN. Effect of Nigella sativa oil versus metformin on glycemic control and biochemical parameters of newly diagnosed type 2 diabetes mellitus patients. Endocrine. 2019;65(2):286–94.

64. Muddu M, Mutebi E, Ssinabulya I, Kizito S, Mondo CK. Hypertension among newly diagnosed diabetic patients at Mulago national referral hospital in Uganda a cross sectional study. Cardiovasc J Afr [Internet]. 2018 Jul 1 [cited 2021 Apr 17];29(4):218–24. Available from: https://pubmed.ncbi.nlm.nih.gov/29750228/

65. Mullugeta Y, Chawla R, Kebede T, Worku Y. Dyslipidemia associated with poor glycemic control in type 2 diabetes mellitus and the protective effect of metformin supplementation. Indian J Clin Biochem. 2012;27(4):363–9.

66. Munyogwa MJ, William R, Kibusi SM, Gibore NS. Clinical characteristics and health care received among patients with type 2 diabetes attending secondary and tertiary healthcare facilities in Mwanza Region, Tanzania: a cross-sectional study. BMC Health Serv Res [Internet]. 2020;20(527). Available from: https://link.springer.com/article/10.1186/s12913-020-05407-y

67. Musenge EM, Michelo C, Mudenda B, Manankov A. Glycaemic Control and Associated Self-Management Behaviours in Diabetic Outpatients: A Hospital Based Observation Study in Lusaka, Zambia. 2016; Available from: http://dx.doi.org/10.1155/2016/7934654

68. Neboh EE, Ikekpeazu EJ, Maduka IC, Aduba O, Ibegbu DM, Aniagolu MO, et al. Lipid profile, aip and glycated haemoglobin level in type-2 diabetic patients in Enugu, South-East Nigeria. J Asian Sci Res. 2012;2(5):300–6.

69. Nduati NJ. Factors Associated With Glycemic Control among Type 2 Diabetes Patients Attending Mathari National Teaching Hospital, Nairobi Kenya. J Endocrinol Diabetes . 2016;3(6):1–11.

70. Ngala RA, Asare AA. Influence of oxidative stress, skeletal muscle mass and obesity on type 2 diabetes mellitus among patients in Kumasi Metropolis. Trends Med Res [Internet]. 2014;9(2):107–15. Available from: http://scialert.net/qredirect.php?doi=tmr.2014.107.115&linkid=pdf

71. Ng’ang’a L, Ngoga G, Dusabeyezu S, Hedt-Gauthier BL, Ngamije P, Habiyaremye M, et al. Implementation of blood glucose self-monitoring among insulin-dependent patients with type 2 diabetes in three rural districts in Rwanda: 6 months open randomised controlled trial. BMJ Open [Internet]. 2020;10(7). Available from: https://bmjopen.bmj.com/content/10/7/e036202

72. Nganou-Gnindjio CN, Mba CM, Azabji-Kenfack M, Dehayem MY, Mfeukeu-Kuate L, Mbanya JC, et al. Poor glycemic control impacts heart rate variability in patients with type 2 diabetes mellitus: A cross sectional study. BMC Res Notes [Internet]. 2018;11(1):1–4. Available from: https://doi.org/10.1186/s13104-018-3692-z

73. Nielsen J, Bahendeka SK, Gregg EW, Whyte SR, Bygbjerg IC, Meyrowitsch DW. A comparison of cardiometabolic risk factors in households in rural Uganda with and without a resident with type 2 diabetes, 2012-2013. Prev Chronic Dis [Internet]. 2015;12(4):E44. Available from: http://www.cdc.gov/pcd/issues/2015/14_0486.htm

74. Nkoana MK, Khine AA. Correlation between self-monitored mean blood glucose and average plasma glucose estimated from glycated haemoglobin in patients attending the diabetes clinic at Dr George Mukhari Academic Hospital, Pretoria, South Africa. S Afr Med J. 2020 Apr;110(5):416–21.

75. Odume BB, Ofoegbu OS, Aniwada EC, Okechukwu EF. The influence of family characteristics on glycaemic control among adult patients with type 2 diabetes mellitus attending the general outpatient clinic, national hospital, Abuja, Nigeria. South African Fam Pract. 2015;57(6):347–53.

76. Ofori EK, Owusu-Ababio D, Tagoe EA, Asare-Anane H. Dyslipidaemia is common among patients with type 2 diabetes: a cross-sectional study at Tema Port Clinic. BMC Res Notes. 2019/04/05. 2019;12(1):204.

77. Okoro N, Okpara H, Azinge E, Builders M. Serum Total Adiponectin and its Relationship with Glycemic Control ad Markers of Lipoprotein Metabolism in Nigerians with Type 2 Diabetes Mellitus. Ann Med Health Sci Res. 2019;9(1):478–83.

78. Olaniyan SI, Fasina O, Bekibele CO, Ogundipe AO. Relationship between dry eye and glycosylated haemoglobin among diabetics in Ibadan, Nigeria. Pan Afr Med J [Internet]. 2019;33. Available from: https://www.panafrican-med-journal.com/content/article/33/14/full/

79. Omar SM, Musa IR, ElSouli A, Adam I. Prevalence, risk factors, and glycaemic control of type 2 diabetes mellitus in eastern Sudan: a community-based study. Ther Adv Endocrinol Metab. 2019;10.

80. Osei-Yeboah J, Owiredu W, Norgbe G, Obirikorang C, Lokpo S, Ashigbi E, et al. Physical Activity Pattern and Its Association with Glycaemic and Blood Pressure Control among People Living with Diabetes (PLWD) In The Ho Municipality, Ghana. Ethiop J Health Sci. 2019;29(1):819–30.

81. Osei-Yeboah J, Lokpo SY, Owiredu WKBA, Johnson BB, Orish VN, Botchway F, et al. Medication Adherence and its Association with Glycaemic Control, Blood Pressure Control, Glycosuria and Proteinuria Among People Living With Diabetes (PLWD) in the Ho Municipality, Ghana. Open Public Health J. 2019;11(1):552–61.

82. Osman H, Ahmed AM, A-Rahman NHA. Demographic and clinical characteristics of diabetic patients attending an outpatient clinic in Omdurman, Sudan. Sudan J Public Heal [Internet]. 2013;8(2):47–51. Available from: http://www.sjph.net.sd/files/Vol8N2/Original_Article1.pdf

83. Owolabi EO, Goon D Ter, Ajayi AI. Efficacy, acceptability and feasibility of daily text-messaging in promoting glycaemic control and other clinical outcomes in a low-resource setting of South Africa: A randomised controlled trial. PLoS One. 2019;14(11):e0224791.

84. Paruk IM, Pir FJ, Nkwanyana NM, Motala AA. Prevalence of low serum testosterone levels among men with type 2 diabetes mellitus attending two outpatient diabetes clinics in KwaZulu-Natal Province, South Africa. SAMJ - South African Med J [Internet]. 2019;109(12):957–62. Available from: http://www.samj.org.za/index.php/samj/article/view/12774/9042

85. Pengpid S, Peltzer K, Skaal L. Efficacy of a church-based lifestyle intervention programme to control high normal blood pressure and/or high normal blood glucose in church members: a randomized controlled trial in Pretoria, South Africa. BMC Public Health. 2014 Jun;14:568.

86. Philis-Tsimikas A, Del Prato S, Satman I, Bhargava A, Dharmalingam M, Skjøth T V, et al. Effect of insulin degludec versus sitagliptin in patients with type 2 diabetes uncontrolled on oral antidiabetic agents. Obe Metab. 2013;15:760–6.

87. Pillay S, Jansen Van Vuuren JM, Jansen Van Vuuren CJ The Magnesium and Glucose (MAG) Study: the prevalence and effect of hypomagnesaemia on diabetes control in a regional hospital in KwaZulu-Natal. Journal of Endocrinology, Metabolism and Diabetes of South Africa [Internet]. 2018;23(1):22–5. Available from: <https://www.tandfonline.com/doi/full/10.1080/16089677.2017.1414731>

88. Pinchevsky Y, Butkow N, Raal FJ, Chirwa T. The implementation of guidelines in a South African population with type 2 diabetes. J Endocrinol Metab Diabetes South Africa. 2013 Jan 1;18(3):154–8.

89. Pinchevsky Y, Butkow N, Chirwa T, Raal FJ. Glycaemic, blood pressure and cholesterol control in 25 629 diabetics. Cardiovasc J Afr. 2015;26(4):188–92.

90. Pinchevsky Y, Shukla VJ, Butkow N, Chirwa T, Raal F. Multi-ethnic differences in HbA1c, blood pressure, and low-density-lipid cholesterol control among South Africans living with type 2 diabetes, after a 4-year follow-up. Int J Gen Med. 2016;9:419–26.

91. Pinchevsky Y, Butkow N, Chirwa T, Raal F, PinchevskyYacob, Butkow N, et al. Treatment Gaps Found in the Management of Type 2 Diabetes at a Community Health Centre in Johannesburg, South Africa. J Diabetes Res. 2017 Jan 1;2017:9536025.

92. Pirie FJ, Maharaj S, Esterhuizen TM, Paruk IM, Motala AA. Retinopathy in subjects with type 2 diabetes at a tertiary diabetes clinic in Durban, South Africa: Clinical, biochemical and genetic factors. J Clin Transl Endocrinol [Internet]. 2014;1(1):e9. Available from: http://dx.doi.org/10.1016/j.jcte.2013.12.002

93. Ramkisson S, Pillay BJ, Sibanda W. Social support and coping in adults with type 2 diabetes. African J Prim Heal care Fam Med. 2017;9(1).

94. Rwegerera GM, Shailemo DHP, Rivera YP, Mokgosi KO, Bale P, Oyewo TA, et al. Metabolic Control and Determinants Among HIV-Infected Type 2 Diabetes Mellitus Patients Attending a Tertiary Clinic in Botswana. Diabetes Metab Syndr Obesity-Targets Ther. 2021;14:85–97.

95. Sadik I, Yagoub Z, Sayed N, El Nour A, El Hameed MA, Abid SB. The Level of Ischemic Modified Albumin (IMA) as Risk Marker for Cardio Vascular Disease (CVD) among some diabetic patients (type II) in Khartoum State-Sudan. Sudan J Med Sci. 2017;12(4):231–9.

96. Shimels T, Asrat Kassu R, Bogale G, Bekele M, Getnet M, Getachew A, et al. Magnitude and associated factors of poor medication adherence among diabetic and hypertensive patients visiting public health facilities in Ethiopia during the COVID-19 pandemic. PLoS One. 2021;16(4):e0249222.

97. Randeree H, Liebl A, Hajjaji I, Khamseh M, Zajdenverg L, Chen JW, et al. Safety and Effectiveness of Bolus Insulin Aspart in People with Type 2 Diabetes: A(1)chieve Sub-Analysis. Diabetes Ther. 2013;4(1):153–66.

98. Segal D, Tupy D, Distiller L. The Biosulin equivalence in standard therapy (BEST) study - a multicentre, open-label, non-randomised, interventional, observational study in subjects using Biosulin 30/70 for the treatment of insulin-dependent type 1 and type 2 diabetes mellitus. S Afr Med J [Internet]. 2013 [cited 2022 Feb 17];103(7):458–60. Available from: https://pubmed.ncbi.nlm.nih.gov/23802207/

99. Sobngwi E, Ndour-Mbaye M, Boateng KA, Ramaiya KL, Njenga EW, Diop SN, et al. Type 2 diabetes control and complications in specialised diabetes care centres of six sub-Saharan African countries: The Diabcare Africa study. Diabetes Res Clin Pract [Internet]. 2012 Jan [cited 2020 Apr 26];95(1):30–6. Available from: http://dx.doi.org/10.1016/j.diabres.2011.10.018

100. Tayo MS, Joshi MP, Jurgens JC, Hough et.al. GA, Distiller L, Centre for Diabetes and Endocrinology South Africa H. Achievement of therapeutic targets in South African patients with diabetes mellitus. Journal of Endocrinology, Metabolism and Diabetes of South Africa. South African Medical Association; 2017;22: 41–2.

101. Udoh B, Iwalokun B, Etukumana E, Amoo J. Asymptomatic falciparum malaria and its effects on type 2 diabetes mellitus patients in Lagos, Nigeria. Saudi J Med Med Sci. 2020;8(1):32.

102. Unung PJ, Bassey IE, Etukudo MH, Udoh AE, Alhassan MB, Akpan UO. Effect of glycemic control and dyslipidemia on plasma vascular endothelial growth factor and pigment epithelium-derived factor in diabetic retinopathy patients in Northern Nigeria. Int J Heal Sci. 2020;14(6):4–12.

103. van den Berg L, Mokhehle M, Raubenheimer J, University of the Free State SA. Nutritional status, glycaemic control and barriers to treatment compliance among patients with type 2 diabetes attending public primary health clinics in Maseru, Lesotho. J Endocrinol Metab Diabetes South Africa. 2019 Jan 1;24(3):98–110.

104. Woyesa SB, Hirigo AT, Wube TB. Hyperuricemia and metabolic syndrome in type 2 diabetes mellitus patients at Hawassa university comprehensive specialized hospital, South West Ethiopia. BMC Endocr Disord. 2017 Dec;17(1):76.

105. Yameogo N, Mbaye A, Kane A, Ndour M, Kagambega L, Ndiaye M, et al. Etude de la micro-albuminurie et des autres facteurs de risque cardio-vasculaire dans la population des diabétiques de type 2 sénégalais. Med Afr Noire. 2012;59(6):303–8.
